# Recent synthesis of thietanes

**DOI:** 10.3762/bjoc.16.116

**Published:** 2020-06-22

**Authors:** Jiaxi Xu

**Affiliations:** 1 State Key Laboratory of Chemical Resource Engineering, Department of Organic Chemistry, College of Chemistry, Beijing University of Chemical Technology, Beijing 100029, People’s Republic of China

**Keywords:** cycloaddition, cyclization, ring contraction, ring expansion, thietane, thiotherification

## Abstract

Thietanes are important aliphatic four-membered thiaheterocycles that are found in the pharmaceutical core and structural motifs of some biological compounds. They are also useful intermediates in organic synthesis. Various synthetic methods of thietanes have been developed, including inter- and intramolecular nucleophilic thioetherifications, photochemical [2 + 2] cycloadditions, ring expansions and contractions, nucleophilic cyclizations, and some miscellaneous methods. The recently developed methods provide some new strategies for the efficient preparation of thietanes and their derivatives. This review focuses on the synthetic methods to construct thietane backbones developed during 1966 to 2019.

## Review

### 1. Introduction

**Figure 1:**
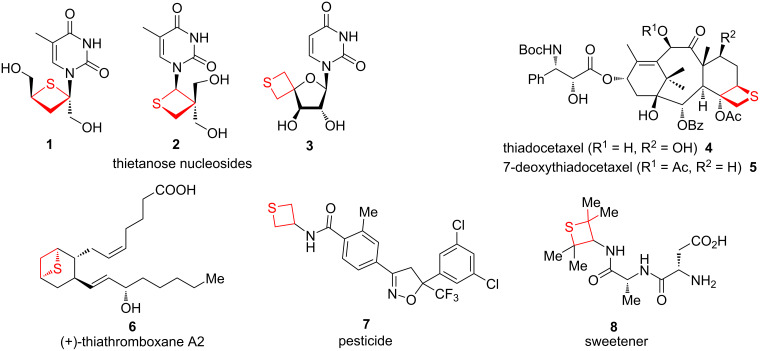
Examples of biologically active thietane-containing molecules.

**Figure 2:**
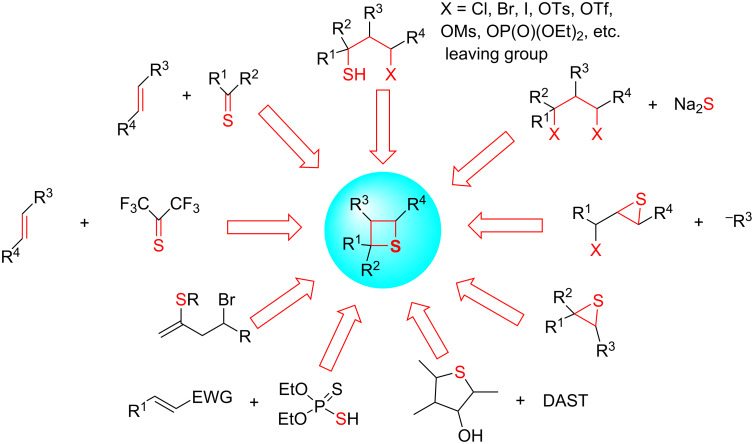
The diverse methods for the synthesis of thietanes.

Thietanes are a class of important aliphatic four-membered thiaheterocycles. Some simple alkyl and dialkyl thietanes are components of anal gland secretions of the stoat [1] and the ferret [2]. Some pharmaceutical and biological thietane-containing compounds include thiaanalogue thietanose nucleosides **1** and **2** [3–4], and the spiroannulated glyco-thietane nucleoside **3** [5] of the antiviral (anti-HIV and HSV) drug oxetanocin A, the D-ring-modified thia derivatives **4** and **5** of the anticancer drug taxoids and docetaxels [6], thiathromboxane A2 **6** [7], pesticide **7** [8], and the sweetener **8** [9] (Figure 1). Thietanes also serve as important and useful intermediates and versatile building blocks in organic synthesis for the preparation of sulfur-containing acyclic and heterocyclic compounds [10–11]. Several synthetic methods for thietanes have been developed and reviewed [12–14]. One traditional route is the intermolecular double substitution (cyclic thioetherification) of 1,3-dihaloalkanes, sulfonates of 3-haloalkan-1-ols, or disulfonates of alkane-1,3-diols with sodium sulfide. The intramolecular substitution of 3-mercaptoalkyl halides or sulfonates is a similar strategy for the preparation of thietanes [12–14]. Alternatively, inter- and intramolecular photochemical [2 + 2] cycloadditions (thia-Paternò–Büchi reactions) of alkenes and thiocarbonyl compounds are another important route for the synthesis of thietanes [15–16], especially, spirothietanes [17–18]. The formal [2 + 2] cycloadditions of hexafluorothioacetone and olefins are also applied in the preparation of bis(trifluoromethyl)-containing thietanes [19]. The ring-contractions of five and six-membered aliphatic thiaheterocycles have been seldom applied in the preparation of thiatetraoses [20–21]. In contrast, both nucleophilic and electrophilic ring expansions of thiiranes have been developed to synthesize thietanes [22–23]. Phosphorodithioate has been applied in the synthesis of thietanes as a nucleophile and generated phosphorothioate as a leaving group [24]. Some other cyclization methods have been reported in the synthesis of thietanes as well [25] (Figure 2).

This review covers the methods outlined in Figure 2 and also some miscellaneous methods for the synthesis of various thietane derivatives. A special focus is on the construction of the thietane ring, excluding methods for the simple modifications of the thietane rings and their side chains [26–31].

### 2. Synthesis via cyclic thioetherifications

#### 2.1 Synthesis via double nucleophilic displacements

**2.1.1 Synthesis via double nucleophilic displacements of 1,3-dihaloalkanes:** Although the double nucleophilic displacements of 1,3-dihaloalkanes with sodium sulfide are the oldest methods for the preparation of thietane derivatives and have well been studied, they are widely applied till now. The development of this method before 1965 was reviewed by Sander [12] and this review contains new advances since 1965.

**Scheme 1:**
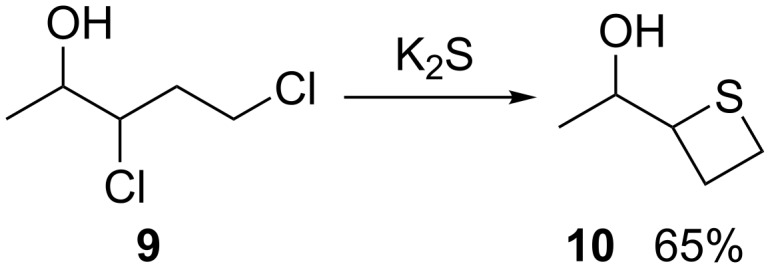
Synthesis of 1-(thietan-2-yl)ethan-1-ol (**10**) from 3,5-dichloropentan-2-ol (**9**).

After Sander’s review [12], Cerny and Polacek reported the synthesis of a thietane derivative via the double nucleophilic displacement of 1,3-dichloroalkane in 1966 [32]. They treated 3,5-dichloropentan-2-ol (**9**) with K_2_S to produce 1-(thietan-2-yl)ethan-1-ol (**10**) in 65% yield (Scheme 1).

**Scheme 2:**
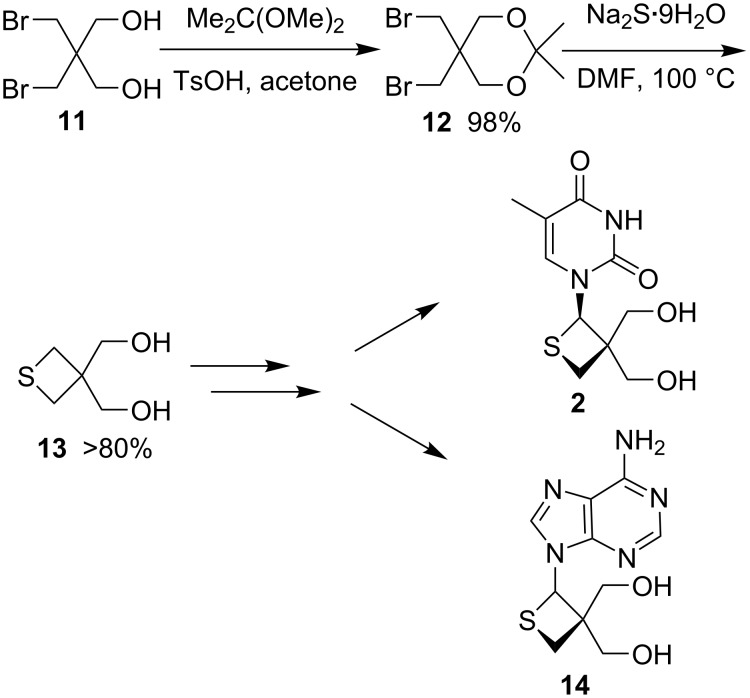
Synthesis of thietanose nucleosides **2**,**14** from 2,2-bis(bromomethyl)propane-1,3-diol (**11**).

In 2007, Nishizono and co-workers used 2,2-bis(bromomethyl)propane-1,3-diol (**11**) as starting material to prepare thietanose nucleosides **2** and **14**. They first carried out a double displacement with sodium sulfide to obtain thietane-3,3-diyldimethanol (**13**), which was further converted into two different thietanose nucleosides **2** and **14** [33] (Scheme 2).

**Scheme 3:**
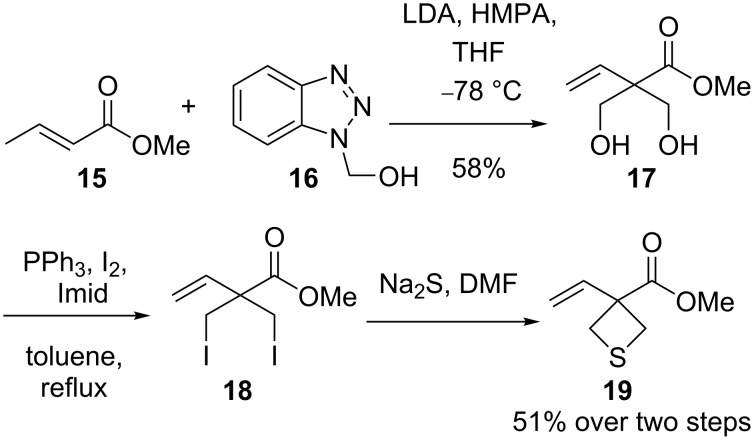
Synthesis of methyl 3-vinylthietane-3-carboxylate (**19**).

In the synthesis of sesquiterpene thioalkaloids, the method also was utilized. A double-aldol condensation of methyl crotonate (**15**) with 1-hydroxymethylbenzotriazole (**16**) generated methyl 2,2-dihydroxymethylbut-3-enoate (**17**) in 58% yield. Iodination and subsequent double displacement with sodium sulfide afforded methyl 1-vinylthietane-1-carboxylate (**19**) in 51% yield over two steps [34]. Compound **19** was used as an intermediate for the total synthesis of sesquiterpene thioalkaloids (Scheme 3).

**Scheme 4:**
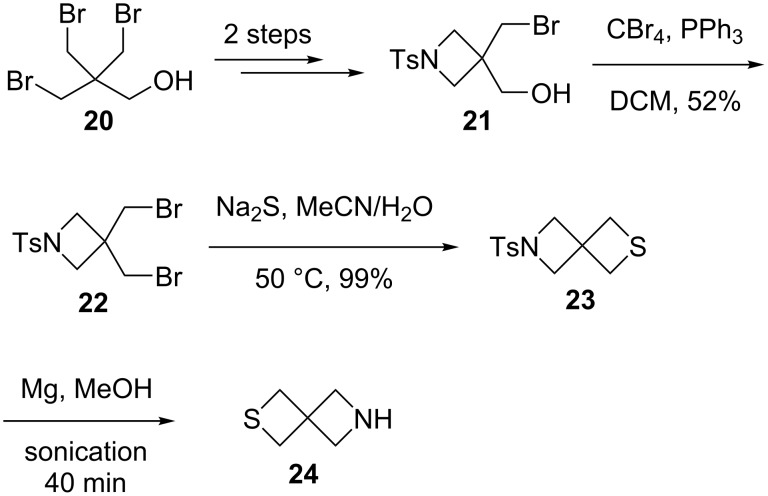
Synthesis of 1,6-thiazaspiro[3.3]heptane (**24**).

Spiro[3.3]heptane derivatives were recently used as the surrogates of piperazines, piperidines, morpholines, and thiomorpholines, which display pharmacological activities [35]. 1,6-Thiazaspiro[3.3]heptane (**24**) was synthesized for discovery of pan-CDK inhibitors. For this, 3-bromo-2,2-bis(bromomethyl)propan-1-ol (**20**) was transformed into 3-bromomethyl-3-hydroxymethyl-1-tosylazetidine (**21**), which was treated with Ph_3_P/CBr_4_ to yield 3,3-bis(bromomethyl)-1-tosylazetidine (**22**) in 52% yield. The double displacement of 3,3-bis(bromomethyl)-1-tosylazetidine (**22**) with sodium sulfide followed by the detosylation with Mg in MeOH afforded 1,6-thiazaspiro[3.3]heptane (**24**) [36] (Scheme 4).

**Scheme 5:**
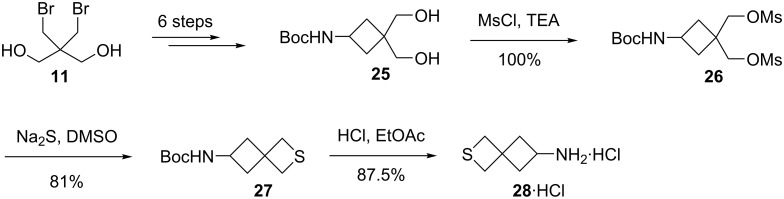
Synthesis of 6-amino-2-thiaspiro[3.3]heptane hydrochloride (**28**).

**2.1.2 Synthesis via double nucleophilic displacements of disulfonates of alkane-1,3-diols:** Considering that 6-amino-3-azaspiro[3.3]heptane was evaluated as inhibitor of kinases, insecticides, and acaricides, its sulfur analogue, 6-amino-2-thiaspiro[3,3]heptane (**28**) was prepared from the cheap starting material 2,2-bis(bromomethyl)propane-1,3-diol (**11**). Compound **11** was converted into 3-(*tert*-butoxycarbonyl)-1,1-bis(hydroxymethyl)aminocyclobutane (**25**) in 6 steps. After the treatment of **25** with methanesulfonyl chloride, the obtained dimethanesulfonate **26** was reacted with sodium sulfide giving rise to 6-(*tert*-butoxycarbonyl)amino-2-thiaspiro[3.3]heptane (**27**), which was further transformed into the desired 6-amino-2-thiaspiro[3,3]heptane (**28**) hydrogen chloride salt after the acidic deprotection [37] (Scheme 5).

**Scheme 6:**
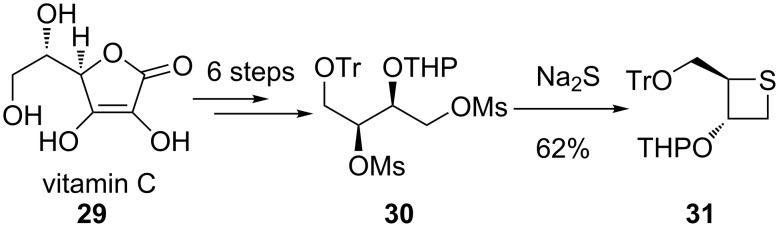
Synthesis of optically active thietane **31** from vitamin C.

During recent decades, the cyclic thioetherification strategy was widely applied in the synthesis of thietane-based square sugurs (thietanoses), and sulfur-containing glycomimetics of furanoses and pyranoses [38]. The first thietanose was synthesized from vitamin C (**29**) in 1996 (Scheme 6). Vitamin C (**29**) was converted first into 1,3-dimesylate **30** of 2,4-di-*O*-protected 1,2,3,4-butane-tetraol in 6 steps. The subsequent treatment with Na_2_S in refluxing ethanol then gave rise to the protected thietanose **31** in 62% yield [3] (Scheme 6).

**Scheme 7:**
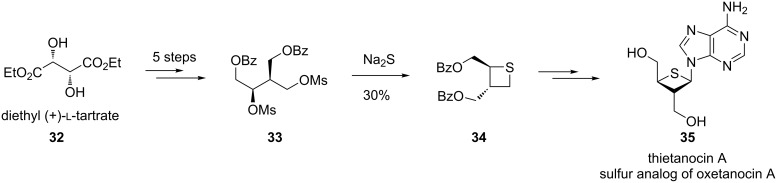
Synthesis of an optically active thietane nucleoside from diethyl L-tartrate (**32**).

Following similar protocols, (*S*,*S*)-2,3-bis(benzoyloxymethyl)thietane (**34**) was synthesized from diethyl L-tartrate (**32**), which was further converted into thietanocin A (**35**), a sulfur analogue of oxetanocin A [39] (Scheme 7).

**Scheme 8:**
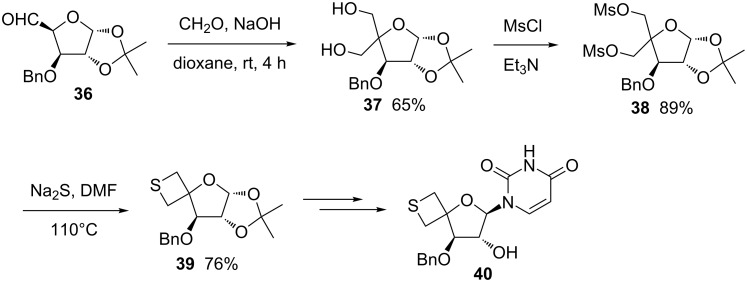
Synthesis of thietane-containing spironucleoside **40** from 5-aldo-3-*O*-benzyl-1,2-*O*-isopropylidene-α-D-glucofuranose (**36**).

The double displacement cyclic thioetherification strategy was also utilized for the synthesis of thietane-containing spironucleosides. The easily available 5-aldo-3-*O*-benzyl-1,2-*O*-isopropylidene-α-D-glucofuranose (**36**) was first treated with formaldehyde in the presence of NaOH followed by MsCl, affording the dimesylate derivative **38**, which was reacted with Na_2_S to afford the spirothietane **39**. The latter was further converted into the thietane-containing spironucleoside **40** [40] (Scheme 8).

**Scheme 9:**
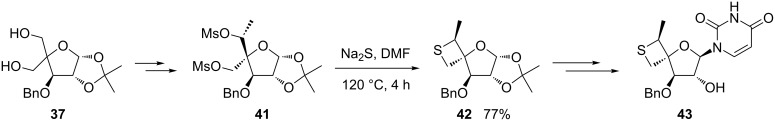
Synthesis of optically active 2-methylthietane-containing spironucleoside **43**.

The same research group synthesized the optically active 2-methylthietane-containing spironucleoside **43** by following a similar synthetic method [40] (Scheme 9).

**Scheme 10:**
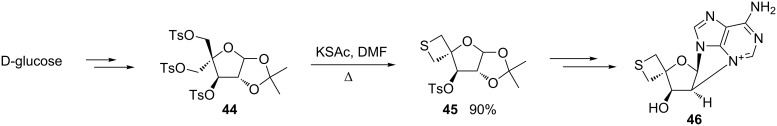
Synthesis of a double-linked thietane-containing spironucleoside **48**.

In 2009, Da Silva and co-worker succeeded in the synthesis of a 4’,4’-spirothietane-2’,*N*^3^-cycloadenosine **46** as a highly constrained analogue of 5’-deoxy-5’-methylthioadenosine. They first prepared tritosylate derivative **44** from D-glucose which was treated with KSAc to give the spirothietane derivative **45**. The latter compound was further converted to the final thietane-containing spironucleoside **46** [41] (Scheme 10).

**Scheme 11:**
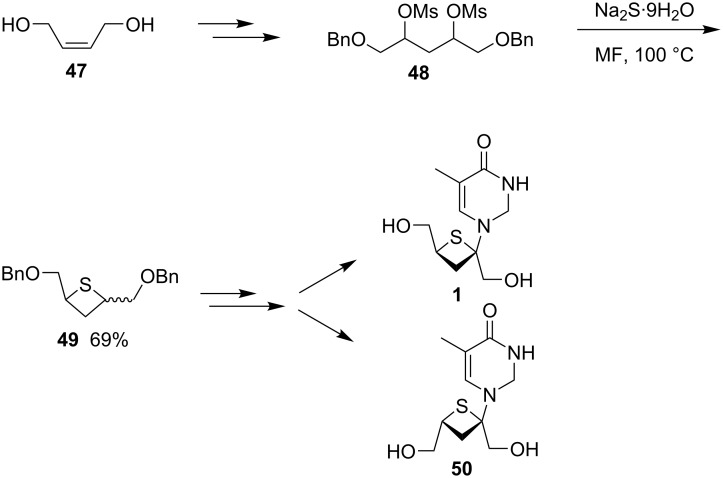
Synthesis of two diastereomeric thietanose nucleosides via 2,4-di(benzyloxymethyl)thietane (**49**).

In 2011, Nishizono and co-worker synthesized two anomeric thietanose nucleosides with (*Z*)-but-2-ene-1,4-diol (**47**) as the starting material. They first converted the diol **47** into dimethanesulfonates **48** of 1,5-dibenzyloxypentane-2,4-diol and treated it with sodium sulfide to afford 2,4-di(benzyloxymethyl)thietane (**49**). Compound **49** was then further transformed into two different anomeric thietanose nucleosides **1** and **50** [4] (Scheme 11).

**Scheme 12:**
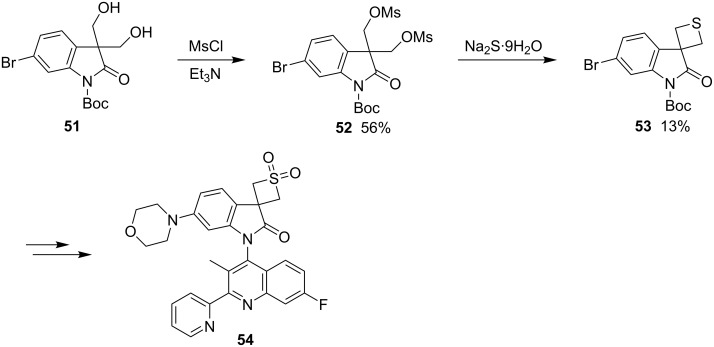
Synthesis of the thietane-containing PI3k inhibitor candidate **54**.

In the development of novel class I phosphoinositide 3-kinase (PI3k) inhibitors, 6-bromo-3,3-bis(hydroxymethyl)indolin-2-one (**51**) was reacted first with mesyl chloride and then treated with sodium sulfide to afford 6-bromospiro[indoline-3,3′-thietan]-2-one (**53**), which was further converted into the target inhibitor candidate **54** [42] (Scheme 12).

**Scheme 13:**
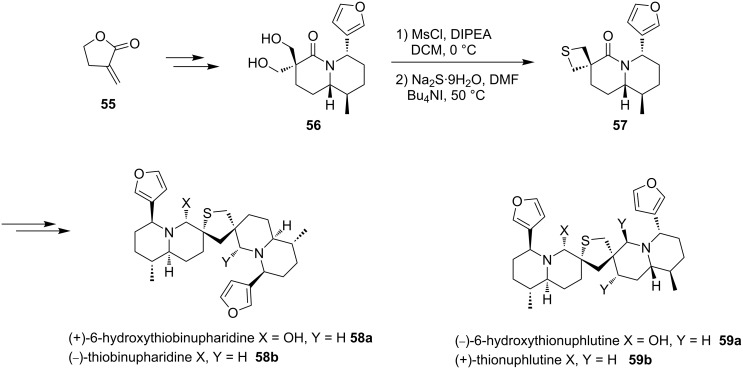
Synthesis of the spirothietane **57** as the key intermediate to Nuphar sesquiterpene thioalkaloids.

2-Methylene-γ-butyrolactone (**55**) as the initial starting material was converted into bis(hydroxymethyl)quinolizidinone **56**. After mesylation and the double displacement with sodium sulfide, spirothietane-quinolizidine **57** was obtained as a key intermediate. It was further applied in the total synthesis of four different natural products of Nuphar sesquiterpene thioalkaloids **58** and **59** [43] (Scheme 13).

#### 2.2. Synthesis via intramolecular nucleophilic displacements

**Scheme 14:**
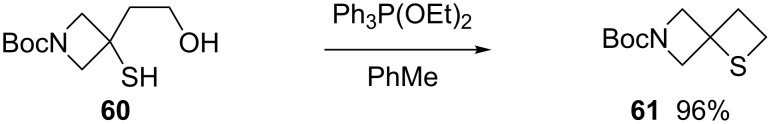
Synthesis of spirothietane **61** through a direct cyclic thioetherification of 3-mercaptopropan-1-ol.

**2.2.1 Synthesis via the direct cyclic thioetherification of γ-mercaptoalkanols:** The direct cyclic thioetherification of γ-mercaptoalkanols was regarded as an efficient route to synthesize thietanes. Indeed, the direct cyclization of the 3-mercaptopropan-1-ol unit in **60** with Ph_3_P(OEt)_2_ as a reagent was realized in the synthesis of the spirothietane derivative **61** [44] (Scheme 14).

**Scheme 15:**
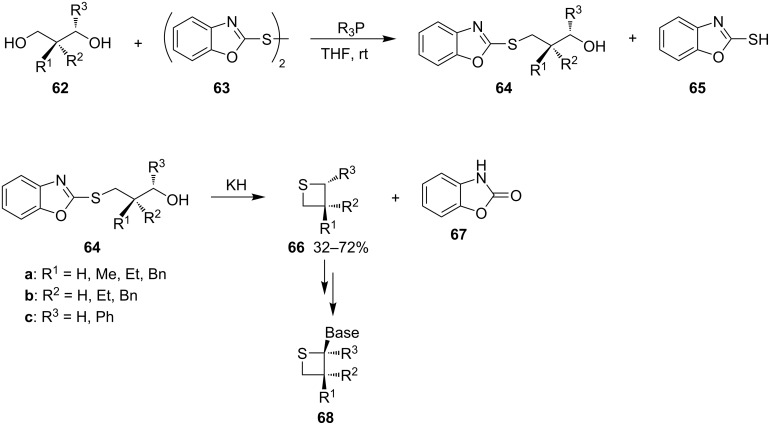
Synthesis of thietanes **66** from 1,3-diols **62**.

Also, 1,3-diols were considered as precursors of γ-mercaptoalkanols. A Japanese group developed a new method to transform 1,3-diols **62** into the precursors of γ-mercaptoalkanols with dibenzoxazol-2-yl disulfide (**63**) and phosphines. They reacted primary or secondary 1,3-diols **62** with disulfide **63** in the presence of Bu_3_P or Ph_3_P to selectively synthesize 2-(3-hydroxyalkylthio)benzoxazoles **64.** These were treated with KH to afford the corresponding thietanes **66**. The subsequent introduction of nucleobases then gave the corresponding thietanose nucleosides **68** [45] (Scheme 15).

**Scheme 16:**
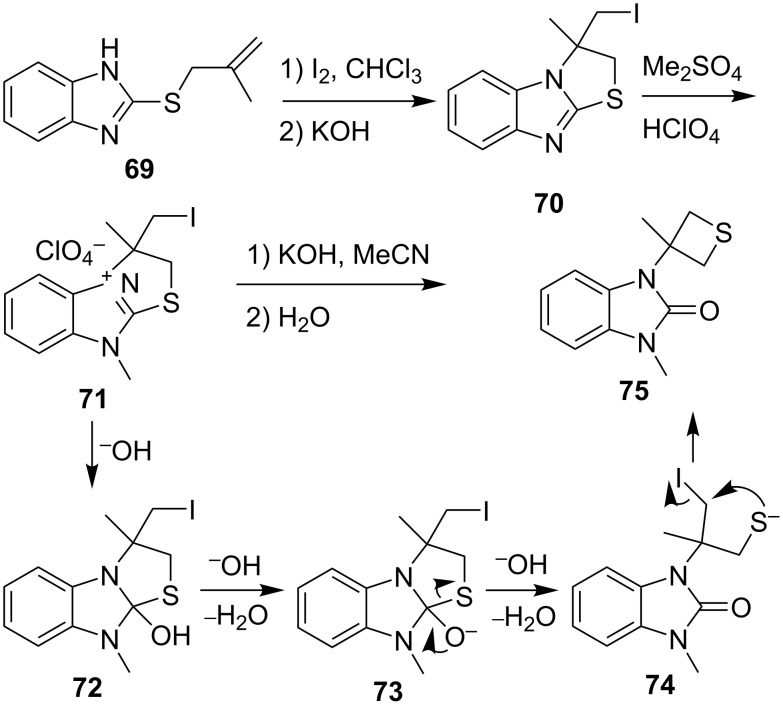
Synthesis of thietanylbenzimidazolone **75** from (iodomethyl)thiazolobenzimidazole **70**.

The treatment of 2-(allylthio)benzimidazole **69** with iodine in CHCl_3_ followed by aq. KOH gave (iodomethyl)thiazolobenzimidazole **70** which was converted to thiazolobenzimidazolium perchlorate **71** by methylation with dimethyl sulfate and addition of HClO_4_. After the treatment with KOH powder in MeCN and subsequent hydrolysis it gave thietanylbenzimidazolone **75**. In the last step, the hydroxide ion first nucleophilically added to the iminium **71** to generate an *O,S*-hemiacetal **72**. Under the basic conditions, the hemiacetal **72** converted to the thiolate **74**, which underwent an intramolecular substitution to give the final product thietanylbenzimidazolone **75** [46] (Scheme 16).

**Scheme 17:**
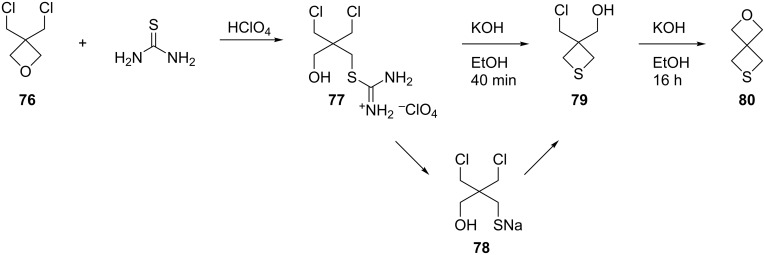
Synthesis of 2-oxa-6-thiaspiro[3.3]heptane (**80**) from bis(chloromethyl)oxetane **76** and thiourea.

**2.2.2 Synthesis via the stepwise nucleophilic displacements:** Besides the double displacements of 1,3-dihaloalkanes with different sulfide salts, thiourea was also used as a nucleophile in the double displacements, actually following the preparation procedure of thiols, affording thietane derivatives. Thiourea reacted with 3,3-bis(chloromethyl)oxetane (**76**) in the presence of HClO_4_ to yield *S*-[2-(3-chloro-2-(chloromethyl)-2-hydroxymethyl)propyl]isothiouronium perchlorate (**77**). Heating compound **77** with KOH in ethanol for 40 min yielded 3-chloromethyl-3-hydroxymethylthietane (**79**) through a thiolate intermediate (**78**). Further reflux for 16 h gave rise to 2-oxa-6-thiaspiro[3.3]heptane (**80**) [47] (Scheme 17).

**Scheme 18:**
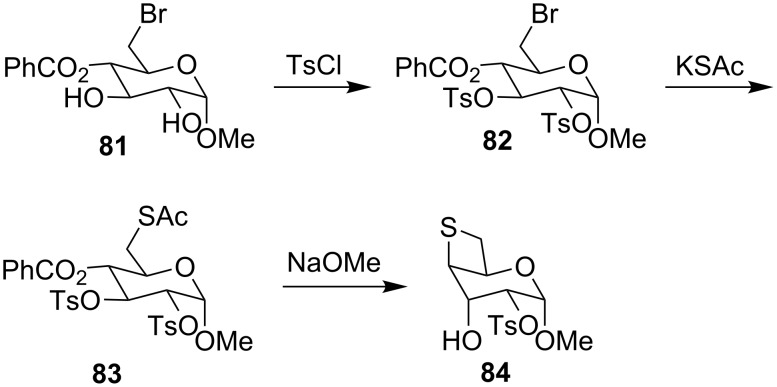
Synthesis of the thietane-containing glycoside, 2-*O*-*p*-toluenesulfonyl-4,6-thioanhydro-α-D-gulopyranoside (**84**).

In 1985, Miljkovic and co-workers reported the synthesis of thioanhydrohexopyranosides starting from bromodeoxyglucopyranoside **81**. Compound **81** was reacted with *p*-MeC_6_H_4_SO_2_Cl and KSAc to yield thioacetate **83**, that upon treatment with excess NaOMe, gave methyl 2-*O*-*p*-toluenesulfonyl-4,6-thioanhydro-α-D-gulopyranoside (**84**), the thietane-containing gulopyranoside [48] (Scheme 18).

**Scheme 19:**
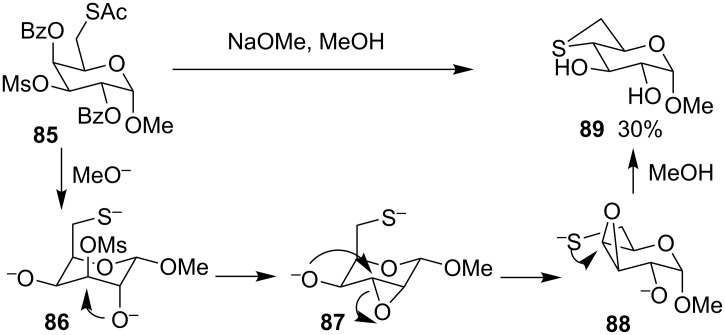
Synthesis of methyl 4,6-thioanhydro-α-D-glucopyranoside (**89**).

For the preparation of thioanhydro sugar derivatives, Cubero and co-workers treated methyl 6-*S*-acetyl-2,4-di-*O*-benzoyl-3-*O*-methanesulfonyl-6-thio-α-D-galactopyranoside (**85**) with methanolic sodium methoxide to generate methyl 4,6-thioanhydro-α-D-glucopyranoside (**89**), the thietane-fused pyranoside, in 30% yield [49] (Scheme 19).

**Scheme 20:**
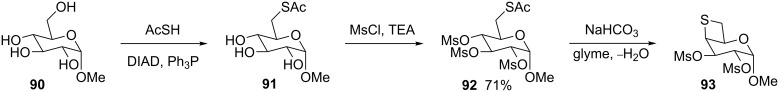
Synthesis of thietane-fused α-D-galactopyranoside **93**.

Since 2000, a lot of thietane-derived carbohydates were reported. Voss and co-workers prepared the 2-oxo-7-thiabicyclo[4.2.0]octane derivative (methyl 2,3-di-*O*-mesyl-4,6-thio-anhydro-α-D-galactopyranoside (**93**)) from methyl α-D-glucopyranoside (**90**) through a Mitsunobu thioacetylation, mesylation, thioacetate hydrolysis with the treatment of sodium bicarbonate, and a subsequent intramolecular nucleophilic displacement. In the displacement step, the formation of the four-membered thietane ring is strongly favored over the ring closure between the thiolate and the 2-position, since the S_N_2 displacement of a mesylate leaving group adjacent to the anomeric center is known to be restricted [50] (Scheme 20).

**Scheme 21:**
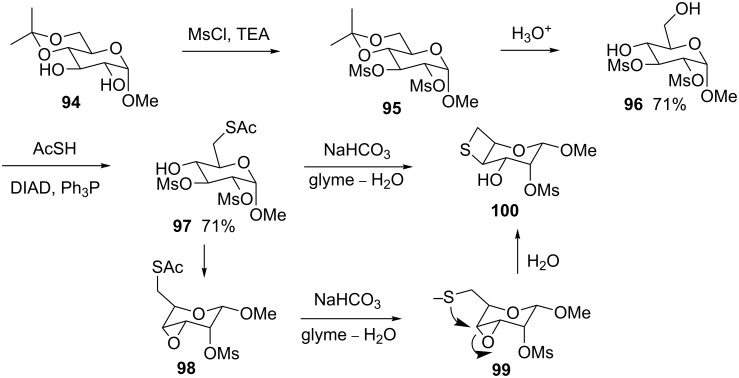
Synthesis of thietane-fused α-D-gulopyranoside **100**.

The same group synthesized a thietane-fused gulopyranoside starting from methyl 4,6-*O*-isopropylidene-α-D-glucopyranoside (**94**). Compound **94** first was mesylated and then hydrolyzed to afford 2,3-dimesylated methyl α-D-glucopyranoside **96**. After thioacetylation and treatment with sodium bicarbonate compound **96** was converted into the thietane-fused α-D-gulopyranoside **100**. The thioacetate derivative **97** was first converted to the oxirane-fused derivative **98** through an intramolecular substitution. After hydrolysis, the thiolate underwent an intramolecular nucleophilic displacement to generate the final thietane-fused α-D-gulopyranoside **100** [50] (Scheme 21).

**Scheme 22:**
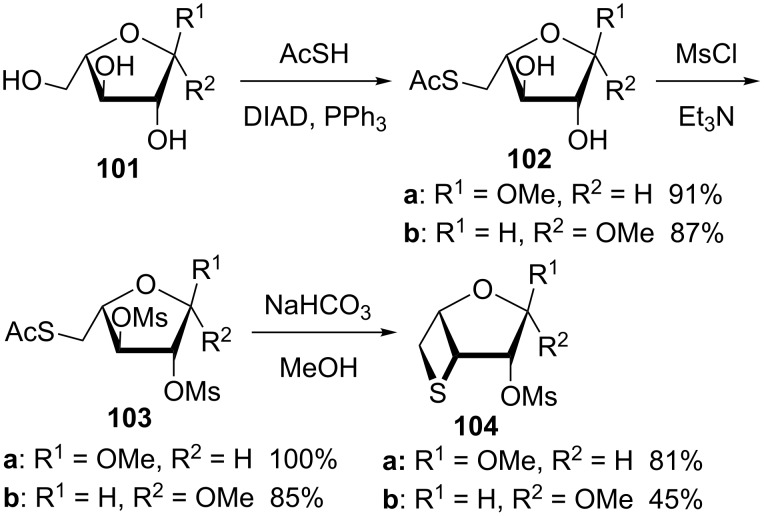
Synthesis of 3,5-anhydro-3-thiopentofuranosides **104**.

In 2004, Schulze and co-workers synthesized 3,5-anhydro-3-thiopentofuranosides **104** from methyl α- and β-arabinosides **101** through a Mitsunobu reaction, mesylation, and hydrolysis sequence followed by an intramolecular displacement. The in situ generated thiolate nucleophilically attacked the mesylate to form the thietane ring [51] (Scheme 22).

**Scheme 23:**
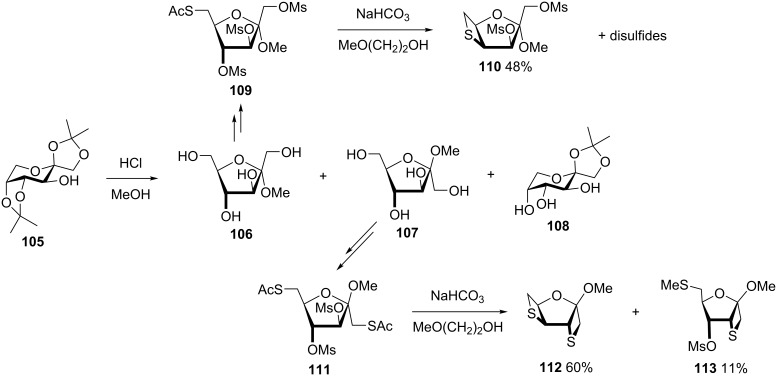
Synthesis of anhydro-thiohexofuranosides **110**, **112** and **113** from from 1,2:4,5-di-*O*-isopropylidene D-fructose (**105**).

Following the similar synthetic route, Polchow and Voss synthesized 4,6-anhydro-4-thiofuranoside **110**, 1,3:4,6-dianhydro-1,4-dithio-β-D-sorbofuranoside **112**, and 1,3-anhydro-6-*S*-methyl-1,6-dithio-D-psicofuranoside **113** from 1,2:4,5-di-*O*-isopropylidene D-fructose (**105**) [52] (Scheme 23).

**Scheme 24:**
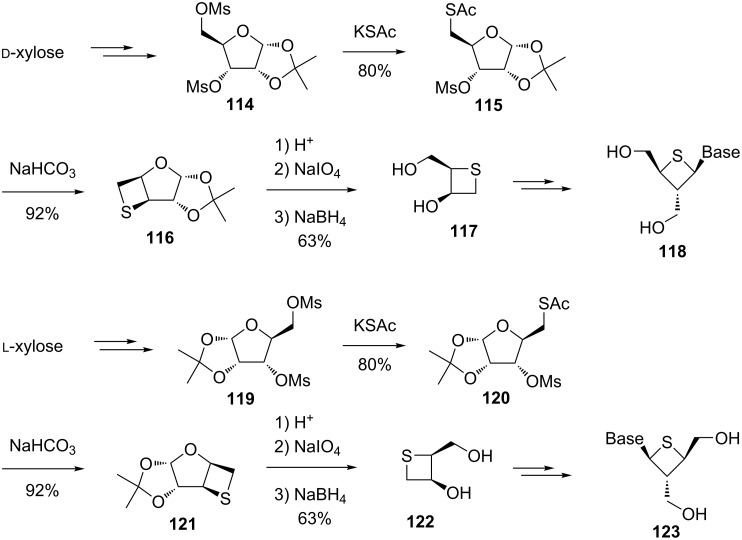
Synthesis of optically active thietanose nucleosides from D- and L-xyloses.

In an alternative approach, the thietane ring was constructed more efficiently through a two-step displacement sequence from the D-xylose-derived dimesylate **114** (Scheme 24). The first step displacement involved the selective S_N_2 reaction of the primary mesylate with KSAc to yield a monothioacetate **115** in 80% yield. The second displacement was an intramolecular S_N_2 process performed under mild basic conditions, affording the desired thietane **116** in 92% yield. After deprotection, oxidative cleavage, and reduction, a thietanose **117** was obtained in 63% overall yield. The thietanose **117** was further applied to synthesize a series of thietanose nucleosides **118** [53]. Similarly, enatiomeric thietanose nucleosides **123** were prepared from L-xylose [53] (Scheme 24).

**Scheme 25:**
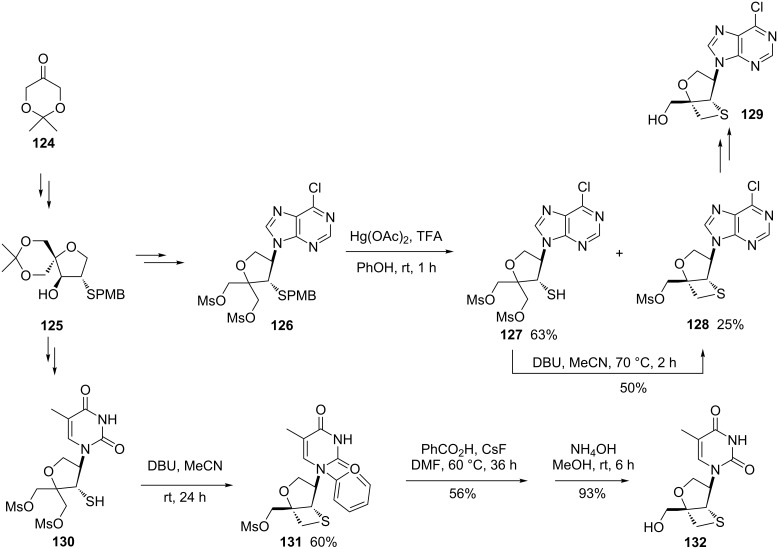
Synthesis of thietane-fused nucleosides.

In 2010, Takahata and co-workers designed and synthesized thietane-fused nucleosides. They first prepared a key intermediate spiro acetal **125**, which was converted into two different dimesylated nucleosides. After the deprotection with Hg(OAc)_2_ in the presence of TFA, the dimesylated thiols **127** and **130** generated companied with the thietane-fused nucleoside **128** in one case. Further the treatment of the dimesylated thiols **127** and **130** with DBU gave rise to the corresponding mesylated thietane-fused nucleosides **128** and **131**, which generated the final thietane-fused nucleosides **129** and **132** after the reactions with benzoic acid and CsF and subsequent aminolysis [54] (Scheme 25).

**Scheme 26:**
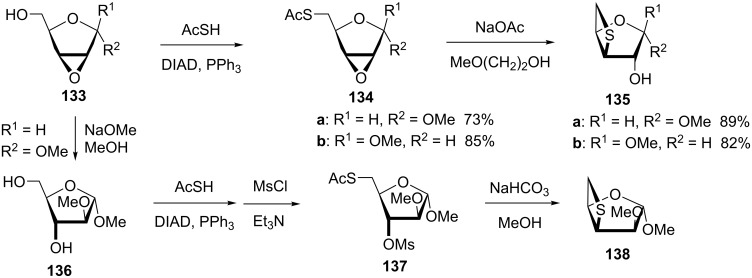
Synthesis of 3,5-anhydro-3-thiopentofuranosides.

The methyl 2,3-anhydro-α- and β-D-ribofuranosides **133** were used as starting materials and converted into 3,5-anhydro-3-thiopentofuranosides **135** through a Mitsunobu reaction with thiolacetic acid and hydrolysis followed by an intramolecular nucleophilic ring-opening of the oxirane ring. The newly generated thiolate underwent a nucleophilic ring-opening of the oxirane to generate the thietane ring [55] (Scheme 26).

After the ring-opening of methyl 2,3-anhydro-α-D-ribofuranoside (**133a**) with NaOMe, following a sequence of a Mitsunobu reaction, mesylation, and treatment with sodium bicarbonate, another 3,5-anhydro-3-thiopentofuranoside **138** was prepared [51] (Scheme 26).

**Scheme 27:**
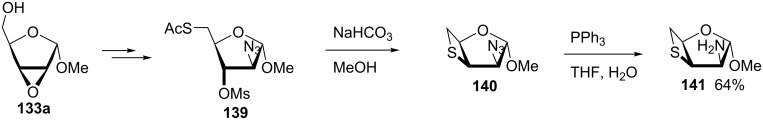
Synthesis of 2-amino-3,5-anhydro-3-thiofuranoside **141**.

The 2-amino-3,5-anhydro-3-thiofuranoside **141** was prepared from methyl 2,3-anhydro-α-D-ribofuranoside (**133a**), which was first reacted with sodium azide followed by the similar synthetic route as described above, affording 3,5-anhydro-2-azido-3-thiofuranoside **139**. The azido derivative **139** generated the final product 2-amino-3,5-anhydro-3-thiofuranoside **141** by reduction with triphenylphosphine [55] (Scheme 27).

**Scheme 28:**
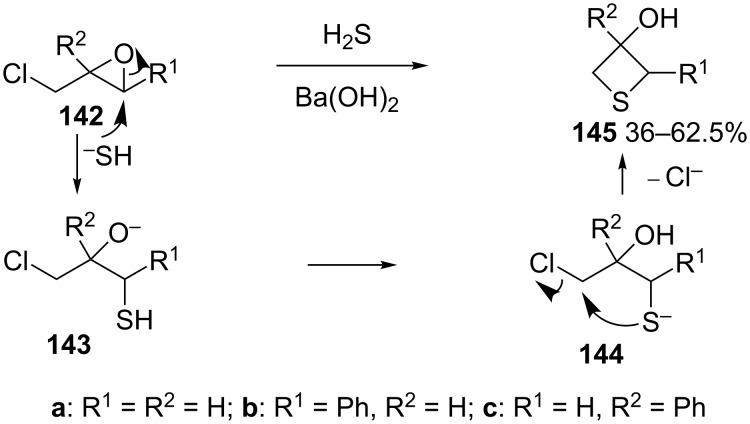
Synthesis of thietane-3-ols **145** from (1-chloromethyl)oxiranes **142** and hydrogen sulfide.

**2.2.3 Synthesis via the nucleophilic ring-opening of three-membered heterocycles and subsequent displacement from halomethyloxirane derivatives:** Chloromethyloxirane (**142a**) and its 2 and 3-phenyl derivatives **142b** and **142c** reacted with H_2_S in the presence of Ba(OH)_2_ to give the corresponding thietane-3-ols **145**. In this reaction H_2_S first was deprotonated to the hydrogensulfide anion (^−^SH) by Ba(OH)_2_. The obtained anion nucleophilically attacked the less steric or benzylic ring carbon atom of the oxirane ring, giving mercaptoalkanolates **143**. A proton transfer generated hydroxyalkanethiolates **144** because the acidity of the thiols is higher than that of alcohols, the newly generated thiolates **144** underwent an intramolecularly nucleophilic displacement to give thietane-3-ols **145** [56] (Scheme 28).

**Scheme 29:**
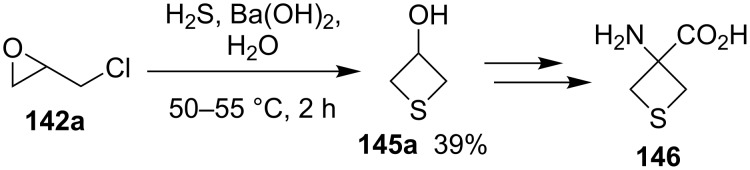
Synthesis of thietane-3-ol **145a** from chloromethyloxirane (**142a**).

In a similar approach, chloromethyloxirane (**142a**) was first converted into a thietan-3-ol **145a** by treatment with H_2_S and Ba(OH)_2_. Compound **145a** was further transformed to 3-aminothietane-3-carboxylic acid (**146**), a modulator of the *N*-methyl-D-aspartate (NMDA) receptor [57] (Scheme 29).

**Scheme 30:**
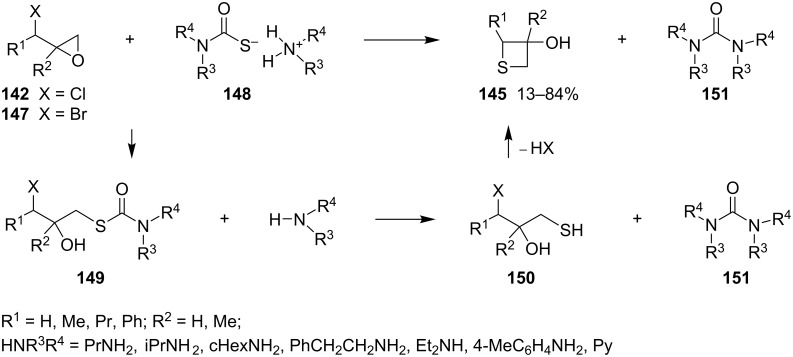
Synthesis of thietane-3-ols **145** from 2-(1-haloalkyl)oxiranes **142** and **147** with ammonium monothiocarbamates **148**.

Several thietane-3-ol derivatives **145** were synthesized in low to good yields by the reaction of 2-(1-haloalkyl)oxiranes **142** and **147** with ammonium monothiocarbamates **148** as the sulfur nucleophiles. First, a nucleophilic ring-opening of the oxiranes **142** and **147** by monothiocarbamates **148** gave rise to the *S*-(γ-halo-β-hydroxyalkyl)carbamates **149** with release of amines. The latter then aminolyzed the carbamates **149** to generate ureas **151** and γ-halo-β-hydroxyalkanethiols **150**. The intermediates **150** further underwent an intramolecular cyclization to produce the thietane-3-ols **145** in low to good yields [58] (Scheme 30).

**Scheme 31:**
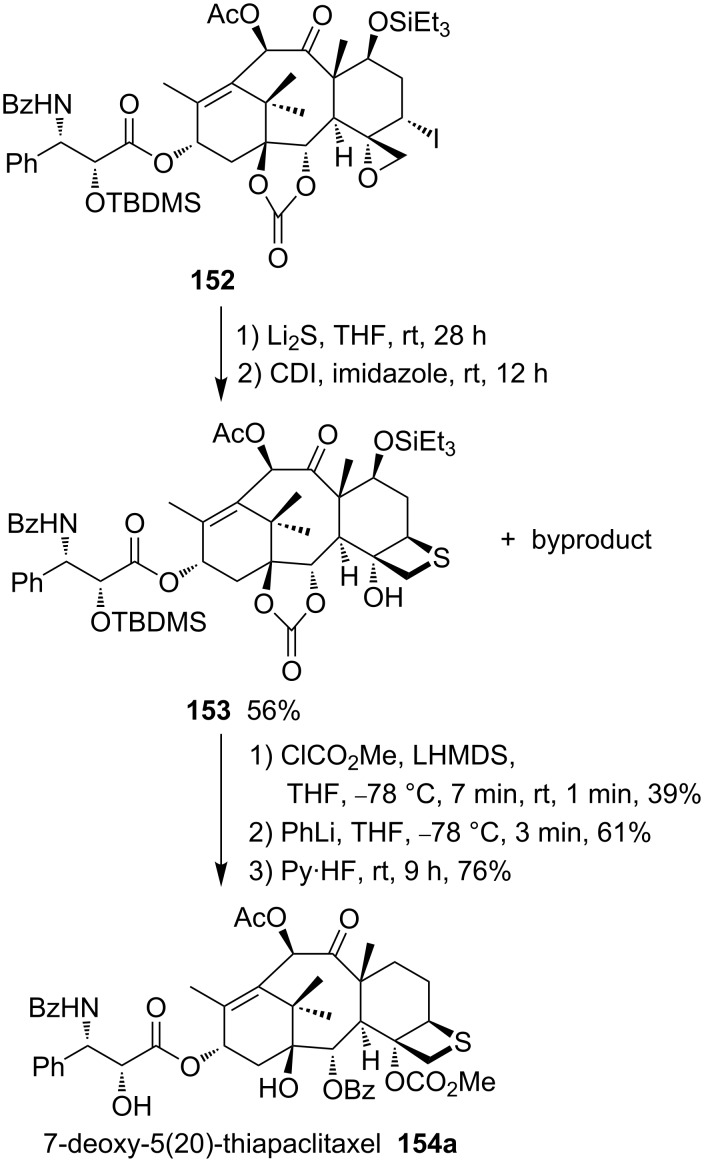
Synthesis of 7-deoxy-5(20)thiapaclitaxel **154a**, a thietane derivative of taxoids.

Paclitaxel (Taxol^®^) and docetaxel (Taxotere^®^) both are anticancer drugs of the taxoid series. They inhibit cell growth through the interaction with microtubules. In order to study the structure–activity relationships, the D-ring-modified deoxythiataxoid **154a** was synthesized. For this, the iodomethyloxirane derivative **152** was first treated with lithium sulfide followed by reaction with carbonyldiimazole (CDI), yielding the thietane derivative **153** and byproduct. The thietane derivative **153** was then converted into 7-deoxy-5(20)-thiapaclitaxel **154a** in a three steps sequence [59] (Scheme 31).

**Scheme 32:**
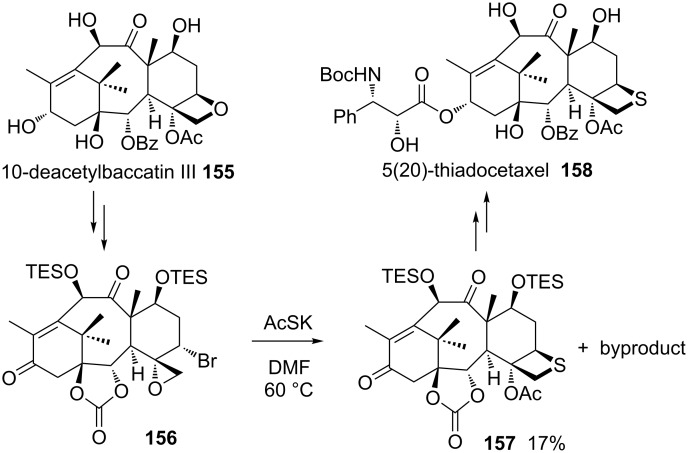
Synthesis of 5(20)-thiadocetaxel **158** from 10-deacetylbaccatin III (**155**).

Another member of taxoids, 10-deacetylbaccatin III (**155**) was isolated from the leaves of the European yew tree *Taxus baccata L.* in a significant yield and was applied as starting material for the semisynthesis of 5(20)-thiadocetaxel **158**. First, the compound was converted into the corresponding bromomethyloxirane derivative **156**, which generated the corresponding thietane-fused product **157** by the treatment with KSAc. Product **157** was finally transformed to 5(20)-thiadocetaxel **158** [6] (Scheme 32).

**Scheme 33:**
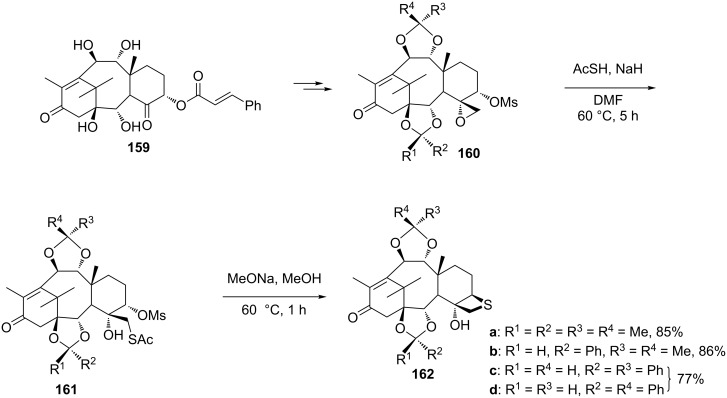
Synthesis of thietane derivatives **162** as precursors for deoxythiataxoid synthesis through oxiranemethyl mesylate derivatives **160**.

**2.2.4 Synthesis via the nucleophilic ring-opening of three-membered heterocycles and subsequent displacement from oxirane-2-methyl sulfonates:** Similar as for the halomethyloxirane derivatives, oxiranemethyl mesylate derivatives were also used as precursors for the synthesis of the corresponding thietane derivatives. After various protection–deprotection steps and mesylation, the oxiranemethyl mesylate derivatives **160** were prepared (Scheme 33). Following treatments with KSAc and NaOMe in methanol, respectively, the corresponding thietane-fused products **162** were obtained as the intermediates for the synthesis of deoxythiataxoids [60] (Scheme 33).

**Scheme 34:**
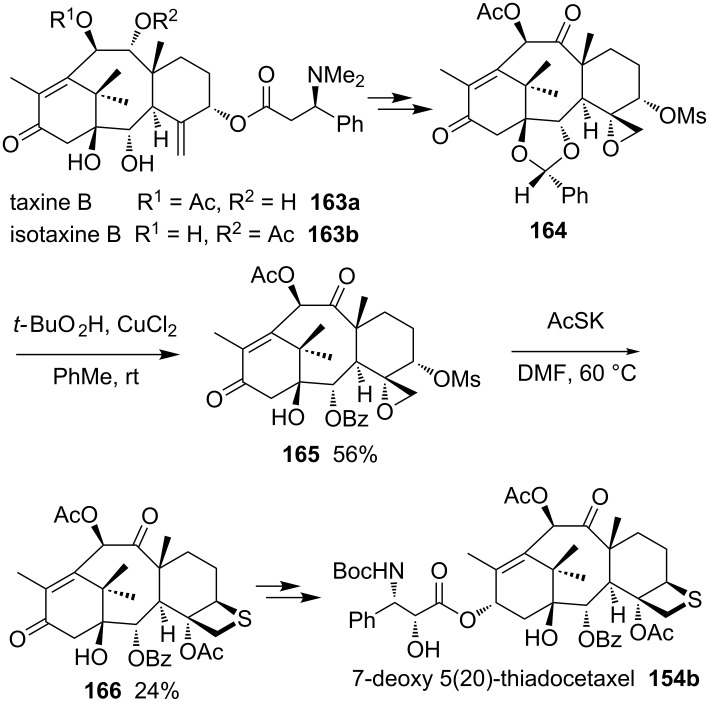
Synthesis of 7-deoxy 5(20)-thiadocetaxel **154b**.

Taxine B (**163a**) and isotaxine B (**163b**) were obtained from the leaves of the European yew tree *Taxus baccata L.* in significant yields as well. The compounds were used for the semisynthesis of further sulfur derivatives of taxoids by first converting them into the acetal-protected oxiranemethyl mesylate derivative **164**. After the treatment of compound **164** with KSAc, the mesylate **165** generated the corresponding thietane-fused product **166**, which was finally converted into the D-ring-modified 7-deoxy 5(20)-thiadocetaxel **154b** [6] (Scheme 34).

**Scheme 35:**
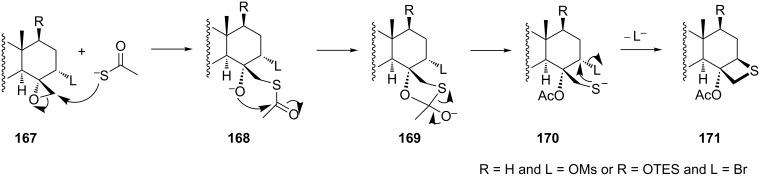
Mechanism for the formation of the thietane ring in **171** from oxiranes with vicinal leaving groups **167**.

The mechanism for the formation of thietane rings **171** from oxiranes **167** with vicinal leaving groups was suggested as a nucleophilic ring-opening and intramolecular transesterification followed by an intramolecular displacement [6] (Scheme 35).

**Scheme 36:**
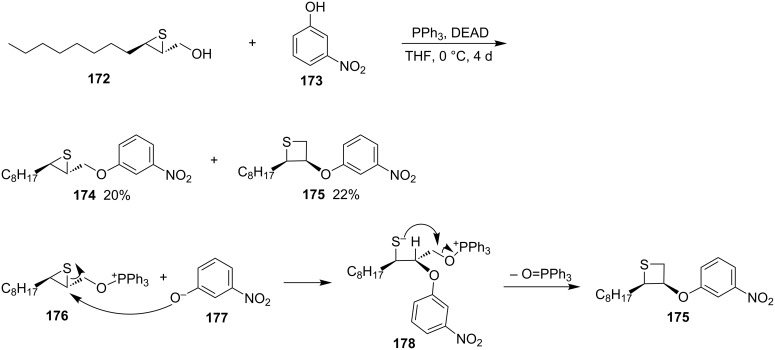
Synthesis of *cis-*2,3-disubstituted thietane **175** from thiirane-2-methanol **172**.

**2.2.5 Synthesis via the nucleophilic ring-opening of three-membered heterocycles and subsequent displacement from thiirane-2-methanol derivatives:** Gay and Scherowsky prepared thietane derivatives from a thiirane-2-methanol when they worked on the synthesis of liquid crystal materials. They synthesized a chiral thietane **175** from the chiral thiirane-2-methanol **172** with 3-nitrophenol (**173**) under Mitsunobu conditions (Scheme 36). In the synthesis, the alcohol **172** first reacted with triphenylphosphine to generate thiirane **174**, which underwent nucleophile ring-opening followed by an intramolecular substitution to afford chiral thietane **175** [61].

**Scheme 37:**
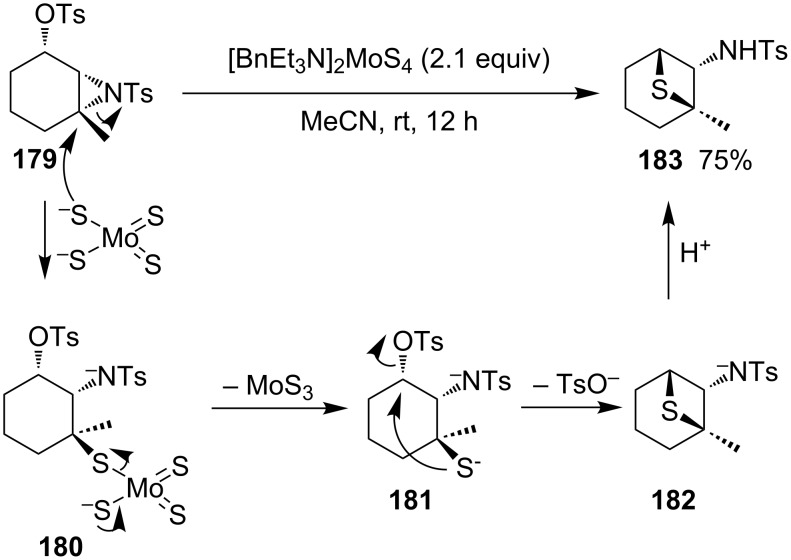
Synthesis of a bridged thietane **183** from aziridine cyclohexyl tosylate **179** and ammonium tetrathiomolybdate.

**2.2.6 Synthesis via the nucleophilic ring-opening of three-membered heterocycles and subsequent displacement from aziridine-2-methyl tosylate:** (1*R*,2*S*,6*R*)-6-Methyl-7-tosyl-7-azabicyclo[4.1.0]heptan-2-yl tosylate (**179**) is a derivative of aziridine-2-methyl tosylate. After the ring-opening with ammonium tetrathiomolybdate and subsequent intramolecular cyclization, the compound was converted into a bridged thietane **183** in 75% yield. The results indicated that, in the ring-opening step, tetrathiomolybdate nucleophilically attacked the more substituted aziridine carbon atom [62] (Scheme 37).

The thioetherification cyclization of 1,3-dihaloalkanes, 3-haloalkyl sulfonates, or disulfonates of alkane-1,3-diols with sodium sulfide is a common method for the preparation of thietanes. However, this method is suitable for the preparation of 3-monosubstituted and 3,3-disubstituted thietanes but can hardly applied for the preparation of 2,2/2,4-disubstituted, 2,2,4-trisubstituted, and 2,2,4,4-tetrasubstituted thietanes due to steric hindrance in the substitution step. In these cases, the substitution reaction is accompanied by elimination reactions.

### 3. Synthesis via cycloadditions

Cycloadditions, especially the photochemical [2 + 2] cycloaddition (thia-Paternò–Büchi reaction) of thiones and thioamides with olefins [15–18], and formal cycloadditions are alternative routes for the construction of thietane derivatives, especially multisubstituted thietanes.

#### 3.1 Synthesis via photochemical [2 + 2] cycloadditions

**Scheme 38:**
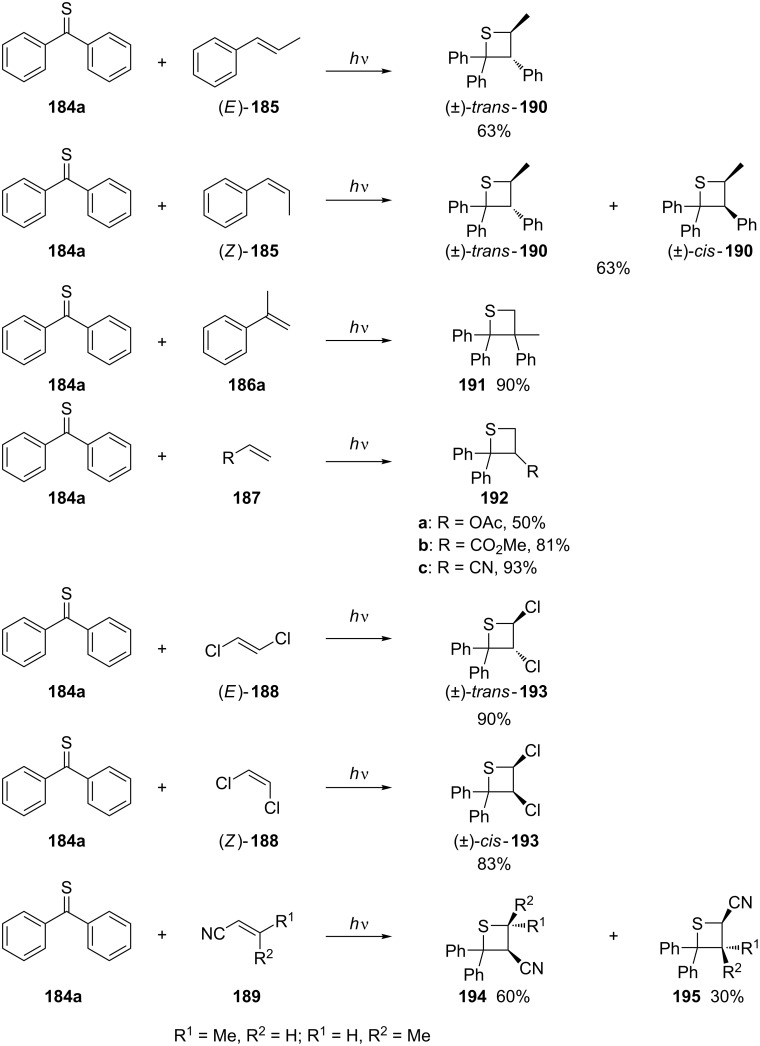
Synthesis of thietanes via the photochemical [2 + 2] cycloaddition of thiobenzophenone **184a** with various olefins.

**3.1.1 Synthesis via intermolecular photochemical [2 + 2] cycloadditions:** In 1969, the first photo-assisted [2 + 2] cycloadditions of alkenes and thiocarbonyl compounds were applied for the synthesis of thietanes. Later, this transformation was considered as thia-Paternò–Büchi reaction. The reactions of thiobenzophenone (**184a**) with both, electron-rich olefins **185, 186a**, and **187a** under irradiation with UV light at 366 nm, and electron-deficient olefins **187b**,**c**, **188**, and **189** under irradiation with either 366 nm or 589 nm UV light gave the desired thietanes **190**–**195** with retention of the olefin configuration in most cases. An exception was observed for the reaction of **184a** with (*Z*)-prop-1-enylbenzene [(*Z*)-**185**], which generated a mixture of *cis*- and *trans*-thietanes, *cis-***190** and *trans-***190**, both configuration retention and inversion products [63] (Scheme 38). However, some olefins, such as cyclohexene, oct-1-ene, vinyl ether, vinyl sulfide, etc., produced 1,4-dithiane derivatives as products through the reaction of two molecules of thiobenzophenone (**184a**) and one molecule of the olefin under irradiation with 589 nm UV light [63].

**Scheme 39:**
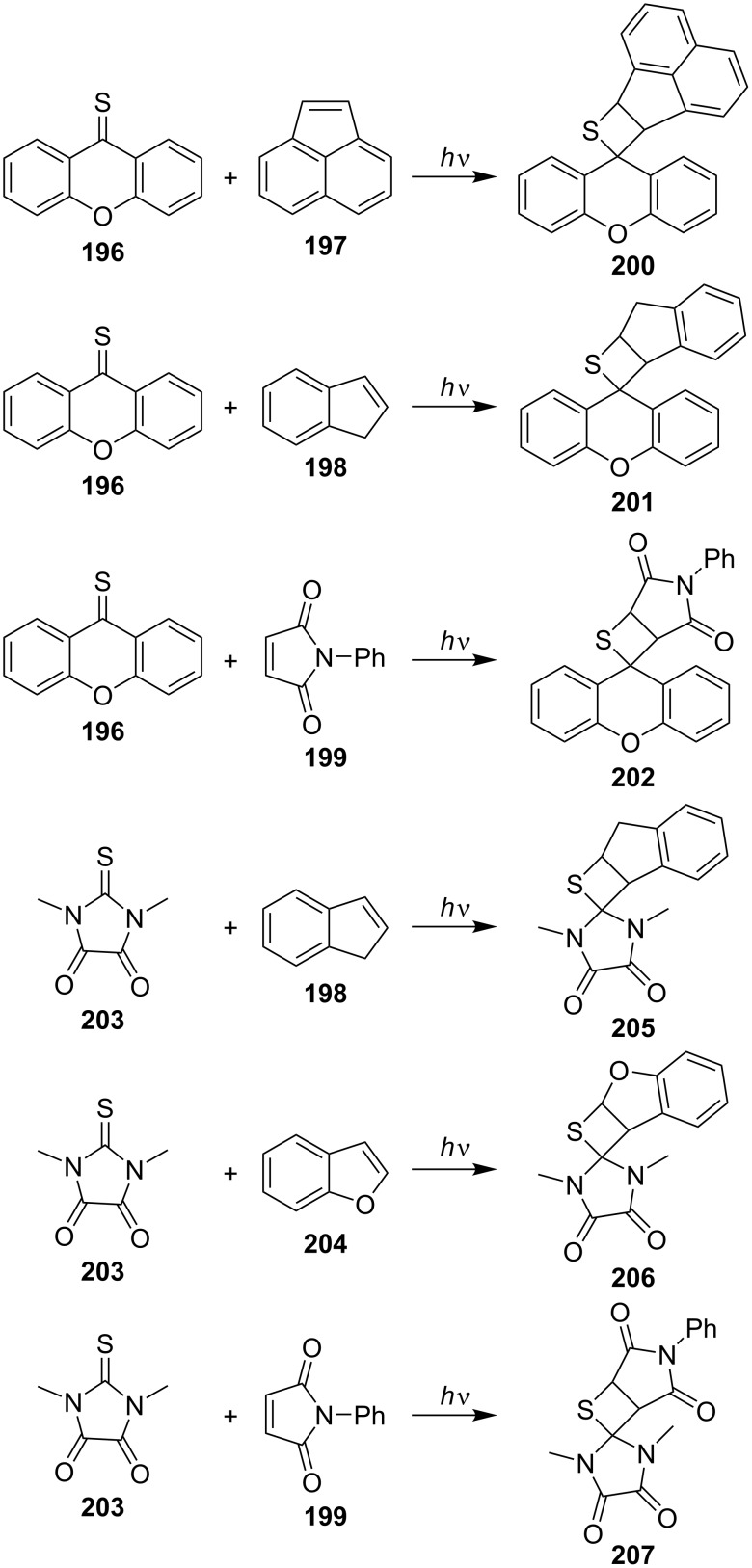
Synthesis of spirothietanes through the photo [2 + 2] cycloaddition of cyclic thiocarbonyls with olefins.

In 1978, Gotthardt and Nieberl investigated the UV light-induced [2 + 2] cycloaddition reaction of thiones with cyclic alkenes and realized the synthesis of spirothietane derivatives. Under n → π^*^ excitation using Na light, xanthione (**196**) reacted with acenaphthylene (**197**), indene (**198**), or *N*-phenylmaleimide (**199**) with the formation of the corresponding spirothietane derivatives **200**–**202** in good yields. The analogous photoreactions of 2-thioparabanate (**203**) in the presence of indene (**198**), benzo[*b*]furan (**204**), or *N*-phenylmaleimide (**199**) gave spirothietanes **205**–**207** as well [64] (Scheme 39).

**Scheme 40:**
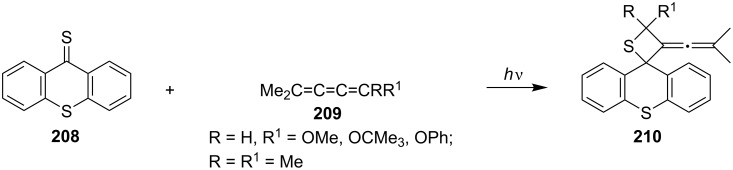
Photochemical synthesis of spirothietane-thioxanthenes **210** from thioxanthenethione (**208**) and butatrienes **209**.

The irradiation of a 0.050 mol/L solution of thioxanthenethione (**208**) in CH_2_Cl_2_ with butatrienes Me_2_C=C=C=CRR^1^ **209** through a K_2_Cr_2_O_7_ filter solution gave 70 to > 90% yields of the corresponding spirothietanes **210** [65] (Scheme 40).

The same research group also performed the reaction mechanistic studies. The reactivity of the substituted allenes towards triplet aromatic thiones was investigated. The product analysis revealed the formation of thietanes and occasionally of [4 + 2] cycloadducts (thiopyrans) generally in high overall yields. Steady-state measurements showed that electron-donating substituents present in the allenes enhanced the overall reaction rate. There was little effect of the solvent polarity on the reaction rate. The formation of thietanes involved the excited triplet thiones and the π-bond of allenes [66].

**Scheme 41:**
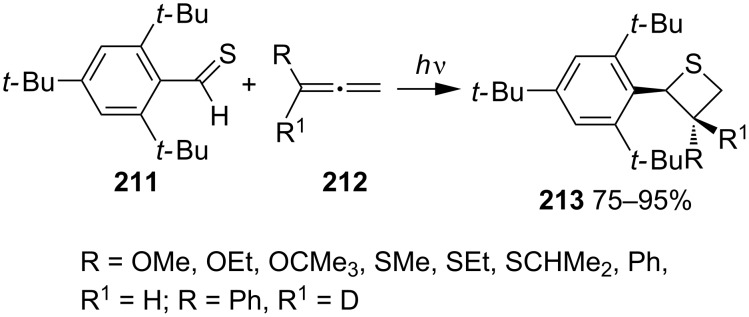
Synthesis of thietanes **213** from 2,4,6-tri(*tert*-butyl)thiobenzaldehyde (**211**) with substituted allenes.

In 1984, Bos and co-workers realized the photocycloaddition reaction of the first stable thiobenzaldehyde, 2,4,6-tri(*tert*-butyl)thiobenzaldehyde (**211**) with substituted allenes **212**. Irradiation of thiobenzaldehyde **211** with RCR^1^=C=CH_2_ (**212**) gave diastereospecific [2 + 2] cycloadducts, thietanes **213** in 75–95% yields [67] (Scheme 41).

**Scheme 42:**
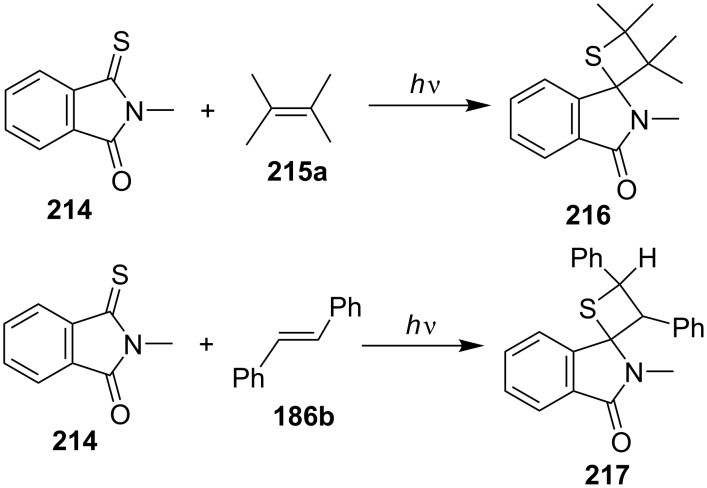
Photochemical synthesis of spirothietanes **216** and **217** from *N*-methylthiophthalimide (**214**) with olefins.

In 1984, Coyle and Rapley performed the photochemical cycloadditions of *N*-methylthiophthalimide (**214**) with 2,3-dimethylbut-2-ene (**215a**) or with stilbene (**186b**) to give spirothietanes **216** and **217**, respectively [68] (Scheme 42).

**Scheme 43:**
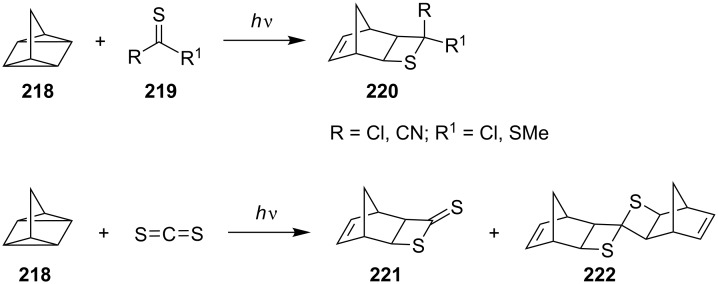
Synthesis of fused thietanes from quadricyclane with thiocarbonyl derivatives **219**.

In 1985, Jenner and Papadopoulos prepared fused thietane derivatives **220** by the photo [2 + 2] cycloaddition of quadricyclane **218** with thiocarbonyl derivatives **219**. With carbon disulfide, mono- and biscycloadducts **221** and **222** were formed depending on concentration, temperature, and pressure conditions [69] (Scheme 43).

**Scheme 44:**
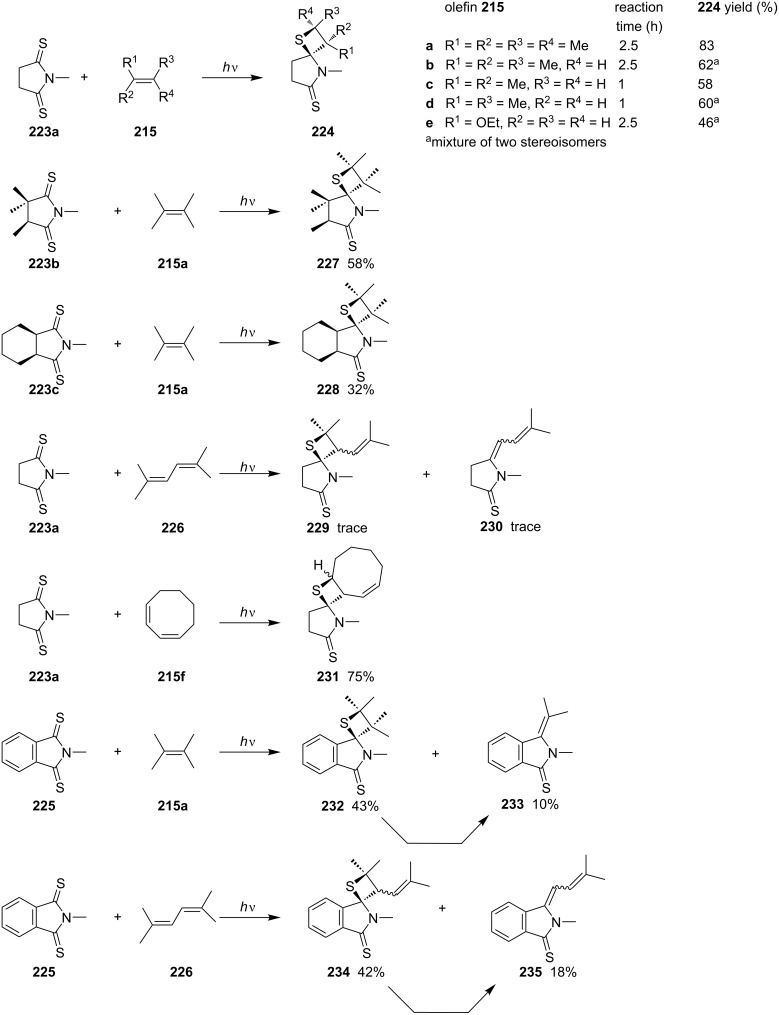
Synthesis of tricyclic thietanes via the photo [2 + 2] cycloaddition of *N*-methyldithiosuccinimides or *N*-methyldithiophthalimide with olefins.

In the same year, Kanaoka and co-workers reported the intermolecular photo [2 + 2] cycloadditions of *N*-methyldithiosuccinimides **223** and *N*-methyldithiophthalimide (**225**) with alkenes **215** and a conjugated diene **226**, generating spirothietanes **224**, **227**–**229**, **231**, **232**, and **234**. In some cases, the reverse [2 + 2] cycloaddition occurred with the loss of a molecule of thioacetone [70] (Scheme 44).

**Scheme 45:**
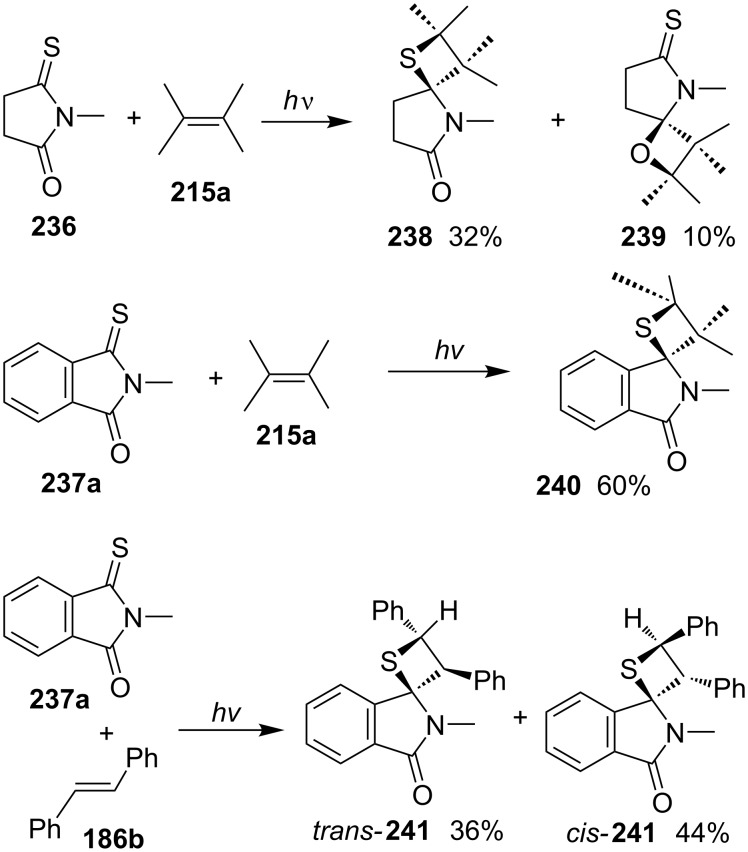
Synthesis of tricyclic thietanes via the photo [2 + 2] cycloaddition of *N*-methylthiosuccinimide/thiophthalimide with olefins.

The photoreaction of *N*-methylthiosuccinimide (**236**) with 2,3-dimethylbut-2-ene (**215a**) gave rise to a mixture of thietane and oxetane derivatives **238** and **239**, with thietane **238** as the major component. However, the reaction of the aromatic counterpart, *N*-methylmonothiophthalimide (**237a**) with olefins **215a** and **186b**, produced exclusively thietane derivatives **240** and **241** [70] (Scheme 45).

**Scheme 46:**
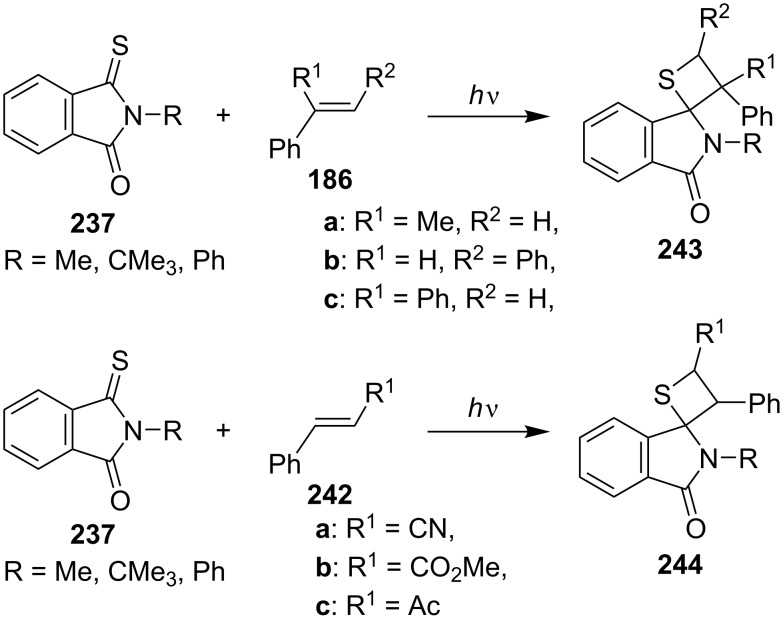
Synthesis of tricyclic thietanes via the photo [2 + 2] cycloaddition of *N*-alkylmonothiophthalimides with styrene derivatives.

The authors further investigated photoreactions of *N*-substituted monothiophthalimides **237** with styrene derivatives **186** and **242**, affording the corresponding spirothietanes **243** and **244** [71] (Scheme 46).

**Scheme 47:**
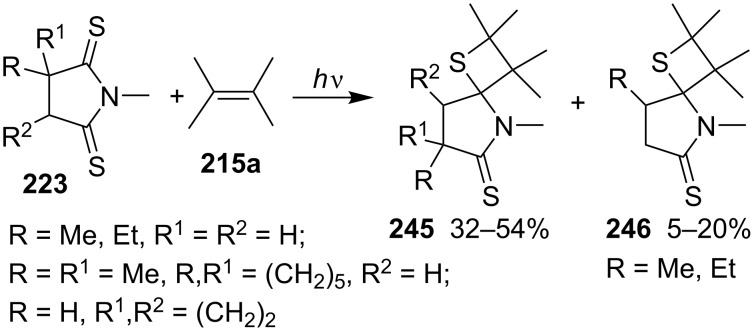
Synthesis of spirothietanes from dithiosuccinimides **223** with 2,3-dimethyl-2-butene (**215a**).

They also documented the photocycloaddition of ring-substituted cyclic dithiosuccinimides **223** with 2,3-dimethyl-2-butene (**215a**), affording a series of spirothietanes **245** [72] (Scheme 47).

In 1986, Coyle and Rapley reported that the photochemical cycloaddition reactions of *N*-methylthiophthalimide (**237a**) and *N*-methyldithiophthalimide (**225**) with alkenes worked as well [73].

**Scheme 48:**
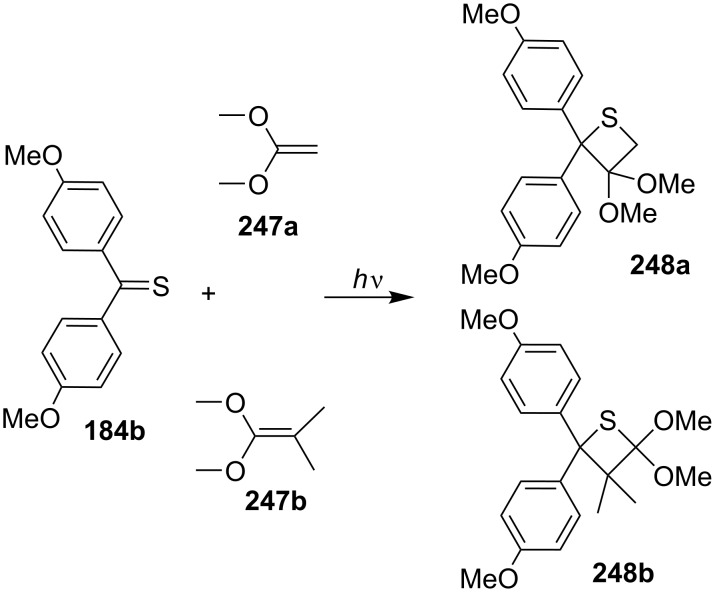
Synthesis of thietanes **248a**,**b** from diaryl thione **184b** and ketene acetals **247a**,**b**.

In 1987, Ooms and Hartmann showed the photochemical [2 + 2] cycloaddition of diaryl thione **184b** with ketene acetals **247** [74] (Scheme 48).

**Scheme 49:**
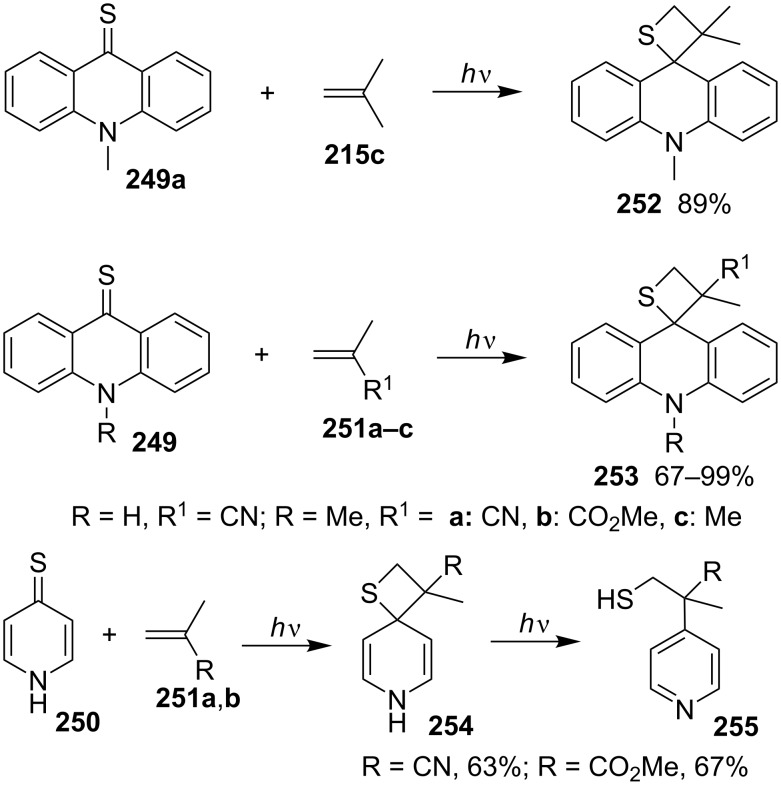
Photocycloadditions of acridine-9-thiones **249** and pyridine-4(1*H*)-thione (**250**) with 2-methylacrynitrile (**251a**) and methyl 2-methylacrylate (**251b**).

In the same year, Nishio studied the photocycloadditions of nitrogen-containing cyclic thiones **249** and **250** with 2-methylacrylnitrile (**251a**) and methyl 2-methylacrylate (**251b**), respectively, affording the corresponding spirothietanes **253** regiospecifically in 67–99% yields for acridine-9-thione **249b** and its *N*-methyl derivative **249a**. However, for pyridine-4(1*H*)-thione (**250**), the generated thietane intermediates **254** underwent ring cleavage and aromatization to give substituted pyridines **255** [75] (Scheme 49).

**Scheme 50:**
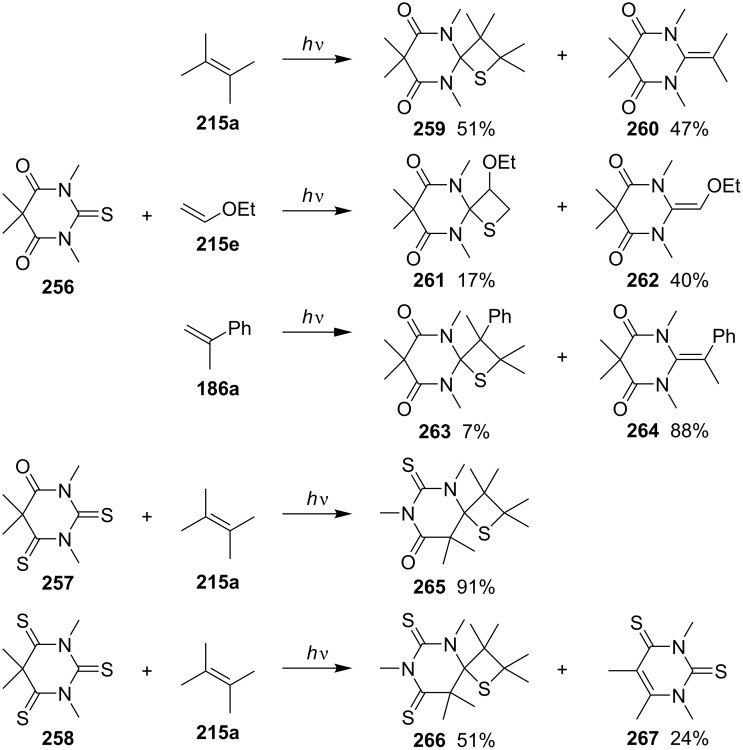
Synthesis of thietanes via the photo [2 + 2] cycloaddition of mono-, di-, and trithiobarbiturates **256**–**258** with olefins.

In 1989, Kanaoka and co-workers further studied the photo [2 + 2] cycloadditions of thiobarbiturates **256–258**, whose skeletons consisted of a combination of a thioamide and an amide or a thioamide (two-imides system), and olefins. 2-Thiobarbiturate **256** generated both, the spirothietanes **259**, **261**, and **263** and the corresponding cycloreversion products **260**, **262**, and **264**. When compound **256** was reacted with 2,3-dimethylbut-2-ene (**215a**), the spirothietane **259** was formed in slight excess. However, the cycloreversion products **262** and **264** formed preferably, in the reaction of **256** with ethyl vinyl ether (**215e**) and propen-2-ylbenzene (**186a**). Notably, the photoreaction of 2,4-dithiobarbiturate **257** and 2,3-dimethylbut-2-ene (**215a**) produced exclusively the 4-thietane derivative **265** in 91% yield. 2,4,6-Trithiobarbiturate **258** reacted with the same olefin to yield the corresponding 4-thietane derivative **266** accompanied with dithiouracil derivative **267** as byproduct [76] (Scheme 50).

**Scheme 51:**
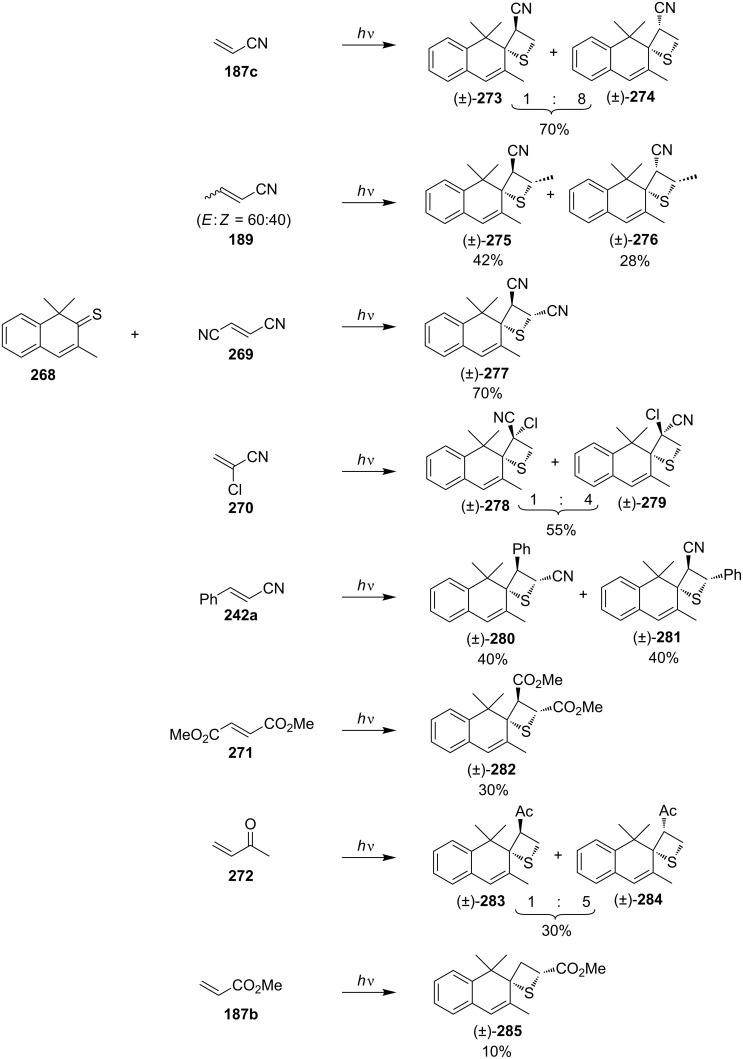
Synthesis of spirothietanes via the photo [2 + 2] cycloaddition of 1,1,3-trimethyl-2-thioxo-1,2-dihydronaphthalene (**268**) and olefins.

Rao and Ramamurthy systematically investigated the intermolecular photocycloadditions of 1,1,3-trimethyl-2-thioxo-1,2-dihydronaphthalene (**268**) with a series of electron-deficient olefins **187b**,**c**, **189**, **242a**, and **269**–**272**. The reactions afforded stereospecifically and regioselectively the 3-functionalized spirothietanes **273**–**285** as the major products. The stereospecific addition suggested either a concerted process or a pathway involving very short-lived diradicals as intermediates. To explain the regioselectivity, theoretical calculations were performed with thiochalcone and acrylonitrile as model substrates. For the frontier molecular orbital treatment, the largest coefficients in both HOMO and LUMO of thiochalcone existed on the sulfur atom, while the largest coefficients in both HOMO and LUMO of acrylonitrile were located at the β-carbon atom. These favored the overlapping between the sulfur atom and the β-carbon atom, deciding the regioselectivity [76–77] (Scheme 51).

Interestingly, the photochemical behavior of thioenones was obviously different from that of enones. The latter underwent the [2 + 2] annulation with olefins at their olefinic center to yield cyclobutane derivatives, and rarely undergo oxetane formation completely. The reaction parameters such as solvent affected the balance between the cyclobutane and oxetane formation. Whereas reactions of olefins with thioenones took place on the thiocarbonyl group to give stereospecific and regioselective thietane derivatives.

**Scheme 52:**
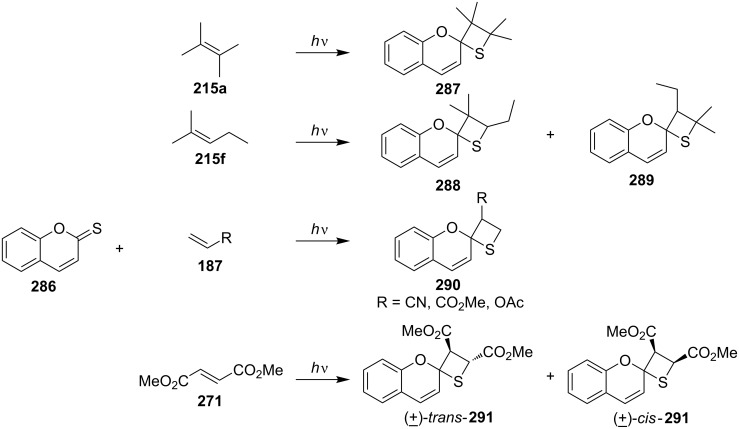
Synthesis of spirothietanes via the photo [2 + 2] cycloaddition of thiocoumarin **286** with olefins.

The same group further studied the photo [2 + 2] cycloadditions of thiocoumarin (**286**) and alkenes **187**, **215a**,**f**, and **271**, producing the corresponding spirothietane derivatives **287**–**291** [78] (Scheme 52).

**Scheme 53:**
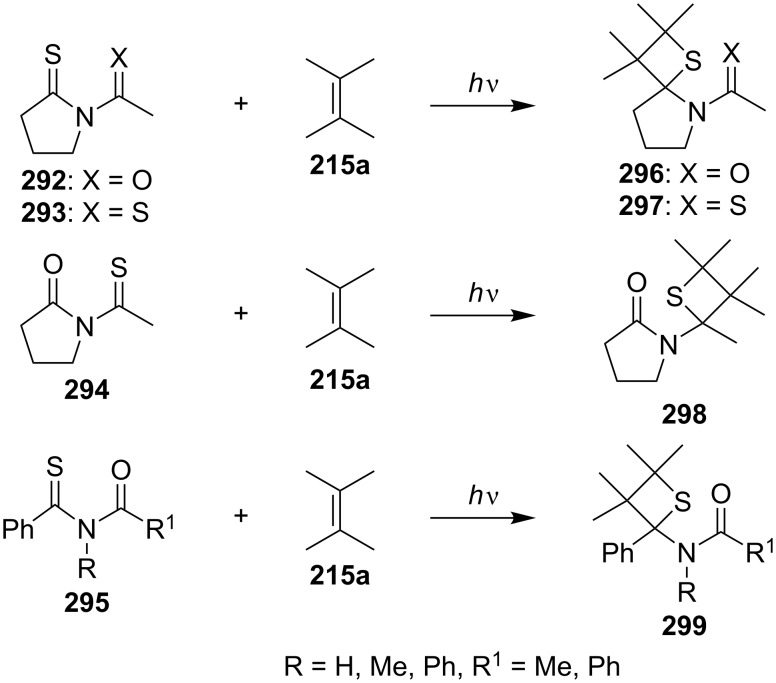
Photochemical synthesis of thietanes **296**–**299** from semicyclic and acyclic thioimides **292**–**295** and 2,3-dimethylbut-2-ene (**215a**).

In 1988, Kanaoka et al. studied the photochemistry of semicyclic and acyclic thioimides **292**–**294** and **295** with 2,3-dimethylbut-2-ene (**215a**) afforded the corresponding thietanes **296–299.** However, the products were obtained together with pyrrolidinone, thiopyrrolidinone, or thiobenzamides as byproducts. The latter were generated in the competition between Paternò–Büchi-type and Norrish-type I reactions [79] (Scheme 53).

**Scheme 54:**
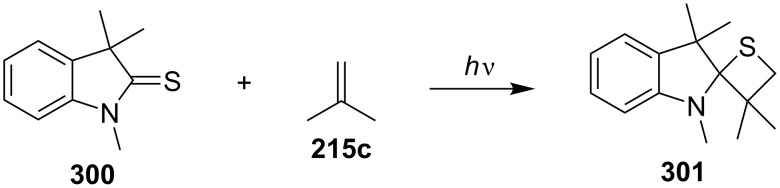
Photochemical synthesis of spirothietane **301** from 1,3,3-trimethylindoline-2-thione (**300**) and isobutene (**215c**).

In the same year, Nishio and co-workers, investigated the photochemical [2 + 2] cycloadditions of indoline-2-thiones with cyanoalkenes. Only 2-alkylideneindolines were obtained via a ring cleavage of the thietanes, that had formed in the [2 + 2] photocycloaddition of the thiocarbonyl moiety and the olefin. However, the reaction of 1,3,3-trimethylindoline-2-thione (**300**) and isobutene (**215c**) afforded the corresponding spiroindoline-thietane derivative **301** [80] (Scheme 54).

**Scheme 55:**
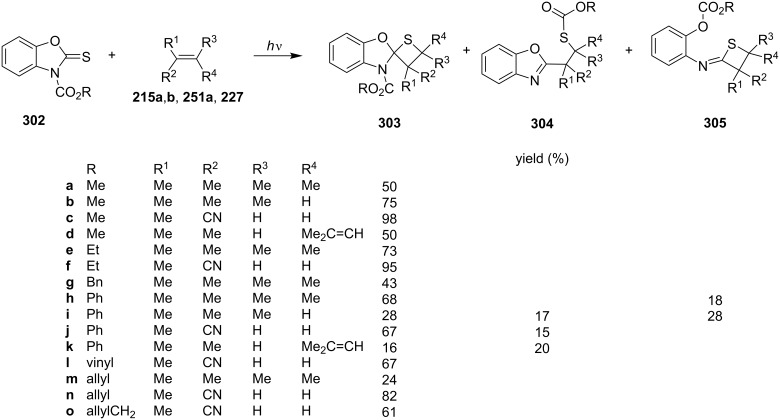
Synthesis of spirobenzoxazolethietanes **303** via the photo [2 + 2] cycloaddition of alkyl and aryl 2-thioxo-3*H*-benzoxazole-3-carboxylates **302** and various alkenes.

They further investigated the photochemical [2 + 2] cycloadditions of alkyl and aryl 2-thioxo-3*H*-benzoxazole-3-carboxylates **302** and alkenes **215a**,**b**, **251a**, and **227**, affording the corresponding spirobenzoxazole-thietane derivatives **303** [81–84] (Scheme 55).

**Scheme 56:**
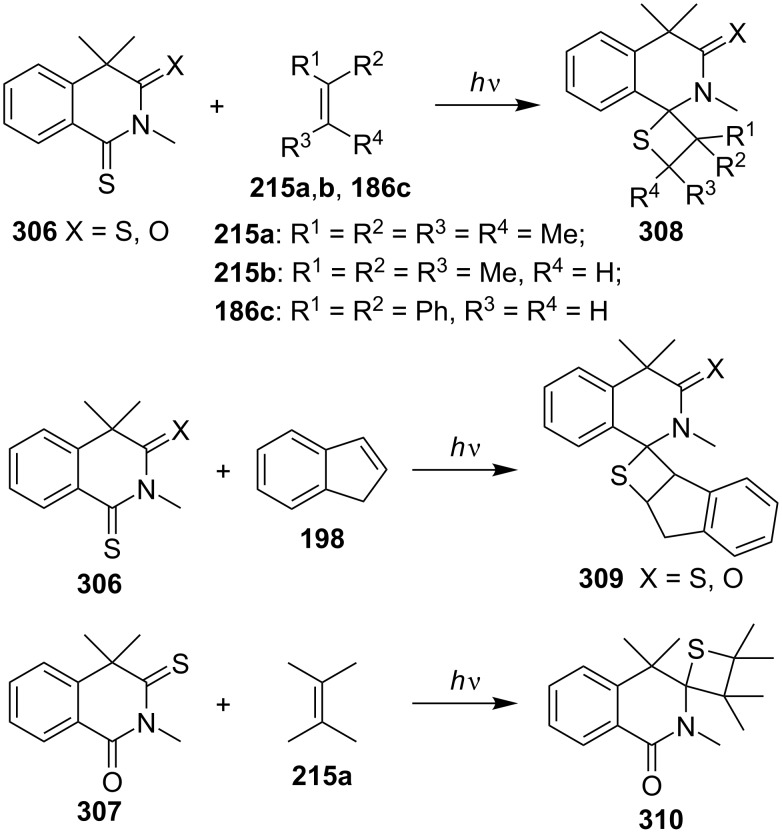
Synthesis of spirothietanes from tetrahydrothioxoisoquinolines **306** and **307** with olefins.

Upon the irradiation of tetrahydrotrimethyldithioxo and [3-oxo-1-thioxo or 1-oxo-3-thioxo]isoquinolines **306** and **307** with olefins **215a**,**b**, and **186c** or indene (**198**), the regioselective [2 + 2] cycloaddition occurred to give oxo- or thiooxospiro[isoquinoline-1,2'(or 3,2')-thietane] derivatives **208–310**. In some cases, the products were accompanied with the related alkylidenetetrahydrotrimethylthioxoisoquinolines as the byproducts [85] (Scheme 56).

Similar intramolecular photoreactions of *N*-alkenylthiohomophthalimides were attempted as well, affording the tetracyclic thietane-fused isoquinoline derivatives regioselectively [86].

**Scheme 57:**
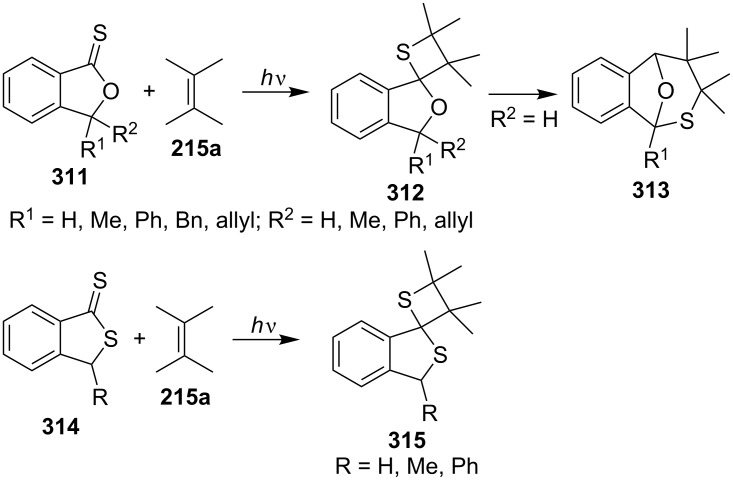
Synthesis of spirothietanes from 1,3-dihydroisobenzofuran-1-thiones **311** and benzothiophene-1-thiones **314** with 2,3-dimethylbut-2-ene (**215a**).

The reactions of isobenzofuran-1-thiones **311** and 2-benzothiophene-1-thiones **314** with 2,3-dimethylbut-2-ene (**215a**) gave the corresponding spirothietanes **312** and **315** under photo irradiation. The spirothietanes **312** derived from 3-unsubstituted or 3-monosubstituted 1,3-dihydroisobenzofuran-1-thiones **311** were less stable and underwent a thermal rearrangement to generate tricyclic isobenzofurans **313** through the ring-cleavage of the thietanes. It was assumed that the rearrangement was assisted through participation of the oxygen lone-pair electrons [17] (Scheme 57).

**Scheme 58:**
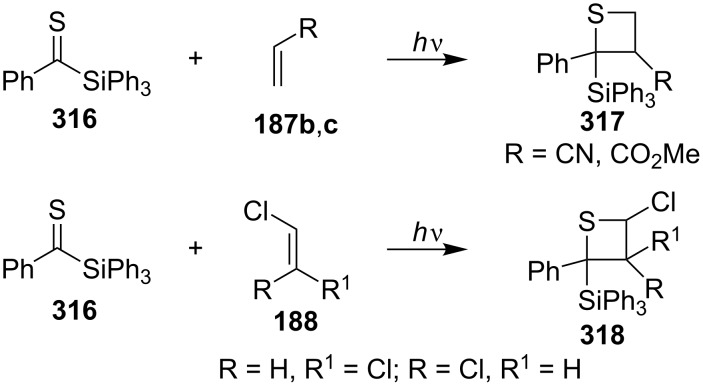
Synthesis of 2-triphenylsilylthietanes from phenyl triphenylsilyl thioketone (**316**) with electron-poor olefins.

The silicon-containing phenyl triphenylsilyl thioketone (**316**) reacted with electron-poor olefins, such as acrylonitrile (**187b**), methyl acrylate (**187c**), and *cis*- and *trans*-1,2-dichloroethenes **188**, under photochemical conditions, giving 2-silylthietanes **317** and **318** in a regio- and highly stereoselective manner. However, silyl thietanes without any regio- or stereocontrol were obtained when **316** was reacted with electron-rich olefins, as for example, vinyl ethers [87] (Scheme 58).

**Scheme 59:**
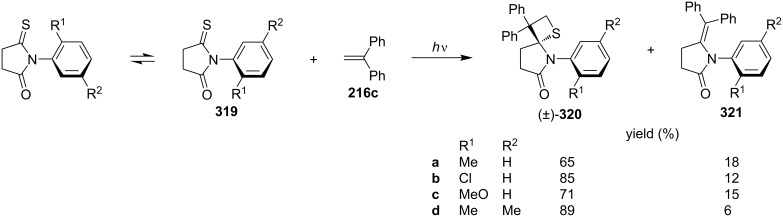
Diastereoselective synthesis of spiropyrrolidinonethietanes **320** via the photo [2 + 2] cycloaddition of *N*-arylthiosuccinimides **319** and 1,1-diphenylethene (**216c**).

In 2003, Sakamoto and co-coworkers investigated the intermolecular diastereoselective photo [2 + 2] cycloaddition of axially chiral monothiosuccinimides **319** which could enantiomerize into both (*R*) and (*S*)-isomers, and 1,1-diphenylethene (**216c**) under UV irradiation. As the products spirothietane-pyrrolidinones **320** were obtained in 65–89% yield. The diastereoselectivity was controlled by the steric effect of the *ortho*-substituents on the phenyl ring [88] (Scheme 59).

**Scheme 60:**
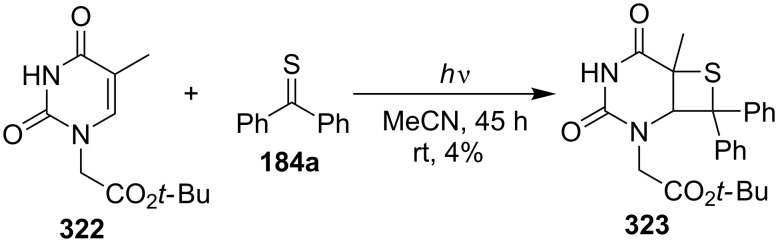
Synthesis of bicyclic thietane **323** via the photo [2 + 2] cycloaddition of 2,4-dioxo-3,4-dihydropyrimidine **322** and thiobenzophenone (**184a**).

The intermolecular photochemical [2 + 2] cycloaddition of *tert*-butyl 2-(5-methyl-2,4-dioxo-3,4-dihydropyrimidin-1(2*H*)-yl)acetate (**322**) and thiobenzophenone (**184a**) was applied to prepare thietane **323** as a model compound for photolyses in a comparative flavin-induced cleavage study of oxetanes and thietanes [89] (Scheme 60).

**Scheme 61:**
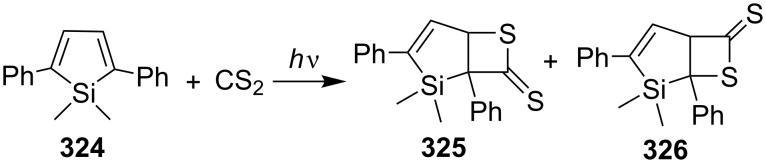
Photo-induced synthesis of fused thietane-2-thiones **325** and **326** from silacyclopentadiene **324** and carbon disulfide.

2,5-Diphenylsilacyclopentadiene (**324**) underwent a photo-induced [2 + 2] cycloaddition with CS_2_ to afford two regioisomeric fused thietane-2-thiones **325** and **326**. The electron transfer from the singlet-excited state of silacyclopentadiene to CS_2_ was shown to play an important role in the cycloaddition [90] (Scheme 61).

**Scheme 62:**
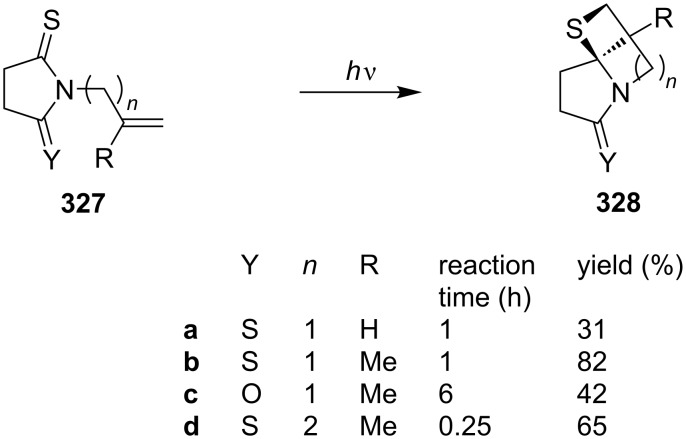
Synthesis of highly strained tricyclic thietanes **328** via the intramolecular photo [2 + 2] cycloaddition of *N*-allyl/but-3-enylthiosuccinimides **327**.

**3.1.2 Synthesis via intramolecular photochemical [2+2] cycloadditions:** In 1985, Machida’s group reported the intramolecular photo-assisted [2 + 2] cycloadditions of *N*-allylthiosuccinimides **327** applying 1 kW high-pressure mercury lamp irradiation under a nitrogen atmosphere, giving the highly strained tricyclic thietanes **328** [91] (Scheme 62).

**Scheme 63:**
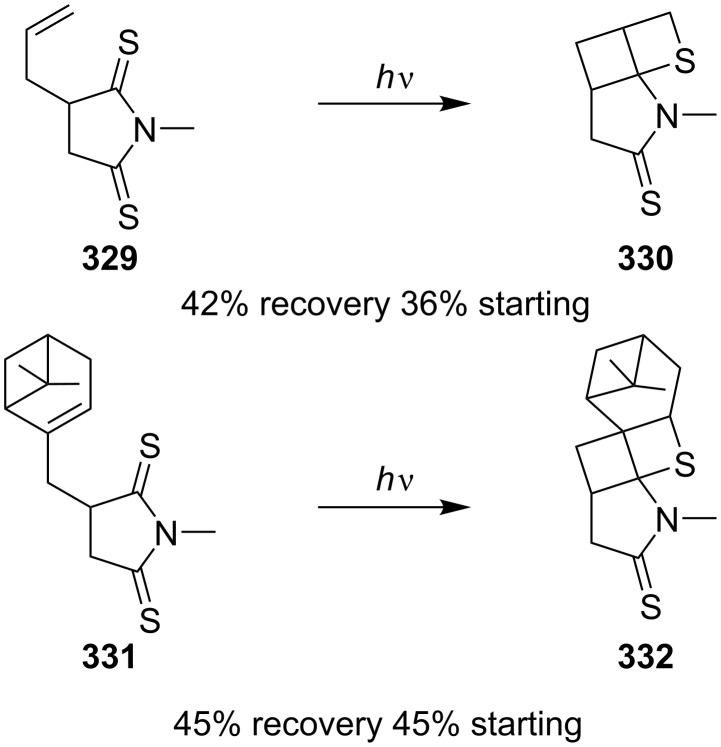
Synthesis of tri- and pentacyclic thietanes **330** and **332**, respectively, through the intramolecular photo [2 + 2] cycloaddition of allyldithiosuccinimides **329** and **331**.

One year later, the same group reported the intramolecular photo [2 + 2] cycloadditions of 2-allyl-*N*-methyldithiosuccinimide (**329**) with the same irradiation source at room temperature for 1 h, generating another highly strained tricyclic thietane **330**. 2-((6,6-Dimethylbicyclo[3.1.1]hept-2-en-2-yl)methyl)-*N*-methyldithiosuccinimide (**331**) gave rise to the pentacyclic thietane derivative **332** under the same conditions [92] (Scheme 63).

**Scheme 64:**
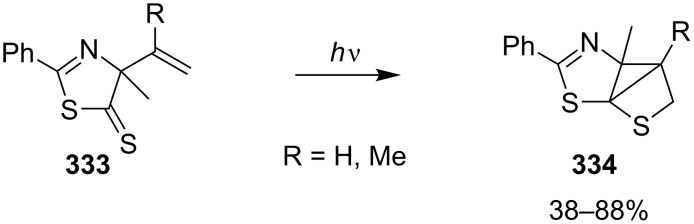
Synthesis of tricyclic thietanes **334** via the intramolecular photo [2 + 2] cycloaddition of *N*-vinylthiazolethiones **333**.

In 1987, Wipf and Heimgartner realized the photochemical intramolecular [2 + 2] cycloaddition of vinylthiazolethiones **333** to give tricyclic thietane derivatives **334** in 38–88% yields [93] (Scheme 64).

**Scheme 65:**
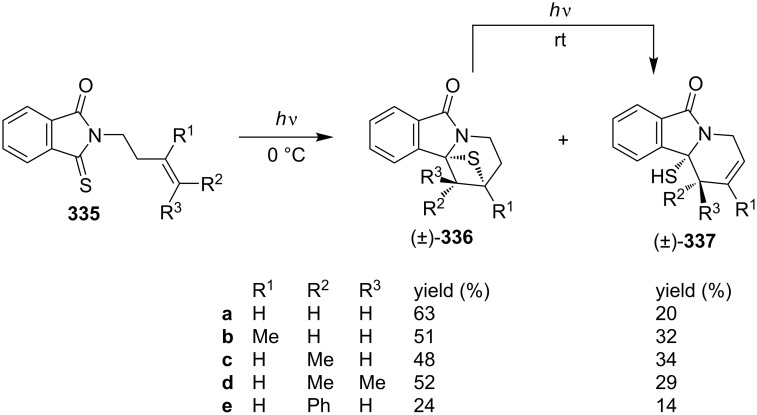
Synthesis of tricyclic thietanes **336** via the intramolecular photo [2 + 2] cycloaddition of *N*-but-3-enylthiophthalimides **335** and photochemical conversion to pyridoisoindolones **337**.

In 1992, Oda’s group found that, under photo irradiation conditions *N*-but-3-enylthiophthalimides **335** underwent an intramolecular photo-assisted [2 + 2] cycloaddition first giving tricyclic thietanes **336**, which further photochemically converted into pyridoisoindolones **337** [94] (Scheme 65).

**Scheme 66:**
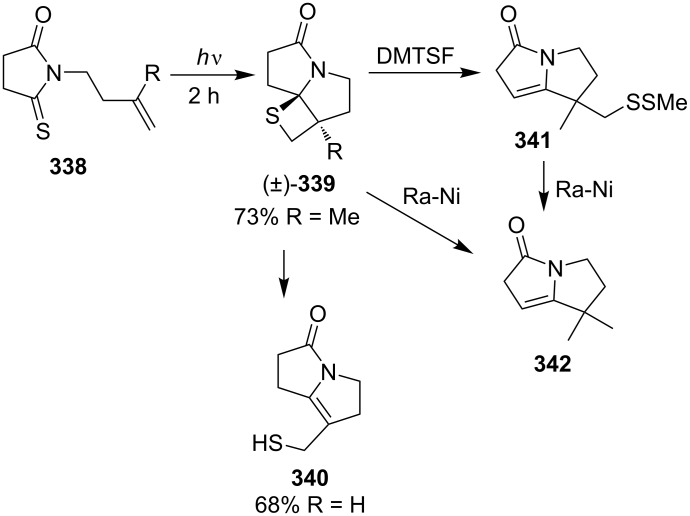
Synthesis of tricyclic thietanes via the intramolecular photo [2 + 2] cycloaddition of *N*-but-3-enylthiosuccinimides **338**.

To synthesize various pyrrolizidine alkaloids, Padwa’s group used the intramolecular photocycloaddition of *N*-but-3-enyl-5-thiopyrrolidin-2-ones **338**. The intramolecular photo [2 + 2] cycloadditions first generated the tricyclic thietanes **339**, which further underwent a ring-opening reaction to afford pyrrolizinones **342**. In case of *N*-but-3-enyl-5-thiopyrrolidin-2-one (**338a**, R = H) the reaction afforded the product 7-mercaptomethyl-1,2,5,6-tetrahydropyrrolizin-3-one (**340**) directly in 68% yield under photo irradiation. However, *N*-(3-methylbut-3-enyl)-5-thiopyrrolidin-2-one (**338b**, R = Me) initially generated a tricyclic fused thietane derivative (**339b**, R = Me), which gave rise to 2,5,6,7-tetrahydropyrrolizin-3-one **341** upon the treatment with dimethyl(methylthio)sulfonium tetrafluoroborate (DMTSF), or hexahydropyrrolizin-3-one **342** in the presence of Ra-Ni in ethanol [18,95] (Scheme 66).

**Scheme 67:**
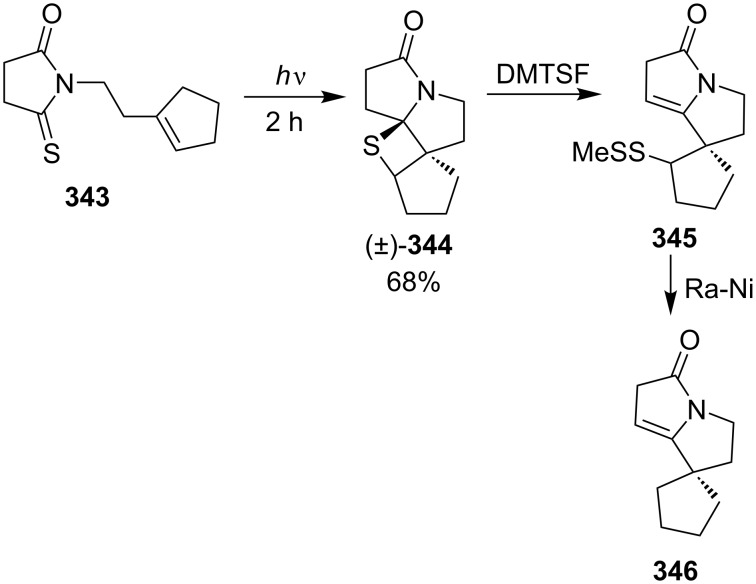
Synthesis of tetracyclic thietane **344** through the intramolecular photo [2 + 2] cycloaddition of *N*-[2-(cyclopenten-1-yl)ethyl]thiosuccinimide **343**.

*N*-1-(Cyclopent-1-enyl)ethyl-5-thiopyrrolidin-2-one (**343**) gave a tetracyclic thietane derivative **344**, which further afforded a spirocyclopentane tetrahydropyrrolizin-3-one **346** under the similar treatments [95] (Scheme 67).

**Scheme 68:**
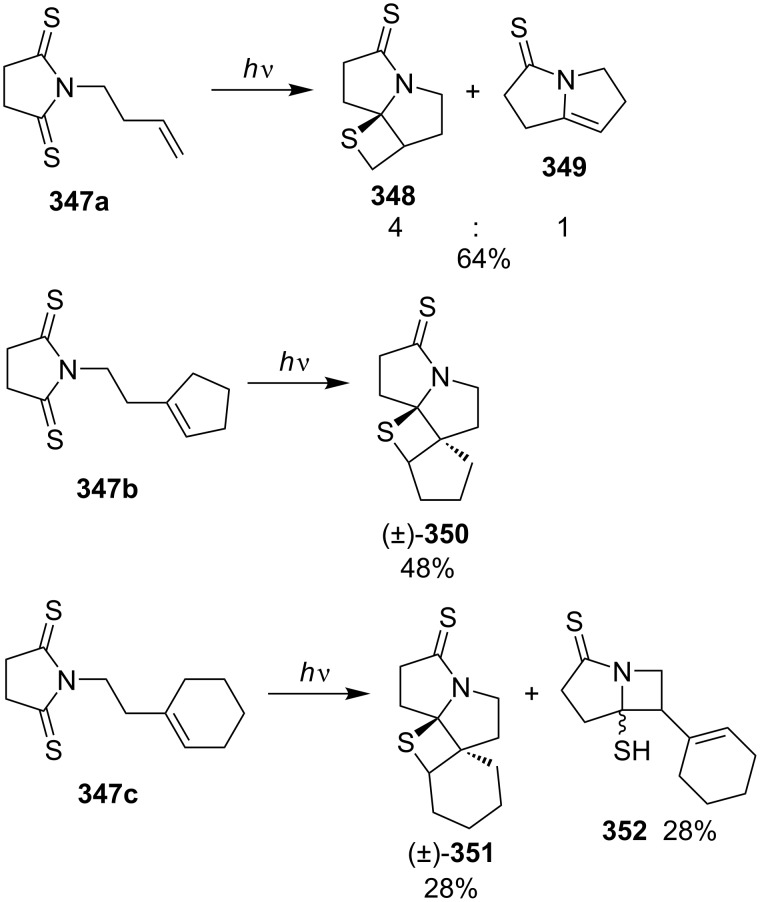
Synthesis of tri- and tetracyclic thietanes **348**, **350**, and **351**, through the intramolecular photo [2 + 2] cycloaddition of *N*-but-3-enyldithiosuccinimides **347a**–**c**.

Similarly, linear and cyclic 3-but-3-enylpyrrolidine-2,5-dithiones **347** gave tricyclic and tetracyclic fused thietane derivatives **348**, **350**, and **351** under photo irradiation [95] (Scheme 68).

**Scheme 69:**
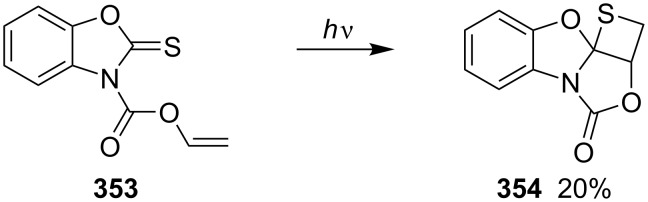
Synthesis of tetracyclic fused thietane **354** via the photo [2 + 2] cycloaddition of vinyl 2-thioxo-3*H*-benzoxazole-3-carboxylate (**353**).

Nishio and co-workers investigated the photochemical [2 + 2] cycloaddition of vinyl 2-thioxo-3*H*-benzoxazole-3-carboxylate (**353**), affording the corresponding tetracyclic fused benzoxazolethietane derivative **354** in 20% yield [83] (Scheme 69).

**Scheme 70:**
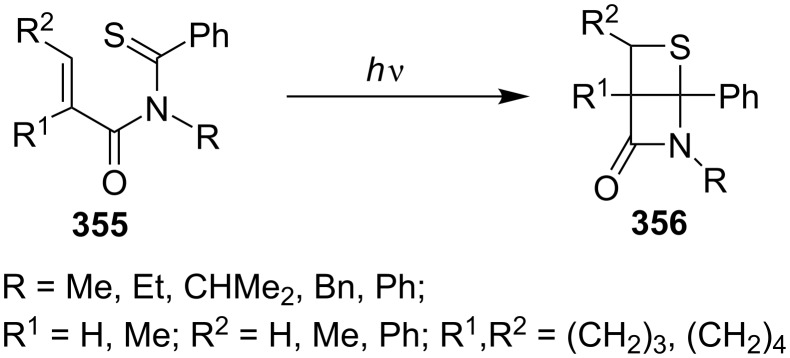
Synthesis of highly rigid thietane-fused β-lactams via the intramolecular photo [2 + 2] cycloaddition of monothioimides **355**.

In 1991, Sakomto and co-workers started on the synthesis of highly rigid thietane-fused β-lactams. They prepared various derivatives **356** in high yields via the photochemical cycloaddition reactions of *N*-(α,β-disubstituted alkyl-2-enoyl)thiobenzamides **355**. Some thioamides **355**, (i.e., R = CHMe_2_), formed R^2^CH=CR^1^CONHCMe_2_CSPh via a β-H abstraction of the thiocarbonyl group. Substituents at the α-position to the alk-2-enoyl moiety led to a preference for the [2 + 2] cyclization over the β-H abstraction. The reaction was shown to proceed via an *n*–π* triplet-excited state [96] (Scheme 70).

**Scheme 71:**
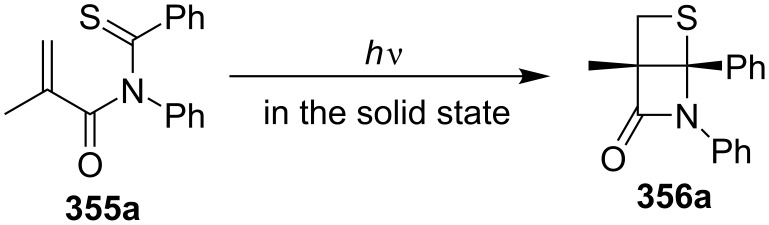
Asymmetric synthesis of a highly rigid thietane-fused β-lactam **356a** via the intramolecular photo [2 + 2] cycloaddition of monothioimide **355a**.

In 1993, the same group first attempted to prepare a chiral thietane-fused β-lactam **356a** from an achiral monothioimide **355a** using a chiral crystal environment through a topochemically controlled intramolecular photochemical [2 + 2] cycloaddition. The reaction afforded the product in 70% yield with 40% ee at −45 °C and in 75% yield with 10% ee at 0 °C, respectively [97] (Scheme 71).

**Scheme 72:**
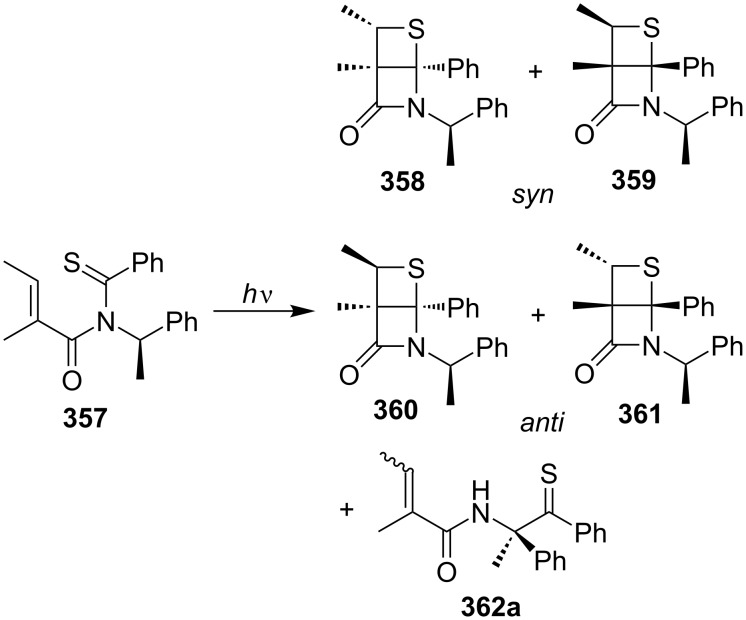
Diastereoselective synthesis of the thietane-fused β-lactams via the intramolecular photo [2 + 2] cycloaddition of the chiral monothioimide **357**.

One year later, they studied the diastereoselective synthesis of highly rigid thietane-fused β-lactams **358–361** from a chiral monothioimide **357**. The photochemical [2 + 2] cycloaddition reaction was performed both in benzene solution and in the solid state, affording 78% yield with a ratio of *syn*/*trans* 8.7:1 and 61% de for *syn*-isomers at 15 °C in crystals, while no diastereoselectivity could be observed in benzene solution [16] (Scheme 72).

**Scheme 73:**
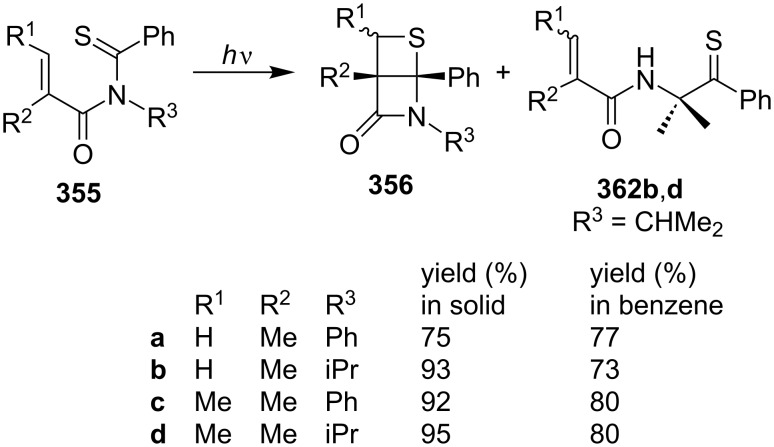
Asymmetric synthesis of thietane-fused β-lactams **356** via the intramolecular photo [2 + 2] cycloaddition of monothioimides **355**.

In 2001, they performed the absolute asymmetric synthesis of highly rigid thietane-fused β-lactams **356** from achiral monothioimides **355** using a chiral crystal environment through a topochemically controlled intramolecular photochemical [2 + 2] cycloaddition in a benzene solution. Only the 2-methylacrylamide derivative **355a** afforded the desired product **356a** in 70% yield with 40% ee at −45 °C and 75% yield with 10% ee at 0 °C, respectively, in the solid state [98] (Scheme 73).

Compared with cyclic thioetherification reactions, the photochemical cycloadditions of thiocarbonyl compounds and olefins are highly suitable for the preparation of multiple substituted thietanes, including fused and spirothietanes.

#### 3.2 Synthesis via the formal thermal [2 + 2] cycloadditions involving hexafluorothioacetone

**Scheme 74:**
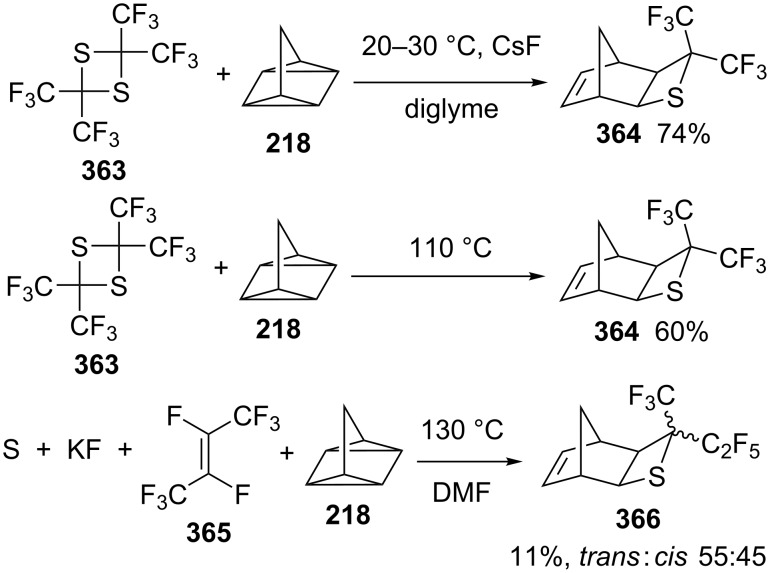
Synthesis of the bridged bis(trifluoromethyl)thietane from 2,2,4,4-tetrakis(trifluoromethyl)-1,3-dithietane (**363**)and quadricyclane (**218**).

The formal thermal [2 + 2] cycloadditions have also been applied in the synthesis of bis(trifluoromethyl)thietanes from 2,2,4,4-tetrakis(trifluoromethyl)-1,3-dithietane-generated bis(trifluoromethyl)thioacetone with various olefins in nucleophilic solvents DMF or DMSO. Previously, the reaction of 2,2,4,4-tetrakis(trifluoromethyl)-1,3-dithietane (**363**) and quadricyclane (**218**) was carried out in diglyme in the presence of CsF as catalyst, affording the thietane **364** in 74% yield. However, a 60% yield of the thietane **364** was obtained without the catalyst and solvent. The reaction of sulfur, KF, perfluorobut-2-ene (**365**) and quadricyclane (**218**) in DMF at 130 °C generated 4-trifluoromethyl-4-pentafluoroethyl-3-thiatricyclo[4.2.1.0^2,5^]non-7-ene (**366**) in 11% yield with a *trans*:*cis* ratio of 55:45 [99] (Scheme 74).

**Scheme 75:**
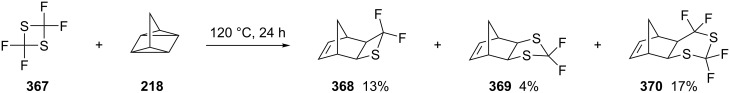
Synthesis of the bridged-difluorothietane **368** from 2,2,4,4-tetrafluoro-1,3-dithietane (**367**) and quadricyclane (**218**).

The reaction of 2,2,4,4-tetrafluoro-1,3-dithietane (**367**) and quadricyclane (**218**) generated the difluoro-bridged thietane **368** in 13% yield and two other byproducts **369** and **370** [99] (Scheme 75).

**Scheme 76:**
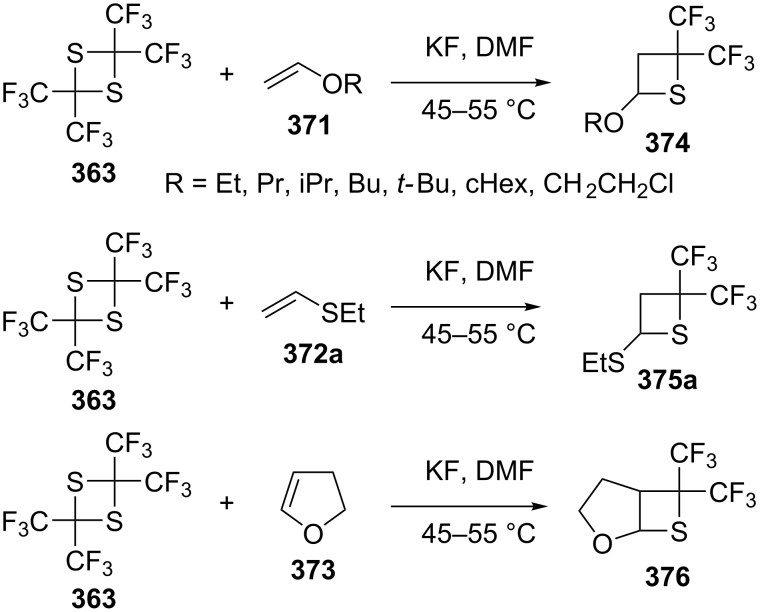
Synthesis of bis(trifluoromethyl)thietanes from 2,2,4,4-tetrakis(trifluoromethyl)-1,3-dithietane (**363**) and electron-rich olefins.

The reaction of 2,2,4,4-tetrakis(trifluoromethyl)-1,3-dithietane (**363**) with electron-rich olefins **371** and **372a** gave the corresponding thietanes **374** and **375a**. On the other hand, reacting 2,2,4,4-tetrakis(trifluoromethyl)-1,3-dithietane (**363**) with 2,3-dihydrofuran (**373**) gave the corresponding fused thietane 6,6-bis(trifluoromethyl)-2-oxa-7-thiabicyclo[3.2.0]heptane (**376**) [19] (Scheme 76).

**Scheme 77:**
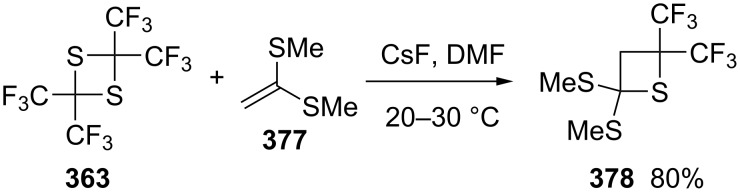
Synthesis of 2,2-dimethylthio-4,4-di(trifluoromethyl)thietane (**378**) from 2,2,4,4-tetrakis(trifluoromethyl)-1,3-dithietane (**363**) and 1,1-dimethylthioethene (**377**).

The reaction of 2,2,4,4-tetrafluoro-1,3-dithietane (**363**) with 1,1-dimethylthioethene (**377**) generated 2,2-dimethylthio-4,4-di(trifluoromethyl)thietane (**378**) in 80% yield [100] (Scheme 77).

**Scheme 78:**
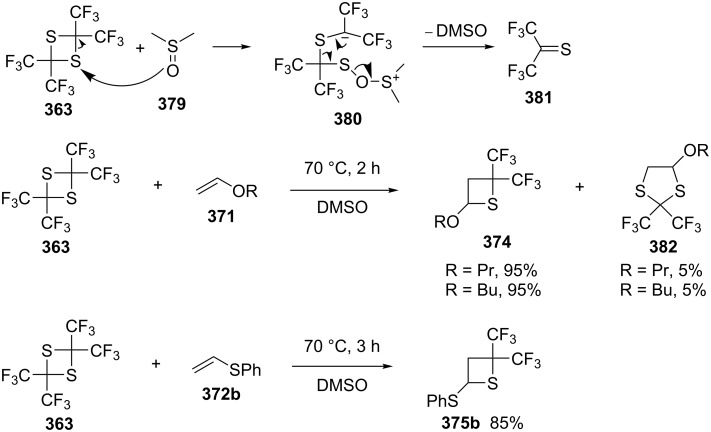
Formation of bis(trifluoromethyl)thioacetone (**381**) through nucleophilic attack of dithietane **363** by DMSO (**379**) and synthesis of 2,2-bis(trifluoromethyl)thietanes **375** and **376b** from 2,2,4,4-tetrakis(trifluoromethyl)-1,3-dithietane (**363**) and olefins in DMSO.

A recent mechanistic investigation revealed that the CsF catalyst was not required. The solvent, such as DMSO (**379**), nucleophilically attacked the 1,3-dithietane **363**, resulting in ring opening and further formation of bis(trifluoromethyl)thioacetone (**381**). The latter reacted with olefins **371** to afford thietanes **374**. The reaction of 2,2,4,4-tetrakis(trifluoromethyl)-1,3-dithietane (**363**) with alkyl vinyl ethers **371** or phenyl vinyl sulfide (**372b**) in DMSO at 70 °C afforded the corresponding 2,2-bis(trifluoromethyl)-3-alkoxy/phenylthiothietanes **374** and **375b**, respectively, with 1,3-dithiolanes **382** as byproducts [101] (Scheme 78).

**Scheme 79:**
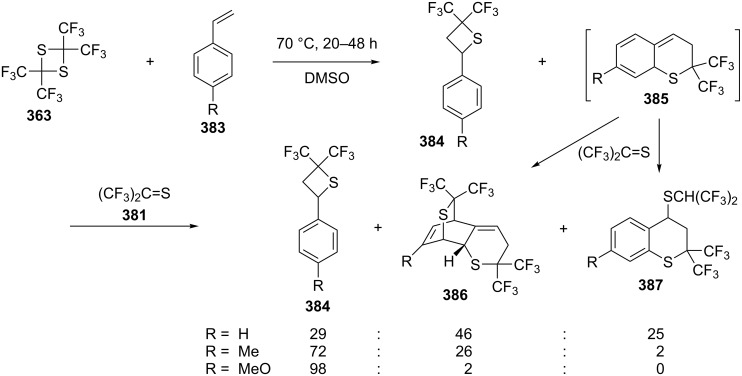
Synthesis of 2,2-bis(trifluoromethyl)thietanes from 2,2,4,4-tetrakis(trifluoromethyl)-1,3-dithietane (**363**) and styrenes **383** in DMSO.

The reactions of 2,2,4,4-tetrakis(trifluoromethyl)-1,3-dithietane (**363**) and styrenes **383** produced the [2 + 2] adducts 4-aryl-2,2-bis(trifluoromethyl)thietanes **384** and Diels–Alder adducts **385**, which further reacted with another molecule of bis(trifluoromethyl)thioketone (**381**) to yield the double Diels–Alder adducts **385** and thiochromane derivatives **386**, respectively, through another Diels–Alder reaction and an ene reaction [101] (Scheme 79).

**Scheme 80:**
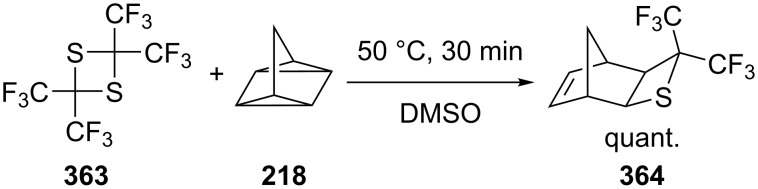
Synthesis of the bridged bis(trifluoromethyl)thietane **364** from of 2,2,4,4-tetrakis(trifluoromethyl)-1,3-dithietane (**363**) and quadricyclane (**218**) in DMSO.

The reaction of 2,2,4,4-tetrakis(trifluoromethyl)-1,3-dithietane (**363**) and quadricyclane (**218**) gave the bridged-thietane derivative **364** quantitatively in DMSO at 50 °C within 30 min [101] (Scheme 80).

#### 3.3 Synthesis via formal [2 + 2] cycloadditions

**Scheme 81:**
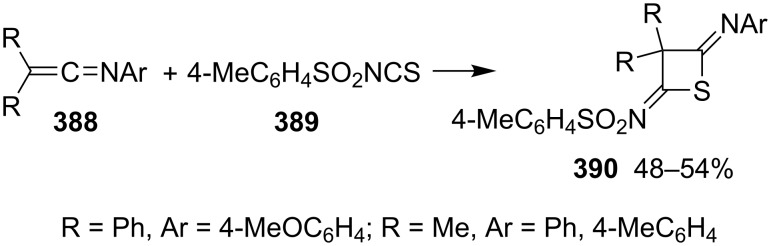
Synthesis of 2,4-diiminothietanes **390** from alkenimines and 4-methylbenzenesulfonyl isothiocyanate (**389**).

The [2 + 2] cycloaddition of alkenimines (R_2_C=C=NAr, **388**) and 4-methylbenzenesulfonyl isothiocyanate (4-MeC_6_H_4_SO_2_NCS, **389**) gave 2,4-diiminothietanes **390** in 48–54% yields. This is a general method to prepare 2,4-diiminothietane derivatives [102] (Scheme 81).

**Scheme 82:**
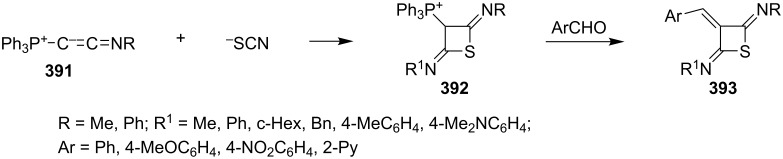
Synthesis of arylidene 2,4-diiminothietanes **393** starting from phosphonium ylides **391** and isothiocyanates.

Phosphonium ylides, Ph_3_P^+^-C^−^=C=NR (**391**) reacted with isothiocyanate in a [2 + 2] cycloaddition to form the four-membered ring phosphonium ylides **392**, which further reacted with aromatic aldehydes to afford the corresponding arylidene-2,4-diiminothietanes **393** [103] (Scheme 82).

**Scheme 83:**
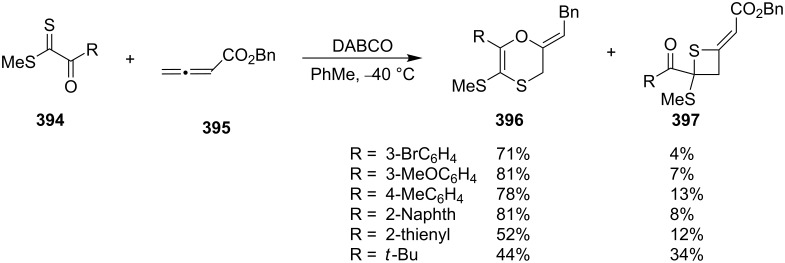
Synthesis of thietane-2-ylideneacetates **397** through a DABCO-catalyzed formal [2 + 2] cycloaddition of benzyl allenoate (**395**) and dithioesters **394**.

Thietan-2-ylideneacetates **397** were synthesized through amine-catalyzed formal [2 + 2] cycloadditions. The DABCO-catalyzed tunable formal [4 + 2] and [2 + 2] cycloadditions of benzyl allenoate (**395**) and methyl 2-oxoalkanedithioates **394** generated the 5-(methylthio)-2-phenylethylidene-2,3-dihydro-1,4-oxathiines **396** with benzyl thietane-2-ylideneacetates **397** as byproducts [104] (Scheme 83).

### 4. Synthesis via the ring expansions and contractions

#### 4.1 Synthesis via ring expansion

The ring expansions of thiiranes are alternative ways to prepare thietane derivatives. The transformations included the nucleophilic ring expansion of (1-haloalkyl)thiiranes with various nucleophiles, nucleophilic ring expansion of thiiranes with sulfur ylides, and the electrophilic ring expansion of thiiranes with carbenes generated from sulfur ylides under the catalysis of transition-metal catalysts.

**Scheme 84:**
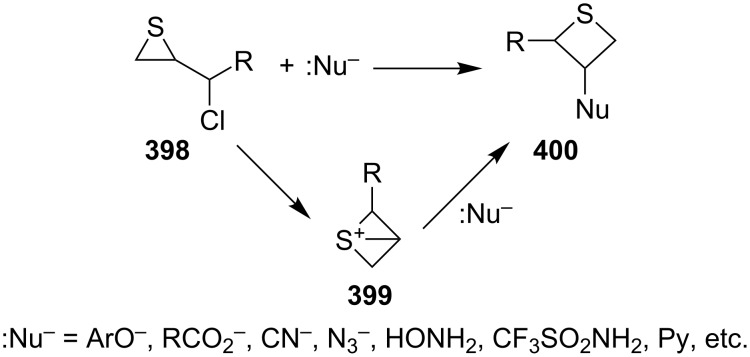
Synthesis of 3-substituted thietanes **400** from (1-chloroalkyl)thiiranes **398**.

**4.1.1 Synthesis via nucleophilic ring expansion of 2-(1-haloalkyl)thiiranes:** A thiirane–thietane rearrangement took place upon the interaction of (1-haloaklyl)thiiranes **398** with hard and weak nucleophiles (:Nu^−^) in the presence of a base. It was an efficient method for the preparation of 3-substituted thietanes **400** from (1-haloalkyl)thiiranes **398** through an intramolecular nucleophilic substitution followed by an intermolecular nucleophilic displacement with the in-situ generated 1-thiabicyclo[1.1.0]butan-1-iums **399** as key intermediates. Following this route, 3-substituted thietanes **400** were prepared from reactions of 2-(1-chloroalkyl)thiiranes **398**, especially chloromethylthiirane (epithiochlorohydrin, **398a**), with hard and weak nucleophiles [105–109], including phenoxides [105], carboxylates and dicarboxylates [106–107], potassium cyanide, sodium azide, hydroxylamine, trifluoromethanesulfonamide, and pyridine [108]. However, the method could only applied to the synthesis of 3-substituted thietanes **400** from (1-chloroalkyl)thiiranes **398** (Scheme 84).

**Scheme 85:**
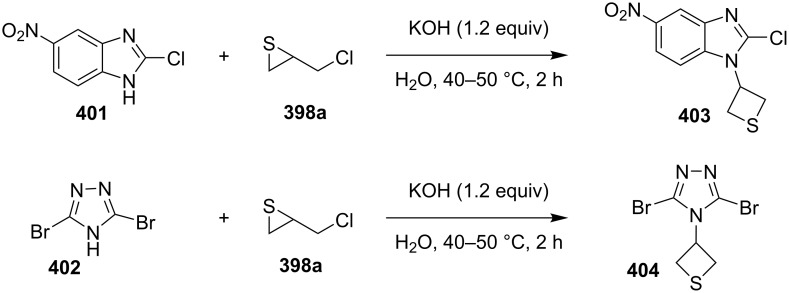
Synthesis of *N*-(thietane-3-yl)azaheterocycles **403** and **404** through reaction of chloromethylthiirane (**398a**) with benzimidazole **401** and 1,2,4-triazole **402**.

Nitrogen-containing aromatic heterocycles, such as 2-chloro-5(6)-nitrobenzimidazole (**401**) and 3,5-dibromo-1,2,4-triazole (**402**), were used as nucleophiles in the reaction with chloromethylthiirane (**398a**) giving rise to 3-heteroarylthietanes **403** and **404**, respectively [109–110] (Scheme 85).

**Scheme 86:**
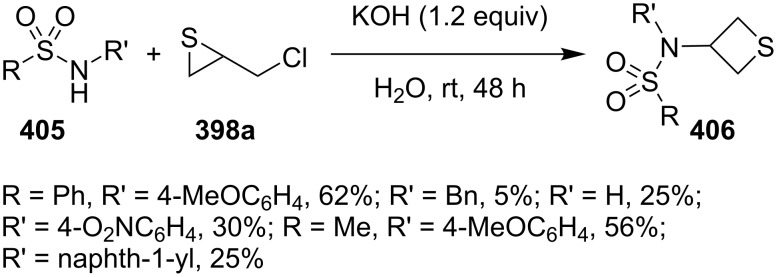
Synthesis of 3-sulfonamidothietanes **406** from sulfonamides and chloromethylthiirane (**398a**).

The treatment of various *N*-substituted sulfonamides **405** with chloromethylthiirane (**398a**) in the presence of KOH in water gave rise to the corresponding 3-sulfonamidothietanes **406** in low to moderate yields [111] (Scheme 86).

**Scheme 87:**
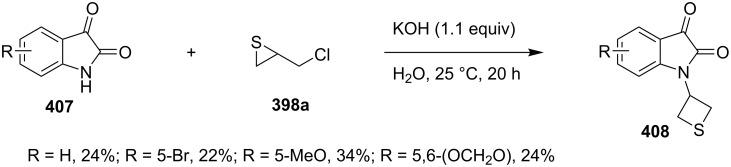
Synthesis of *N*-(thietane-3-yl)isatins **408** from chloromethylthiirane (**398a**) and isatins **407**.

Also isatins **407** reacted with chloromethylthiirane (**398a**) to afford *N-*(thietane-3-yl)isatin derivatives **408** in moderate yields [112] (Scheme 87).

**Scheme 88:**
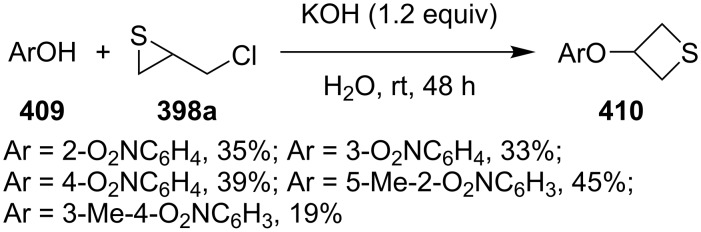
Synthesis of 3-(nitrophenyloxy)thietanes **410** from nitrophenols **409** and chloromethylthiirane (**398a**).

When weakly nucleophilic nitrophenols **409** were used as nucleophiles, the ring-expansion reaction of chloromethylthiirane (**398a**) yielded the corresponding 3-(nitrophenyloxy)thietanes **410** in only low to moderate yields under basic conditions [113] (Scheme 88).

**Scheme 89:**
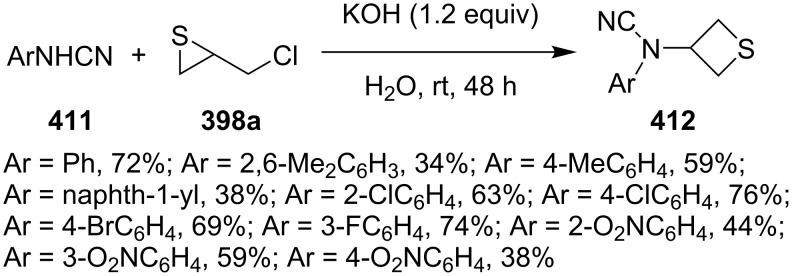
Synthesis of *N*-aryl-*N*-(thietane-3-yl)cyanamides **412** from *N*-arylcyanamides **411** and chloromethylthiirane (**398a**).

Similarly, various *N*-arylcyanamides **411** reacted with chloromethylthiirane (**398a**) under basic conditions to give the corresponding *N*-aryl-*N*-(thietane-3-yl)cyanamides **412** in moderate to good yields [113] (Scheme 89).

**Scheme 90:**
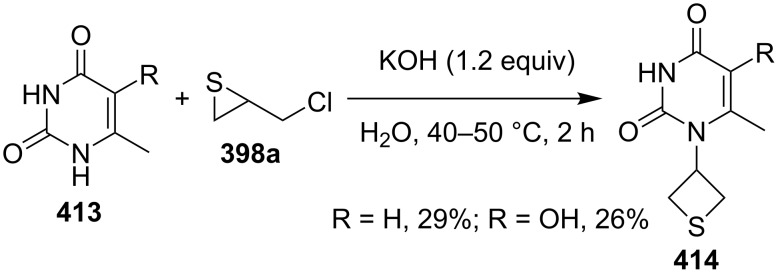
Synthesis of 1-(thietane-3-yl)pyrimidin-2,4(1*H*,3*H*)-diones **414** from chloromethylthiirane (**398a**) and pyrimidine-2,4(1*H*,3*H*)-diones.

Pyrimidine-2,4(1*H*,3*H*)-diones **413** were derivatized with chloromethylthiirane (**398a**), giving rise to 1-(thietane-3-yl)pyrimidin-2,4(1*H*,3*H*)-dione derivatives **414** in low yields [114] (Scheme 90).

**Scheme 91:**
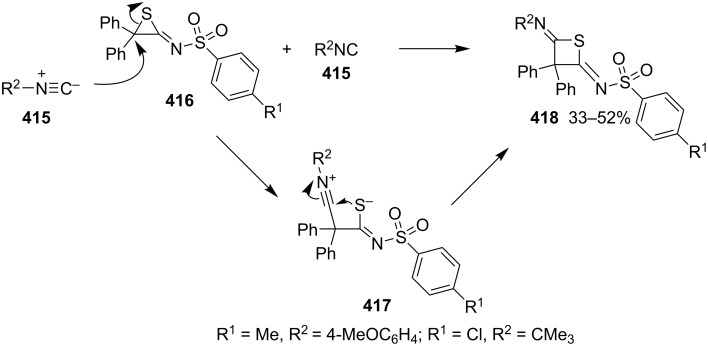
Synthesis of 2,4-diiminothietanes **418** from 2-iminothiiranes **416** and isocyanoalkanes **415**.

**4.1.2 Synthesis via nucleophilic ring expansion of thiiranes:** The nucleophilic ring expansion of thiiranes was used for the synthesis of thietanes. Isocyanoalkanes **415** can be considered as nucleophiles. However, after the nucleophilic addition, they could become electrophiles. Thus, they can be applied in the nucleophilic ring expansion of thiiranes **416**, in which the generated thiolates **417** as nucleophiles undergo a further intramolecular addition to form iminothietanes **418**. 2-Iminothiiranes **416** underwent a nucleophilic ring expansion with isocyanoalkanes **415** as nucleophiles to give rise to 2,4-diiminothietanes **418** in 33 to 52% yields [101] (Scheme 91).

**Scheme 92:**
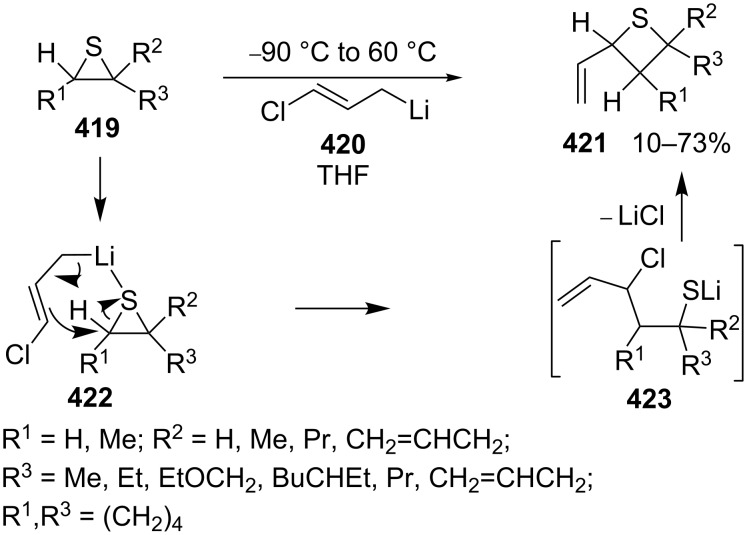
Synthesis of 2-vinylthietanes **421** from thiiranes 419 and 3-chloroallyl lithium (**420**).

3-Chloroallyl lithium (**420**) was also applied as a nucleophile to synthesize 2-vinylthietanes **421** in the electrophilic ring expansion of thiiranes **419**. The corresponding thiiranes **419** reacted with 3-chloroallyl lithium (**420**), yielding vinylthietanes **421** in 10–73% yields. This was a general route towards the synthesis of 2-vinylthietanes **422**. From a mechanistic point of view, 3-chloroallyl lithium (**420**) first coordinated with the thiiranes **419** followed by a nucleophilic ring opening and intramolecular substitution [115] (Scheme 92).

One carbon-containing nucleophiles with a good leaving group should be another reagent for the nucleophilic ring expansion of thiiranes. In the ring expansion, the nucleophiles first nucleophilically open the thiiranes and the generated thiolates then serve as nucleophiles to undergo a further intramolecular displacement to give the thietanes. Dimethyloxosulfonium methylide was demonstrated to be a suitable reagent for the nucleophilic ring expansion of three-membered heterocycles. It was successfully applied in the preparation of oxetanes and azetidines via the ring expansions of oxiranes [116–118] and aziridines [119–120]. However, both thiiranes and thietanes were less stable than the corresponding oxa and aza-analogs.

**Scheme 93:**
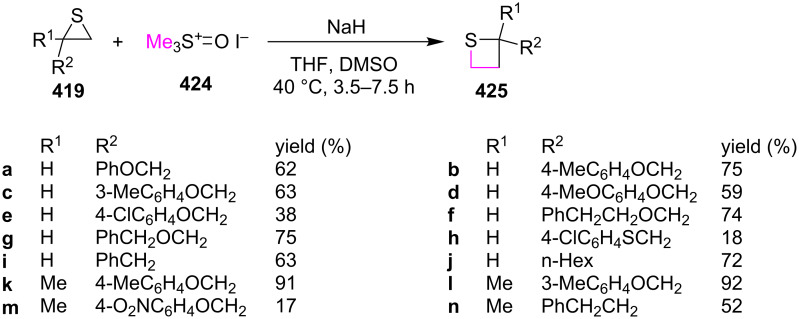
Synthesis of thietanes from thiiranes **419** and trimethyloxosulfonium iodide **424**.

Thiiranes **419** were readily prepared from the corresponding oxiranes [121–123]. The ring expansion reactions of trimethyloxosulfonium iodide (**424**) and various thiiranes **419** delivered the corresponding thietanes **425** in the presence of NaH in a mixture of THF and DMSO at 40 °C [22] (Scheme 93).

**Scheme 94:**
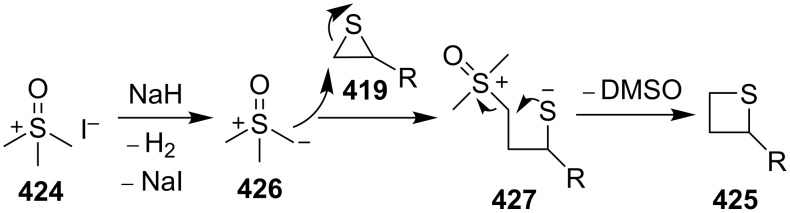
Mechanism for synthesis of thietanes **425** from thiiranes **419** and trimethyloxosulfonium iodide **424**.

The reaction mechanism was proposed as following. The treatment of trimethyloxosulfonium iodide (**424**) with sodium hydride generated dimethyloxosulfonium methylide (**426**) as the one carbon-containing nucleophile with DMSO (**379**) as a good leaving group. The nucleophilic attack of **426** on thiiranes **419** from the least substituted ring carbon atom generated the zwitterionic intermediates **427**, with a good regioselectivity following the general regioselectivity rule in nucleophilic ring opening reactions of aliphatic three-membered heterocycles [124–132]. The generated thiolate in the zwitterionic intermediates **427** then further underwent an intramolecular nucleophilic substitution to yield the desired thietanes **425** by loss of a molecule of DMSO [22] (Scheme 94).

**4.1.3 Synthesis via electrophilic ring expansion of thiiranes:** To realize the synthesis of functionalized thietanes, electron-deficient sulfur ylides were investigated in the ring expansion of thiiranes. However, the reactions failed due to the poor nucleophilicity of the electron-deficient sulfur ylides. However, in the presence of rhodium catalysts, the electron-deficient sulfur ylides were converted into electrophilic metallocarbenes, which favorably reacted with the electron-rich sulfur atom in the thiiranes and further underwent an electrophilic ring expansion to afford thietanes.

**Scheme 95:**
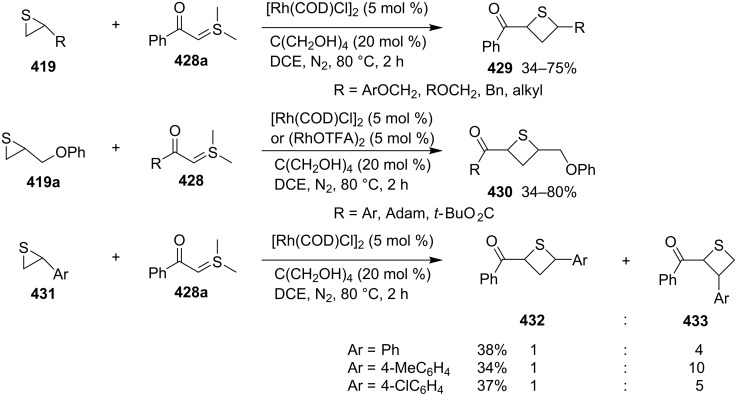
Synthesis of functionalized thietanes from thiiranes and dimethylsulfonium acylmethylides.

Dimethylsulfonium acylmethylides **428** reacted with 2-alkylthiiranes **419** to produce 2-acyl-4-alkylthietanes **429** and **430** in moderate to good yields. However, they gave rise to mixtures of 2-acyl-4-arylthietanes **432** and 2-acyl-3-arylthietanes **433** in ratios between 1:4 to 1:10 in the reactions with 2-arylthiiranes **431** [23] (Scheme 95).

**Scheme 96:**
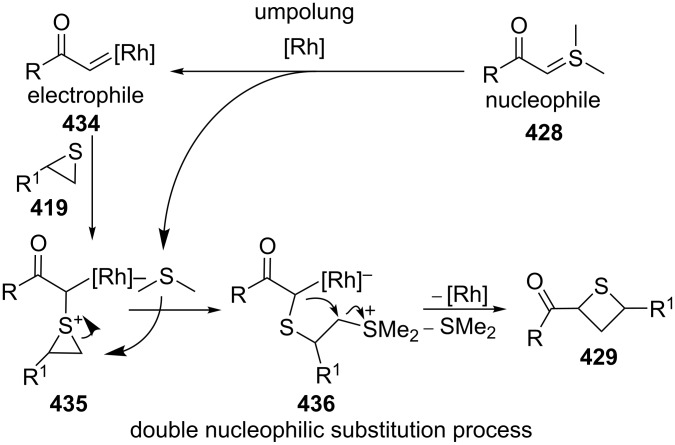
Mechanism for the rhodium-catalyzed synthesis of functionalized thietanes **429** from thiiranes **419** and dimethylsulfonium acylmethylides.

The reaction mechanism was proposed as following. The nucleophilic acyl sulfur ylides **428** first reacted with the rhodium catalyst to generate the electrophilic metallocarbenes **434** by loss of dimethyl sulfide, realizing an umplung. Thiiranes **419** then reacted nucleophilically with the electrophilic metallocarbenes **434** to yield thiiranium intermediates **435**, which were nucleophilically attacked by the released dimethyl sulfide, producing the ring-opened zwitterionic intermediates **436**. The intermediates **436** further underwent an intramolecular substitution, affording the desired thietanes **429** by loss of dimethyl sulfide and the rhodium catalyst. In this transformation dimethyl sulfide worked as a transient nucleophile and leaving group in the reaction system [23] (Scheme 96).

**Scheme 97:**
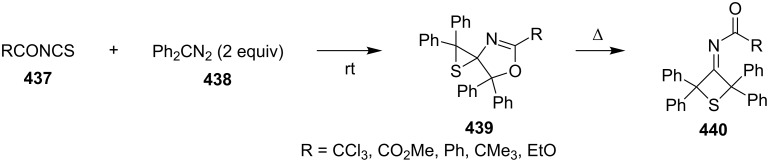
Synthesis of 3-iminothietanes **440** through thermal isomerization from 4,5-dihydro-1,3-oxazole-4-spiro-2'-thiiranes **439**.

**4.1.4 Synthesis via thermal expansion reaction of spirooxazoline-thiiranes:** Acyl isothiocyanates (RCONCS, **437**) reacted with two equivalents of diphenyldiazomethane (**438**) at room temperature to give 4,5-dihydro-1,3-oxazole-4-spiro-2'-thiiranes **439**, which isomerized thermally to 3-iminothietanes **440** [133–135] (Scheme 97).

#### 4.2 Synthesis via ring contraction reactions

**Scheme 98:**
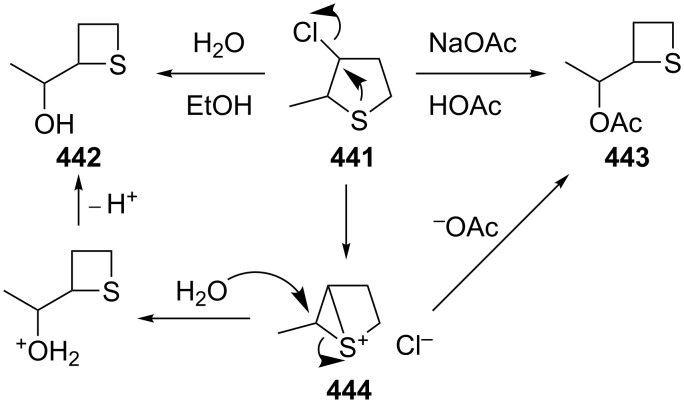
Synthesis of thietanes **443** from 3-chloro-2-methylthiolane (**441**) through ring contraction.

**4.2.1 Synthesis through the ring contraction of thiolanes:** Compared to the ring expansion reactions of thiiranes to thietanes, the ring contraction of thiolanes to thietanes was applied in only limited cases. As an example, 3-chloro-2-methylthiolane (**441**) underwent a ring contraction to give 2-(1-hydroxyethyl)thietane (**442**) and 2-(1-acetoxyethyl)thietane (**443**), respectively, when it was treated with water in ethanol or sodium acetate in acetic acid. The ring contraction proceeded through a thiiranium intermediate **444**, which was isolated as chloride salt from the reaction system, indicating that an intramolecular nucleophilic substitution occurred, followed by the nucleophilic ring opening of the thiiranium ring [32] (Scheme 98).

**Scheme 99:**
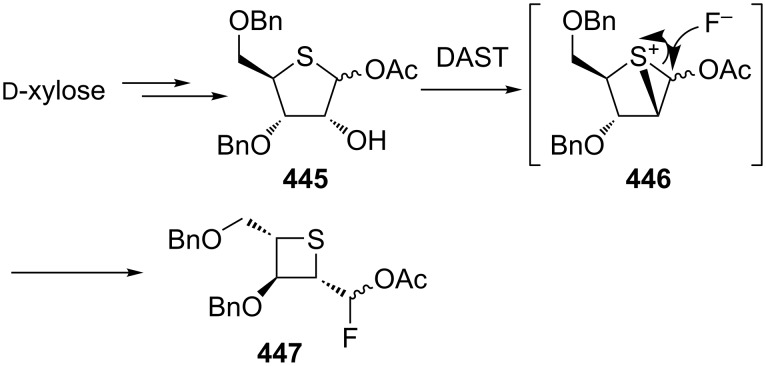
Synthesis of an optically active thietanose **447** from D-xylose involving a ring contraction.

The ring contraction of thiolanes to thietanes was also utilized in the synthesis of thietanoses. The ring contraction was realized by the DAST-mediated conversion of thiofuranose **445** derived from D-xylose into the protected fluorinated thietanose **447** through a thiiranium intermediate **446** [135–136] (Scheme 99).

**Scheme 100:**
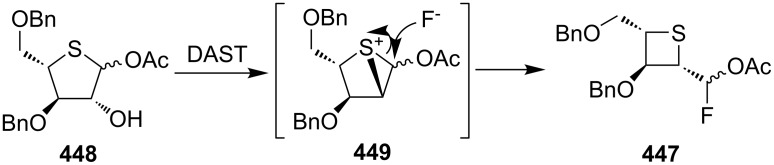
Synthesis of optically thietane **447** via the DAST-mediated ring contraction of **448**.

The similar DAST-mediated ring contraction of thiopentose **448** to thiotetraose **447** was also reported [20] (Scheme 100).

**Scheme 101:**
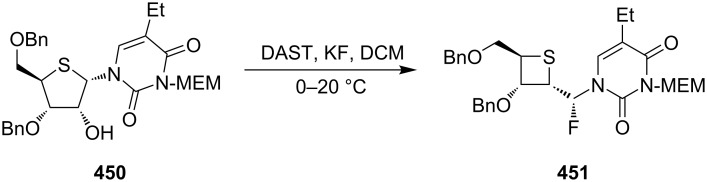
Synthesis of the optically thietane nucleoside **451** via the ring contraction of thiopentose in **450**.

The DAST-mediated ring contraction of a thiopentose to a thiotetraose was realized in the direct conversion of the thiopentose in thionucleoside **450** to its thiotetraose analogue **451** [20] (Scheme 101).

**Scheme 102:**
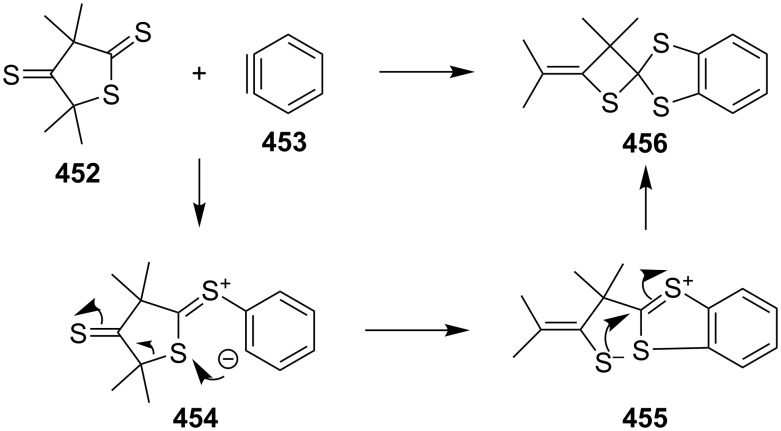
Synthesis of spirothietane **456** from 3,3,5,5-tetramethylthiolane-2,4-dithione (**452**) and benzyne (**453**).

The reaction of 3,3,5,5-tetramethylthiolane-2,4-dithione (**452**) with benzyne (**453**) gave a spirothietane-benzodithiole **456** in a good yield. In the transformation, the thiocarbonyl group of the dithioester in thiolane-2,4-dithione **452** initially attacked benzyne (**453**) to afford a betaine **454**, which finally rearranged to give the spirothietane-benzodithiole **456** [137] (Scheme 102).

**Scheme 103:**
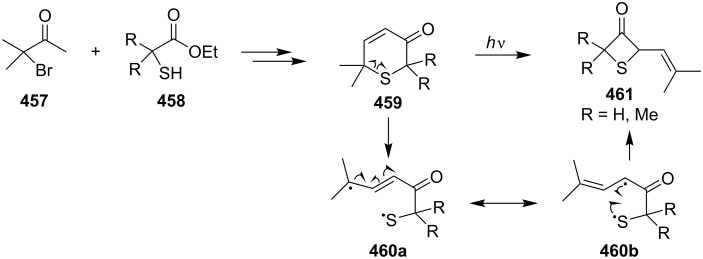
Synthesis of thietanes **461** via photoisomerization of 2*H*,6*H*-thiin-3-ones **459**.

**4.2.2 Synthesis via the ring contraction of 2*H*,6*H*-thiin-3-ones:** 2*H*,6*H*-Thiin-3-ones **459** were first generated from 3-bromo-3-methylbutan-2-one (**457**) and mercapto esters (R_2_C(SH)CO_2_Et, **458**) in four steps. Upon UV irradiation (350 nm) in either MeCN, benzene or Me_2_CHOH solution, these newly synthesized heterocycles **459** isomerized efficiently to 2-(1-alkenyl)thietan-3-ones **461**. The rearrangement was assumed to proceed via an excited-singlet state and sulfuranyl-alkyl biradicals **460** formed by bonding of C(α) of the enone C=C bond on sulfur as possible intermediates [21] (Scheme 103).

### 5. Phosphorothioate-mediated synthesis

#### 5.1. Synthesis from enones

**Scheme 104:**
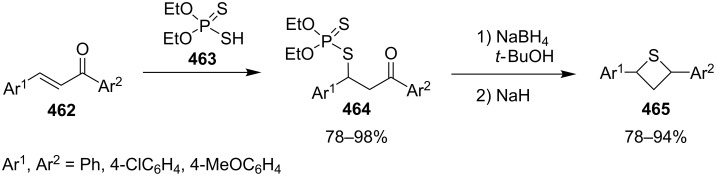
Phosphorodithioate-mediated synthesis of 1,4-diarylthietanes **465**.

In 1981, Ueno and co-workers were the first who utilized *O,O*-diethyl hydrogen phosphorodithioate (**462**) as a nucleophile in the Michael addition of chalcones **462**, affording *O,O*-diethyl *S*-(1,3-diaryl-3-oxopropyl)phosphorodithioates **464**, which were further reduced with sodium borohydride and treated with sodium hydride to give rise to 2,4-diarylthietanes **465** [138] (Scheme 104).

**Scheme 105:**
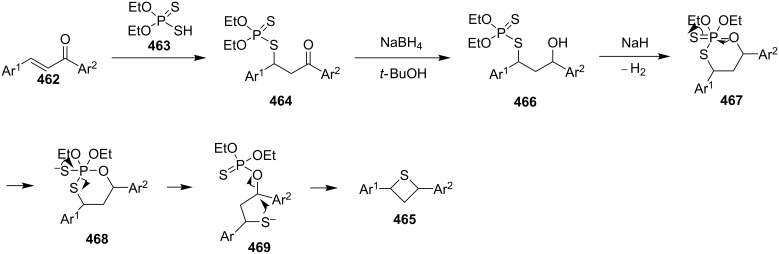
Mechanism of the phosphorodithioate-mediated synthesis of 1,4-diarylthietanes **465**.

In this reaction, the *O,O*-diethyl *S*-(1,3-diaryl-3-oxopropyl)phosphorodithioates **464** were converted to the corresponding alkoxides **467** after the reduction with sodium borohydride and the treatment with sodium hydride. The alkoxides **467** underwent an intramolecular nucleophilic addition to the phosphorus atom followed by an elimination and an intramolecular substitution to give rise to 2,4-diarylthietanes **465**. In this strategy, *O,O*-diethyl hydrogen phosphorodithioate (**463**) first worked as a nucleophile to introduce a sulfur atom in the substrates followed by its conversion to *O,O,O*-trialkylphosphorothioate **469** as a leaving group in the final nucleophilic substitution [138] (Scheme 105).

**Scheme 106:**
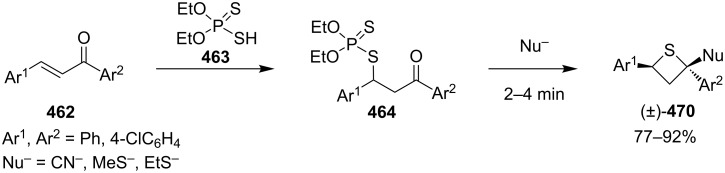
Phosphorodithioate-mediated synthesis of trisubstituted thietanes (±)-**470**.

In 2002, when Yadav worked independently, he further developed Ueno’s synthetic strategy. He and his co-worker treated *O,O*-diethyl *S*-(1,3-diaryl-3-oxopropyl)phosphorodithioates **464** with nucleophiles, such as cyanide, methanethiolate, and ethanethiolate, in the solid state under microwave irradiation affording the 2-functionalized 2,4-diarylthietanes **470** [139] (Scheme 106).

**Scheme 107:**
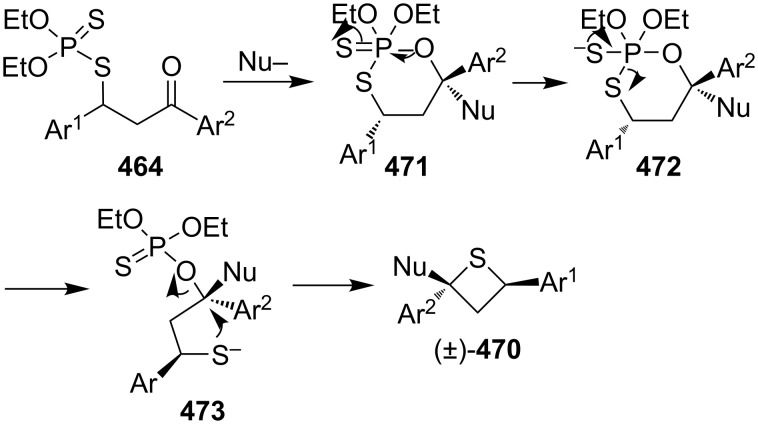
Mechanism on the phosphorodithioate-mediated synthesis of trisubstituted thietanes.

In this reaction, the nucleophiles attacked the carbonyl group of *O,O*-diethyl *S*-(1,3-diaryl-3-oxopropyl)phosphorodithioates **464** to generate the corresponding alkoxides **471**. Compounds **471** then underwent an intramolecular nucleophilic addition to phosphorus followed by an elimination and an intramolecular substitution to give rise to 2-functionalized 2,4-diarylthietanes **470** [139] (Scheme 107).

**Scheme 108:**
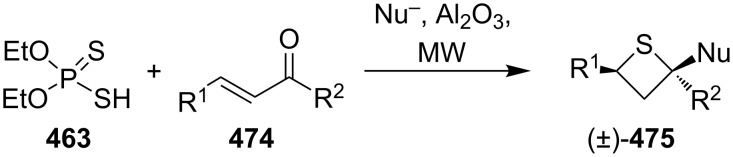
Phosphorodithioate-mediated synthesis of thietanes (±)-**475**.

In 2011, Myrboth and co-workers mentioned a similar transformation. The reaction of *O,O*-diethyl hydrogen phosphorodithioate (**463**) and α,β-alkenones **474** was applied for the synthesis of 2,4-disubstituted thietanes **475** under microwave conditions. In this reaction, the nucleophile-induced cyclization of the Michael adducts in the presence of *O,O*-diethyl hydrogen phosphorodithioate (**463**) was realized in an alumina bath [140] (Scheme 108).

#### 5.2. Synthesis from electron-deficient olefins

**Scheme 109:**
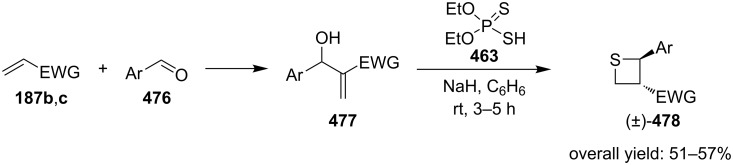
Phosphorodithioate-mediated synthesis of 1,2-disubstituted thietanes from aldehydes **476** and acrylonitrile (**187b**) and methyl acrylate (**187c**).

Yadav and co-worker first realized the synthesis of functionalized thietanes **478** from *O,O*-diethyl hydrogen phosphorodithioate (**463**), aromatic aldehydes **476**, and electron-deficient olefins (acrylonitrile (**187b**) and methyl acrylate (**187c**)). They first conducted a Baylis–Hillman reaction to prepare the Baylis–Hillman adducts **477** of aromatic aldehydes **476** with acrylonitrile (**187b**) and methyl acrylate (**187c**), and then cyclized the adducts **477** with *O,O*-diethyl hydrogen phosphorodithioate (**463**) in the presence of two equivalents of sodium hydride to afford the functionalized thietanes **478** [141] (Scheme 109).

**Scheme 110:**
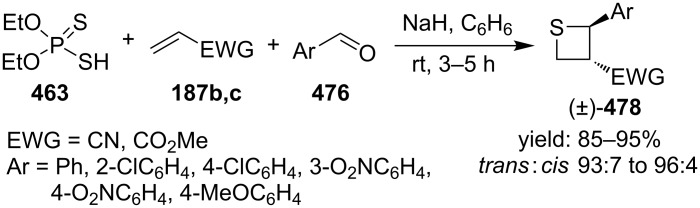
Phosphorodithioate-mediated synthesis of 1,2-disubstituted thietanes via a one-pot three-component coupling reaction.

To make the strategy more efficient, the same group developed a one-pot protocol. The one-pot three-component coupling reaction of *O,O*-diethyl hydrogen phosphorodithioate (**463**), aromatic aldehydes **476**, and electron-deficient olefins **187b,c** proofed as efficient method for the highly diastereoselective synthesis of functionalized thietanes **478** in high yields [141] (Scheme 110).

**Scheme 111:**
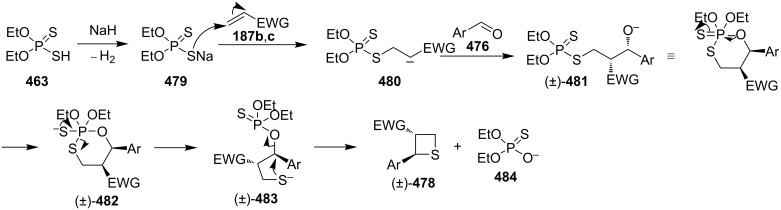
Mechanism for the phosphorodithioate-mediated synthesis of 1,2-disubstituted thietanes via three-component coupling reactions.

Mechanistically, the reaction started with the Michael addition of phosphorodithioate (**479**) and acrylonitrile/acrylate (**187b**/**187c**) to generate the resonance-stabilized carbanions **480**. The latter attacked aldehydes **476** to give the alkoxide anions **481** that underwent an intramolecular addition and elimination to generate thiolates **483**. The thiolates then afforded the desired thietane products **478** after intramolecular substitution [141] (Scheme 111).

**Scheme 112:**
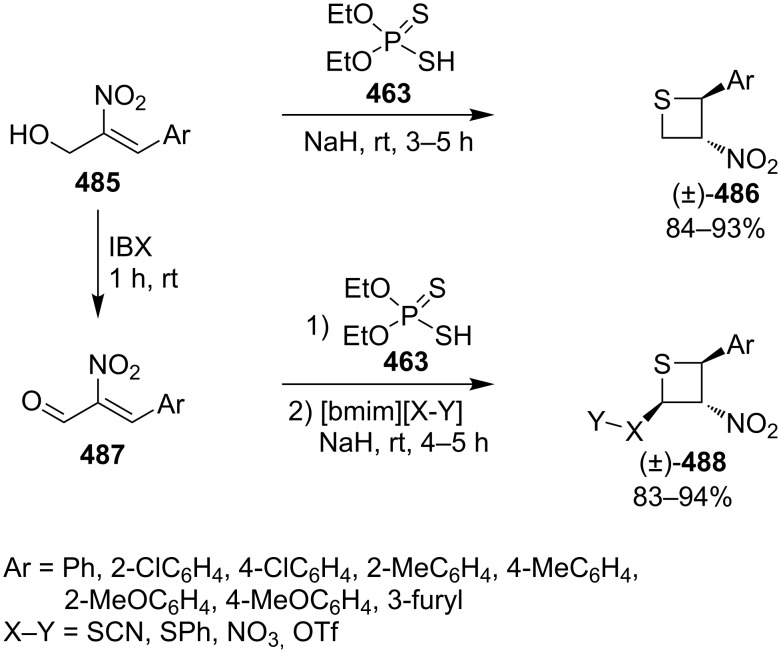
Phosphorodithioate-mediated synthesis of substituted 3-nitrothietanes.

In 2012, Yadav and co-worker reported the synthesis of 3-nitrothietanes **486** from the Baylis–Hillman adducts **485** of nitroolefins and *O,O*-diethyl hydrogen phosphorodithioate (**463**). The reaction of *O,O*-diethyl hydrogen phosphorodithioate (**463**) and 3-aryl-2-nitropropenols **485** gave rise to *trans*-2-aryl-3-nitrothietanes **486** in the presence of sodium hydride [142] (Scheme 112).

The Baylis–Hillman alcohols **485** could also be oxidized to 3-aryl-2-nitropropenals **487** with IBX as an oxidant. They were then converted to 2,3,4-trisubstituted thietanes **488** after the treatment with *O,O*-diethyl hydrogen phosphorodithioate (**463**) followed by [bmim][X-Y] in the presence of sodium hydride [142] (Scheme 112).

**Scheme 113:**
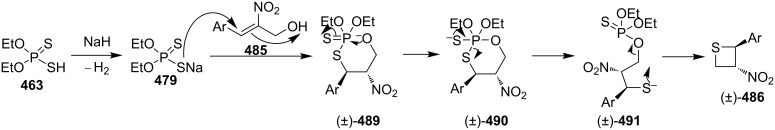
Mechanism on the phosphorodithioate-mediated synthesis of 1,2-disubstituted thietanes (±)-**486**.

For the formation mechanism, *O,O*-diethyl phosphorodithioate (**479**) nucleophilically attacked the 3-aryl-2-nitropropenals **485** to generate alkoxides **489**, which underwent an intramolecular addition and elimination followed by an intramolecular substitution to afford *trans*-2-aryl-3-nitrothietanes **486** as the product [142] (Scheme 113).

**Scheme 114:**
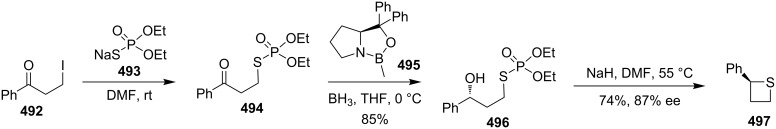
Asymmetric synthesis of (*S*)-2-phenylthietane (**497**).

Wu and Robertson realized the first asymmetric synthesis of (*S*)-2-phenylthietane (**497**) through a similar phosphorothioate-mediated strategy. They first prepared *O,O*-diethyl *S*-(3-oxo-3-phenylpropyl) phosphorothioate (**494**) from 3-iodo-1-phenylpropan-1-one (**492**) and sodium *O,O*-diethyl phosphorothioate (**493**). After an asymmetric borane reduction and the treatment with sodium hydride, (*S*)-2-phenylthietane (**497**) was obtained in 74% yield with 87% ee via the similar cyclization step [143] (Scheme 114).

**Scheme 115:**
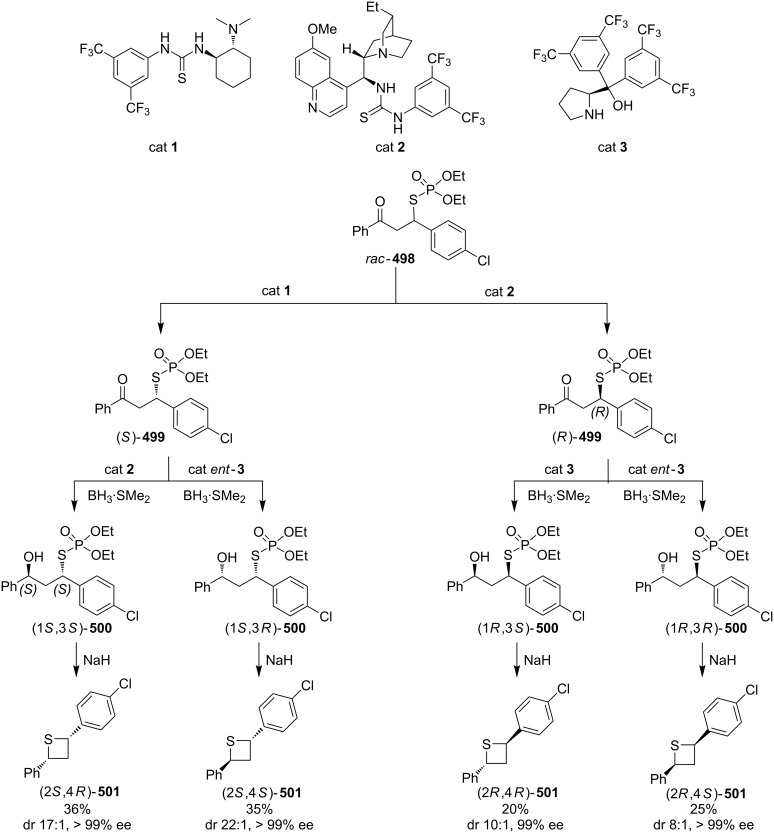
Asymmetric synthesis of optically active 2,4-diarylthietanes.

In 2016, Soós and co-workers developed a bifunctional thiourea-catalyzed stereoablative retro-sulfa-Michael reaction of *S*-(1,3-diaryl-3-oxopropyl) *O,O*-diethyl phosphorothioates **498** under biphasic conditions, that afforded enantiomerically enriched *S*-(1,3-diaryl-3-oxopropyl) *O,O*-diethyl phosphorothioates **499**. Both enantiomeric products (*R*)- and (*S*)-**499** were obtained in up to 40% yield with up to 90% ee in the presence of different enantiomeric catalysts, cat **1** and cat **2**. After the asymmetric borane reduction under the catalysis of one of a pair of enantiomeric catalysts (cat **3** and cat *ent*-**3**) and the treatment with sodium hydride, all of four enantiomerically enriched 2,4-diarylthietanes **501** were obtained with up to 99% ee [144] (Scheme 115).

### 6. Synthesis via cyclizations

#### 6.1 Synthesis via intramolecular thioesterification

**Scheme 116:**
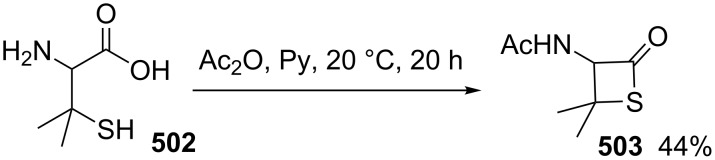
Synthesis of 3-acetamidothietan-2-one **503** via the intramolecular thioesterification of 3-mercaptoalkanoic acid **502**.

2-Amino-3-mercapto-3-methylbutanoic acid (**502**), penicillamine, was converted into the corresponding thietan-2-one derivative **503** with acetic anhydride as a coupling reagent in pyridine accompanied by *N*-acetylation [145] (Scheme 116).

**Scheme 117:**
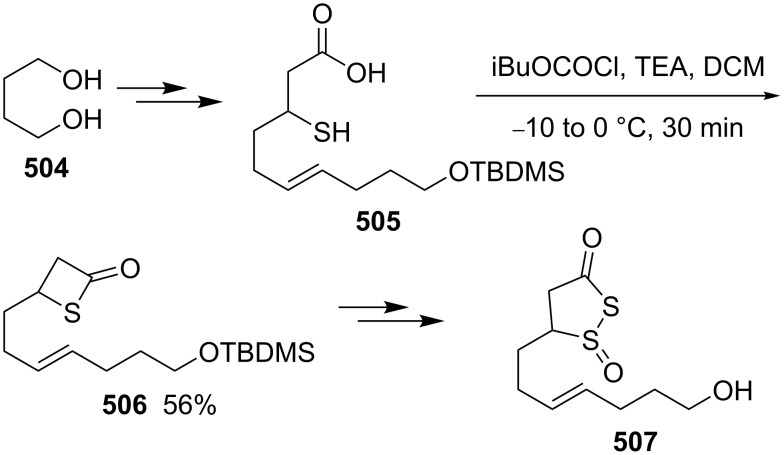
Synthesis of 4-substituted thietan-2-one via the intramolecular thioesterification of 3-mercaptoalkanoic acid.

Similarly, Pattenden and Shuker cyclized 3-mercapto-3-methylbutanoic acid into 4,4-dimethylthietan-2-one for the synthesis of antitumor antibiotic Leinamycin [146–147]. Leinamycin (LNM) is a new antitumor antibiotic produced by *Streptomyces atroolivaceus* S-140. For its preparation, its important fragment was first synthesized from butane-1,4-diol (**504**) as starting material. 3-Mercapto carboxylic acid **505** as a key intermediate was cyclized with isobutyl chloroformate as a coupling reagent, affording the thietan-2-one derivative **506** [147–148] (Scheme 117).

**Scheme 118:**
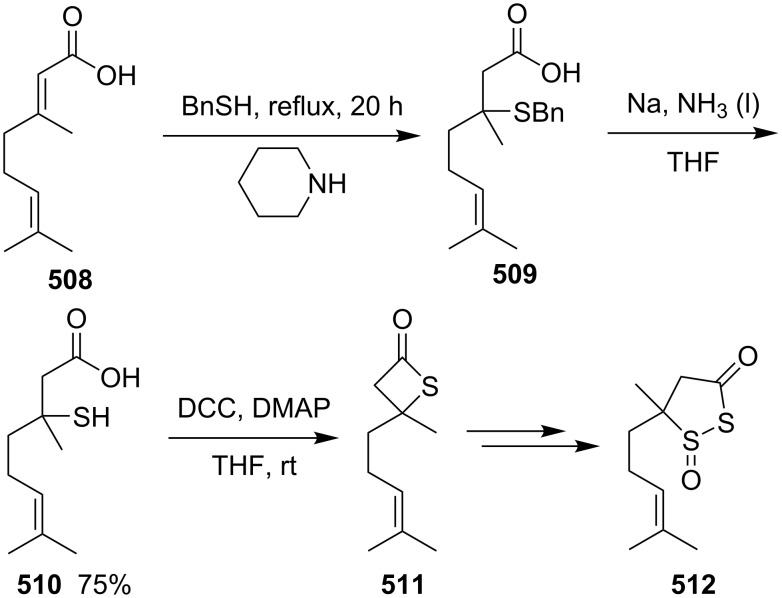
Synthesis of 4,4-disubstituted thietan-2-one **511** via the intramolecular thioesterification of the 3-mercaptoalkanoic acid **510**.

In 2013, Gates’s group prepared a small analogue of the anticancer natural product leinamycin. They first synthesized 3-mercapto carboxylic acid **510** as a key intermediate and then cyclized it with DCC and DMAP as coupling reagents, affording the thietan-2-one derivative **511** which was further converted into a small analogue **512** of leinamycin [149] (Scheme 118).

**Scheme 119:**
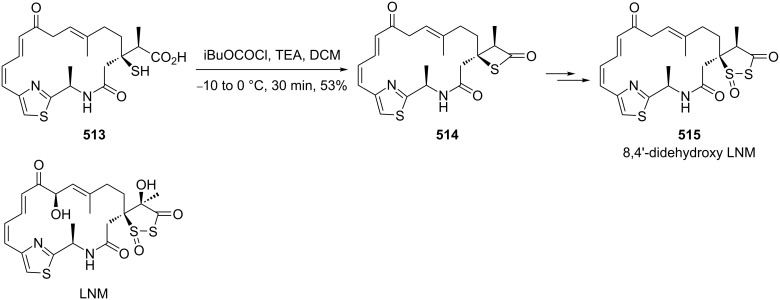
Synthesis of a spirothietan-2-one **514** via the intramolecular thioesterification of 3-mercaptoalkanoic acid.

To investigate the structure–activity relationship of leinamycin (LNM), 8,4’-didehydroxy-leinamycin (**515**) was synthesized. During the synthesis, a spirothietan-2-one intermediate **514** was prepared through an intramolecular thioesterification of 3-mercaptoalkanoic acid **513** and further transformed into the target product **515** [150] (Scheme 119).

**Scheme 120:**
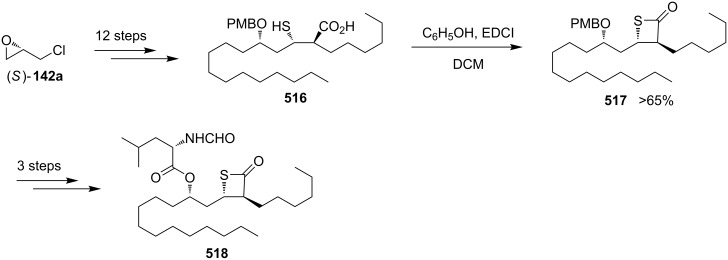
Synthesis of thiatetrahydrolipstatin starting from (*S*)-(−)-epichlorohydrin ((*S*)-**142a**).

Tetrahydrolipstatin (orlistat) is currently marketed as xenical for the treatment of obesity [151]. Crich and co-workers synthesized its sulfur analogue **518** from (*S*)-(−)-epichlorohydrin ((*S*)-**142a**). After 12 steps, 3-mercapto carboxylic acid **516** was obtained and further cyclized into a thia-β-lactone **517** in more than 65% yield with EDCI as a coupling reagent and pentafluorophenyl ester as an active ester intermediate. After 3 steps, the thia-β-lactone **517** was transformed into thiatetrahydrolipstatin **518** [152] (Scheme 120).

#### 6.2 Synthesis via the intramolecular nucleophilic substitution of 2-bromoalk-1-ene-4-thiols

**Scheme 121:**
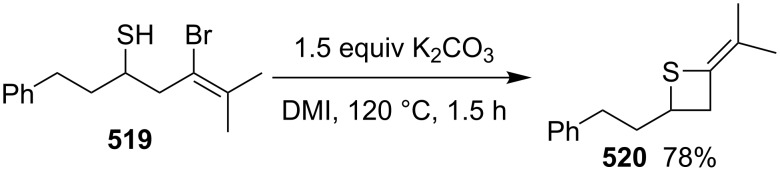
Synthesis of 2-phenethyl-4-(propan-2-ylidene)thietane (**520**) from 5-bromo-6-methyl-1-phenylhept-5-ene-3-thiol (**519**).

When Narasaka and co-workers investigated the formal intramolecular nucleophilic substitution at sp^2^ carbon centers for the preparation of oxygen, nitrogen, and sulfur-containing unsaturated five-membered heterocycles, they found that the method could be applied for the synthesis of 2-alkylidenethietanes. They obtained 2-phenethyl-4-(propan-2-ylidene)thietane (**520**) from 5-bromo-6-methyl-1-phenylhept-5-ene-3-thiol (**519**) as a substrate in 1,3-dimethyl-2-imidazolidinone (DMI) as solvent [25] (Scheme 121).

**Scheme 122:**
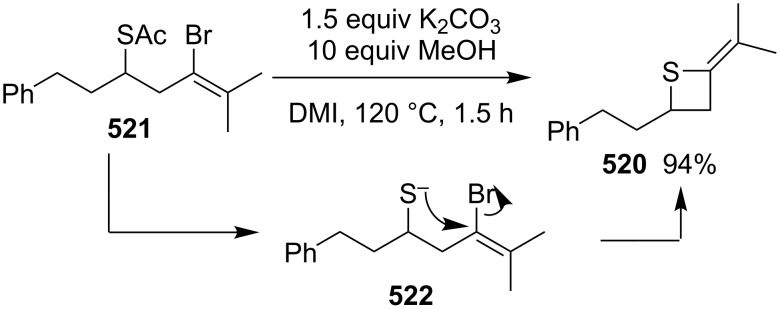
Synthesis of 2-phenethyl-4-(propan-2-ylidene)thietane (**520**) directly from *S*-(5-bromo-6-methyl-1-phenylhept-5-en-3-yl)thioacetate (**521**).

They further applied the method to synthesize 2-phenethyl-4-(propan-2-ylidene)thietane (**520**) from *S*-(5-bromo-6-methyl-1-phenylhept-5-en-3-yl)thioacetate (**521**) directly because K_2_CO_3_ led to deacetylation of the acetyl group from the thioacetate **521**, which was prepared from the corresponding alcohol and thiolacetic acid with the Mitsunobu reagent [153] (Scheme 122).

**Scheme 123:**
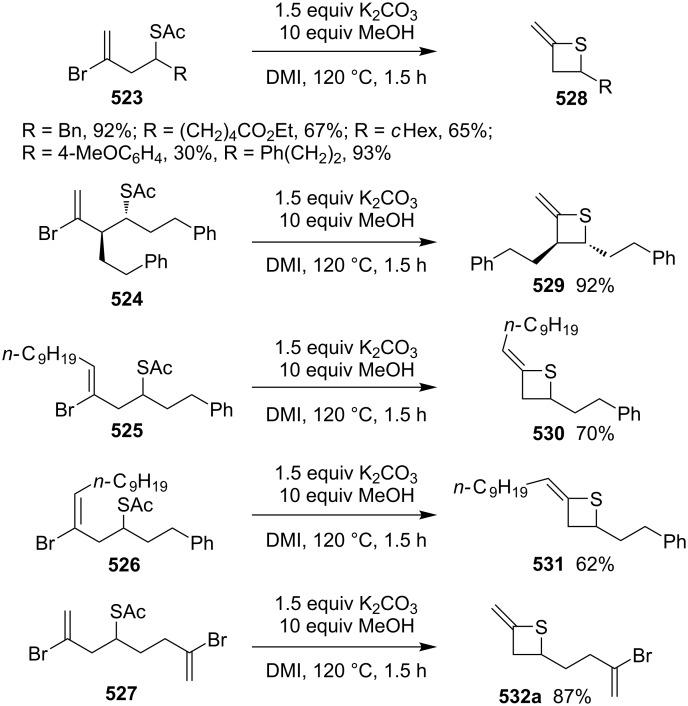
Synthesis of 2-alkylidenethietanes from *S*-(2-bromoalk-1-en-4-yl)thioacetates.

The method was applied using various substrates to synthesize a series of 2-alkylidenethietanes **528**–**532**. The *S*-(2,7-dibromoocta-1,7-dien-4-yl)thioacetate (**527**) generated the 2-methylidenethietane derivative **532** exclusively under the reaction conditions, revealing that the reaction preferred the 4-*exo* ring closure [153–154] (Scheme 123).

**Scheme 124:**
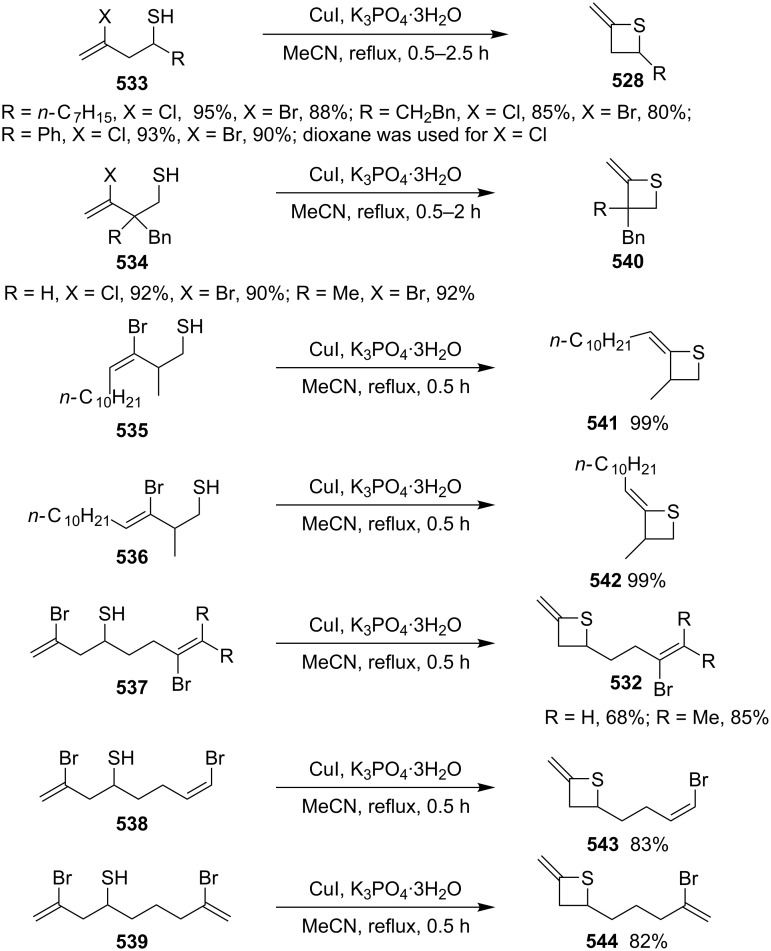
Synthesis of 2-alkylidenethietanes from *S*-(2-bromo/chloroalk-1-en-4-yl)thiols.

In 2009, Li and his co-workers developed a ligand-free CuI-catalyzed intramolecular *S*-vinylation of 2-bromo/chloroalk-1-ene-4-thiols **533**–**539** for the preparation of 2-alkylidenethietanes **528**, **532**, and **540**–**544**. They designed some substrates **537**–**539** possessing double bromovinyl moieties with different chain lengths and performed the reaction. The results indicated that the reaction preferred the 4-*exo* ring closure over other modes, such as 5-*exo*, 6-*exo*, and 6-*endo* cyclizations [155] (Scheme 124).

#### 6.3 Synthesis via nucleophilic addition

**Scheme 125:**
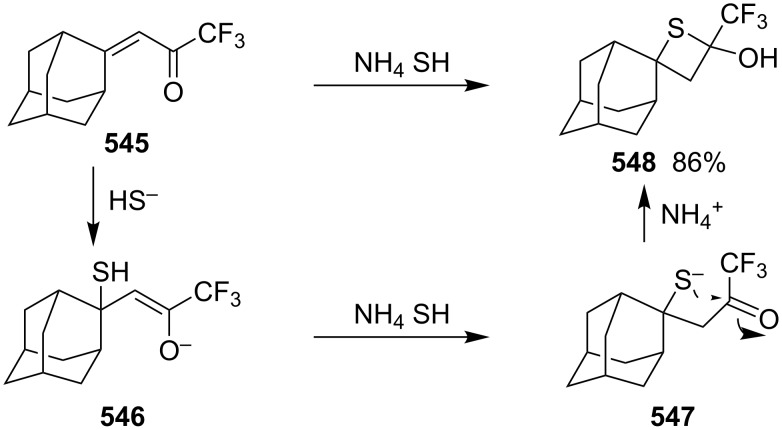
Synthesis of spirothietan-3-ol **548** from enone **545** and ammonium hydrosulfide.

The reaction of bulky α,β-unsaturated trifluoromethyl ketone, adamantylmethylene trifluoromethyl ketone (**545**), and ammonium hydrosulfide generated a spiroadamantine-thietan-3-ol **548** in 86% yield. The reaction involved a thia-Michael addition, proton transfer, and nucleophilic addition [156] (Scheme 125).

**Scheme 126:**
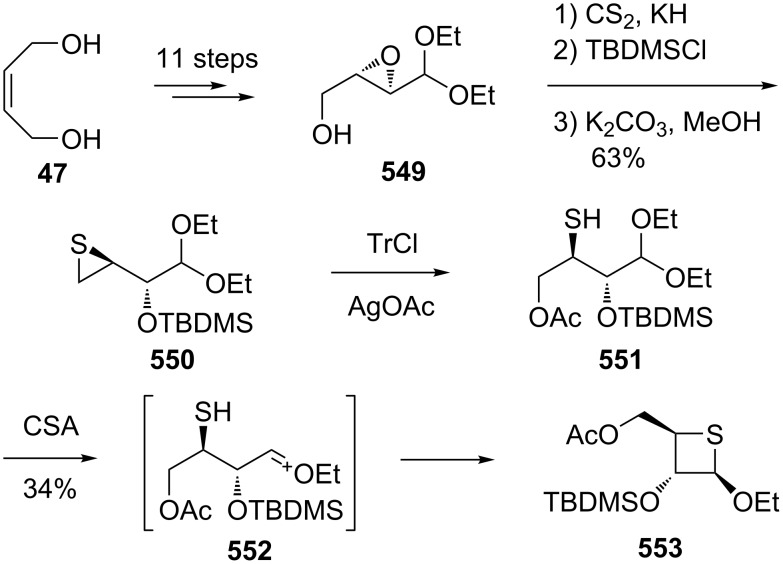
Asymmetric synthesis of the optically active thietanoside from *cis*-but-2-ene-1,4-diol (**47**).

**Scheme 127:**
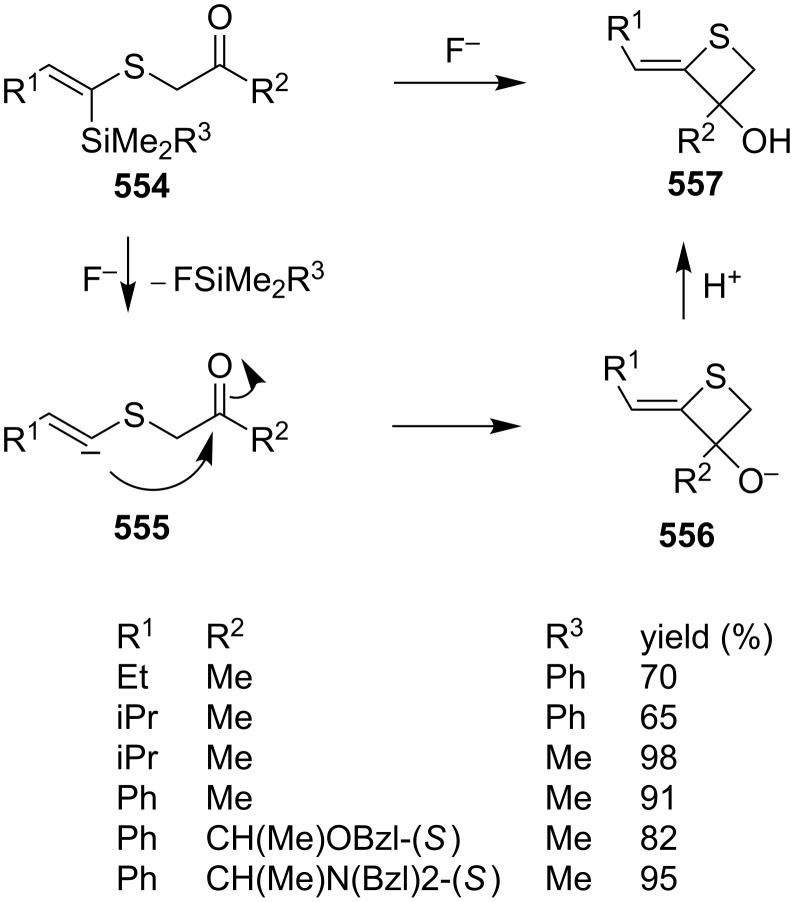
Synthesis of 2-alkylidenethietan-3-ols **557** via the fluoride-mediated cyclization of thioacylsilanes **554**.

The de novo synthesis of the enantiopure thietane derivative **553**, a four-membered ring thiosugar, was conducted from *cis*-but-2-ene-1,4-diol (**47**). The two asymmetric centers were generated first via the Sharpless asymmetric epoxidation. The epoxide **549** was then converted into the corresponding thiirane **550** through a cyclic xanthate intermediate generated by the treatment with CS_2_ and KH. After the protection of the secondary hydroxy group, methanolysis of the xanthate afforded the desired thiirane **550** in 63% overall yield. The AgOAc-mediated regioselective ring opening of the thiirane **550** provided a thiol **551**, which was converted to 1-*O*-ethyl-thietanoside **553** through the acid-catalyzed elimination of EtOH followed by the thiol nucleophilic addition induced by the treatment with CSA in refluxing benzene. The highly stereoselective conversion proceeded via an oxocarbenium intermediate **552**, leading to the thermodynamically favored *trans*,*trans*-substituted thietane derivative **553** [157] (Scheme 126).

(*Z*)-α-Silyl vinyl sulfides **554** were prepared from (*Z*)-α-silyl enethiols and chloromethyl ketones and further converted into 2-alkylidenethietan-3-ols **557** by the treatment with fluoride. The conversion included the desilylation, intramolecular nucleophilic addition, and protonation [158] (Scheme 127).

**Scheme 128:**
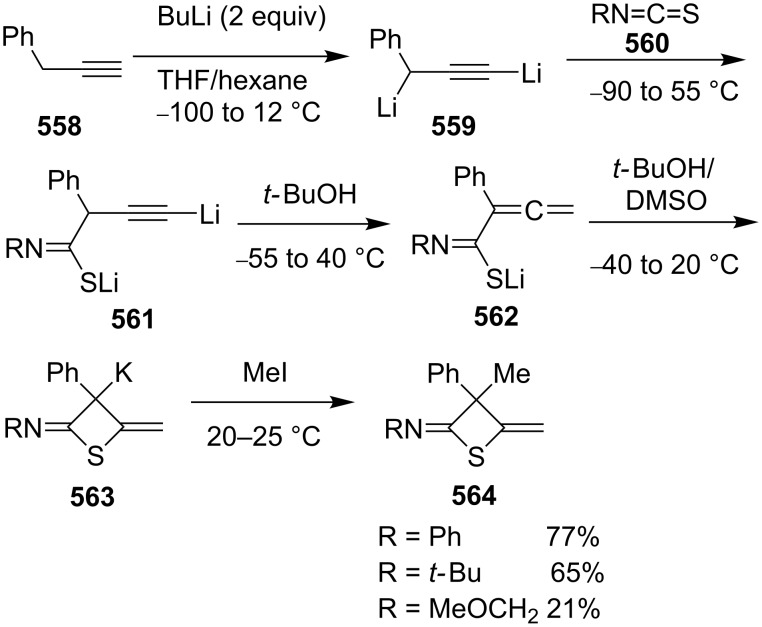
Synthesis of 2-iminothietanes via the reaction of propargylbenzene (**558**) and isothiocyanates **560** in the presence of butyllithium as strong base.

The treatment of propargylbenzene (**558**) with butyllithium generated 1,3-dilithiopropargylbenzene (**559**), which underwent a nucleophilic addition to isothiocyanates **560** followed by protonation, isomerization, intramolecular nucleophilic addition, and methylation, affording 2-iminothietane derivatives **564** [159–160] (Scheme 128).

**Scheme 129:**
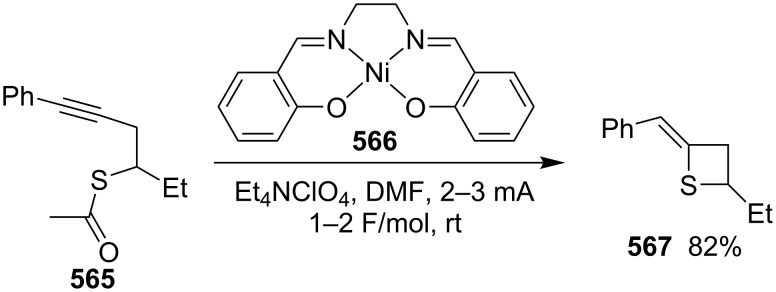
Synthesis of 2-benzylidenethietane **567** via the nickel complex-catalyzed electroreductive cyclization of 1-phenylhex-1-en-4-ylthioacetate.

One example of a 2-benzylidenethietane **567** was prepared in 82% yield from 1-phenylhex-1-en-4-ylthioacetate (**565**) via a nickel complex-catalyzed electroreduction [161] (Scheme 129). However, the electrochemical synthetic method was widely applied for the synthesis of thiacyclopetanes and thiacyclohexanes [161].

#### 6.4 Synthesis via electrocyclic reaction

**Scheme 130:**
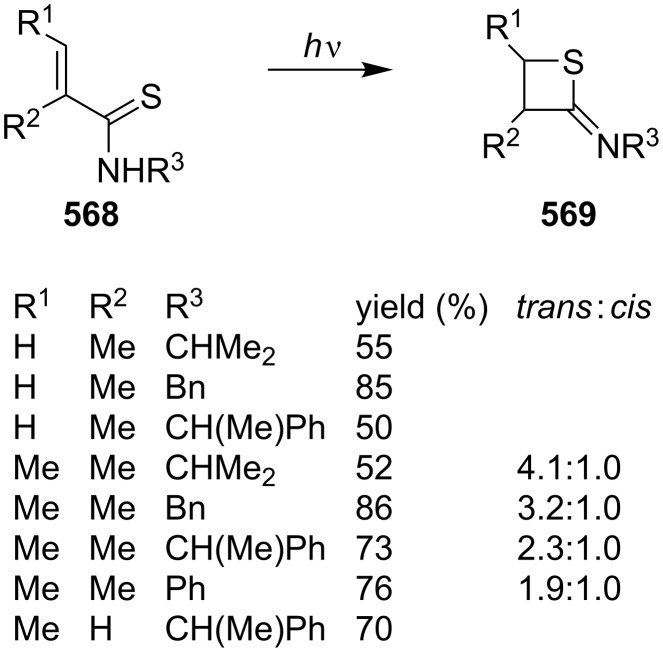
Synthesis of 2-iminothietanes **569** via the photo-assisted electrocyclic reaction of *N*-monosubstituted α,β-unsaturated thioamides.

Besides the cyclization through the inter- and intramolecular nucleophilic substitutions, the photo-assisted electrocyclic reaction of *N*-monosubstituted α,β-unsaturated thioamides **568** was also applied for the synthesis of 2-iminothietane derivatives **569** [162] (Scheme 130).

#### 6.5 Synthesis via nucleophilic addition–elimination

**Scheme 131:**
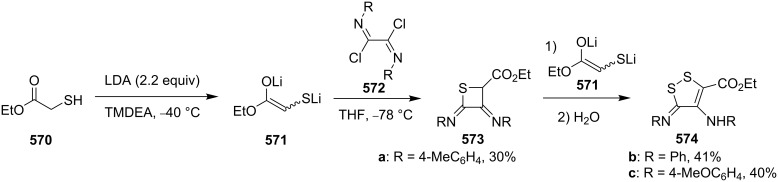
Synthesis of ethyl 3,4-diiminothietane-2-carboxylates from ethyl thioglycolate (**570**) and bis(imidoyl chloride)s.

Iminothietanes [162–169], diiminothietanes [101,170], and triiminothietanes [171] are less reported four-membered thiaheterocycles. Langer and Doring prepared ethyl 3,4-diiminothietane-2-carboxylates **573** through the cyclization of the vicinal dianion **571** generated from ethyl thioglycolate (**570**) and LDA in TMEDA with 1,2-dielectrophiles, bis(imidoyl chloride)s **572**. However, only one target diiminothietane **572a** was obtained in 40% yield (R = 4-MeC_6_H_4_). The other two reacted directly with another molecule of the dianion **571** to generate 4-amino-5-imino-1,2-dithiole-3-carboxylates **574** [172] (Scheme 131).

### 7 Miscellaneous syntheses

**Scheme 132:**
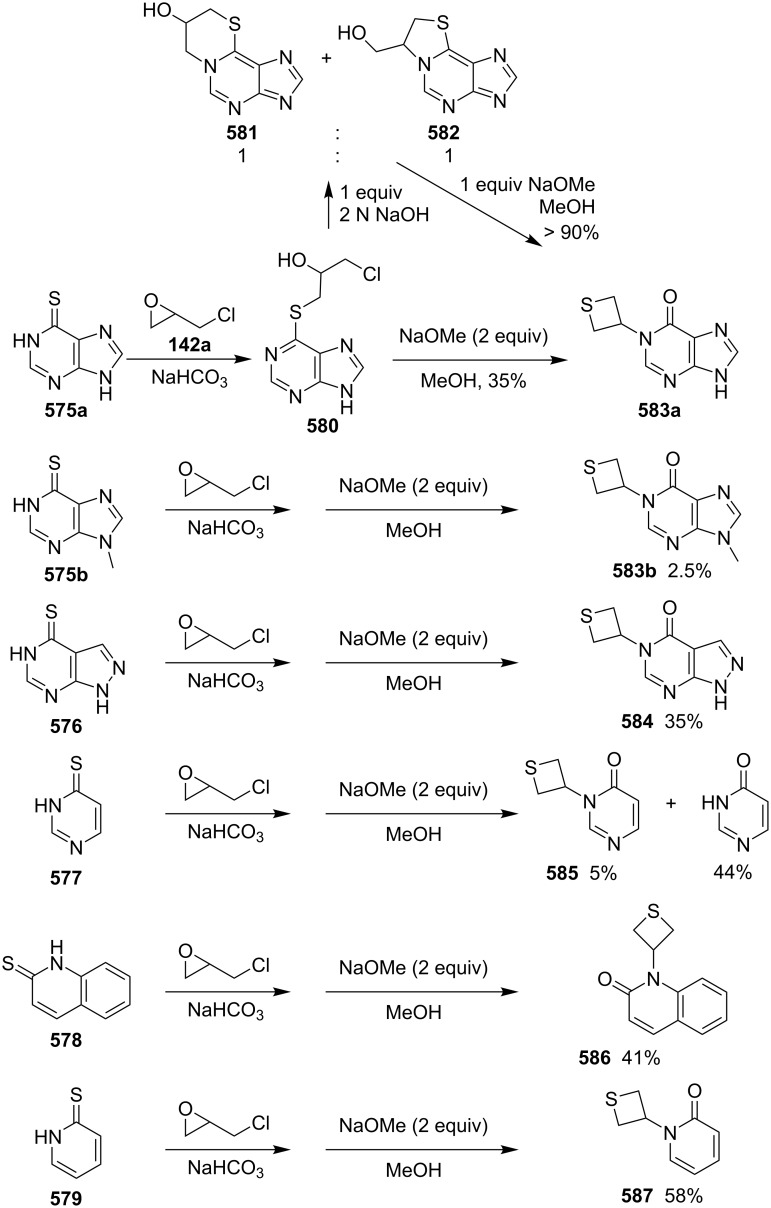
Synthesis of *N*-(thietan-3-yl)-α-oxoazaheterocycles from azaheterocyclethiones and chloromethyloxirane (**142a**).

Press and co-workers developed a rearrangement method to derivatize aromatic azaheterocyclethiones, including 1,9-dihydro-6*H*-purine-6-thiones **575**, 1,5-dihydro-4*H*-pyrazolo[3,4-*d*]pyrimidine-4(3*H*)-thione (**576**), pyrimidine-4(3*H*)-thione (**577**), quinoline-2(1*H*)-thione (**578**), and pyridine-2(1*H*)-thione (**579**), into the corresponding *N*-thietan-3-yl-α-oxo nitrogen-containing heterocycles **583**–**587** with chloromethyloxirane (**142a**) as an alkylation reagent. For the reaction process, the reaction of 1,9-dihydro-6*H*-purine-6-thione (**575a**) and chloromethyloxirane (**142a**) first generated the *S*-alkylated intermediate **580** in the presence of sodium bicarbonate. After the treatment with NaOH, the intermediate **580** converted into tricyclic intermediates **581** and **582**, which finally produced the *N*-thietan-3-yl product **583a** in more than 99% yield in methanolic sodium methoxide through a rearrangement [173] (Scheme 132).

**Scheme 133:**
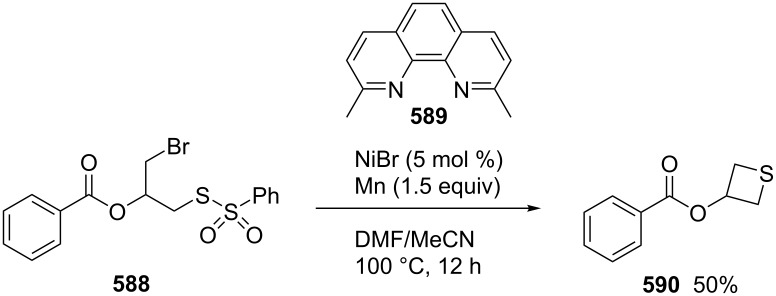
Synthesis of thietan-3-yl benzoate (**590**) via the nickel-catalyzed intramolecular reductive thiolation of *S*-(3-bromo-2-benzoyloxypropyl) benzenesulfonothioate **588**.

Recently, the nickel-catalyzed reductive thiolation of unactivated alkyl bromides and thiosulfonates was developed to synthesize thioethers. The method could also be applied in the synthesis of thietane derivatives. Such as, thietan-3-yl benzoate (**590**) was prepared through the nickel-catalyzed intramolecular reductive thiolation of *S*-(3-bromo-2-benzoyloxypropyl)benzenesulfonothioate (**588**) [174] (Scheme 133).

**Scheme 134:**
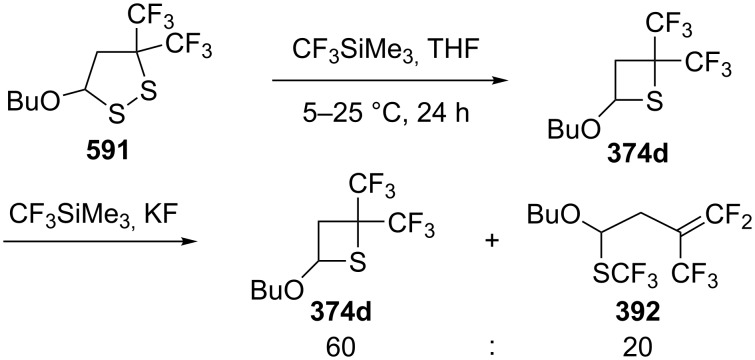
Synthesis of 2,2-bis(trifluoromethyl)thietane from 3,3-bis(trifluoromethyl)-1,2-dithiolane.

The thiophilic ring-opening reaction of 3,3-bis(trifluoromethyl)-5-butoxy-1,2-dithiolane (**591**) proceeded with the treatment of the nucleophile CF_3_SiMe_3_ to generate 2,2-bis(trifluoromethyl)-4-butoxythietane (**374d**) as an intermediate. The latter compound further reacted with another molecule of CF_3_SiMe_3_ to afford a mixture of 2,2-bis(trifluoromethyl)-4-butoxythietane (**374d**) and (1-butoxy-4,4-difluoro-3-(trifluoromethyl)but-3-en-1-yl)(trifluoromethyl)sulfane (**592**) in a ratio of 60:20 [175] (Scheme 134).

**Scheme 135:**
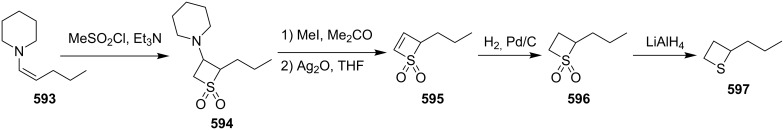
Synthesis of thietanes from enamines and sulfonyl chlorides.

The reaction of enamine **593** and methanesulfonyl chloride in the presence of triethylamine generated 3-amino-2-proylthietane 1,1-dioxide **594**. After the methylation with MeI and Hofmann elimination, 2-propyl-2*H*-thiete 1,1-dioxide (**595**) was obtained. Compound **595** was converted into 2-propylthietane (**597**) after hydrogenation and reduction [176] (Scheme 135).

**Scheme 136:**
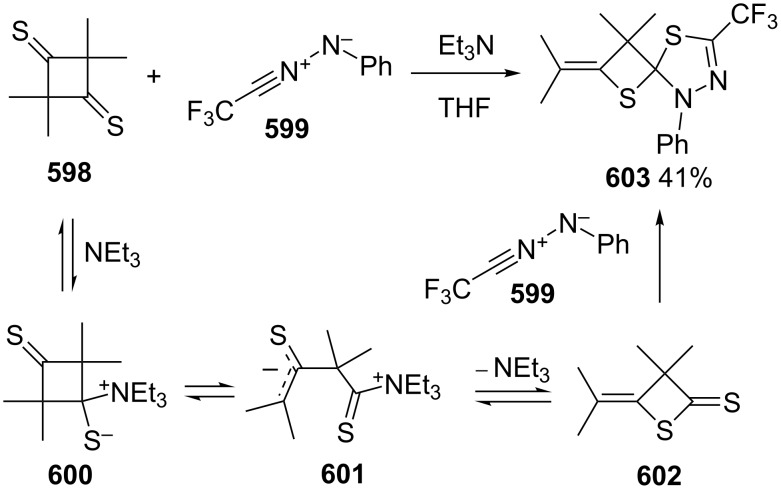
Synthesis of spirothietane **603** via the [2 + 3] cycloaddition of 2,2,4,4-tetramethylcyclobutane-1,3-dithione and nitrile imine.

It is well known that cyclobutane-1,3-dithiones undergo ring rearrangement and isomerization into thietane-2-thiones in the presence of bases [177–178]. 2,2,4,4-Tetramethylcyclobutane-1,3-dithione (**598**) generated 3,3-dimethyl-4-(propan-2-ylidene)thietane-2-thione (**602**) in the presence of triethylamine. It further reacted with the fluorinated nitrile imine **599** derived from trifluoroacetaldehyde phenylhydrazonoyl bromide in the presence of triethylamine to give 1,8-dithia-5,6-diazaspiro[3.4]oct-6-ene **603**, the spiro thietane-1,3,4-thiadiazolidine derivative, through a [2 + 3] cycloaddition [179] (Scheme 136).

**Scheme 137:**
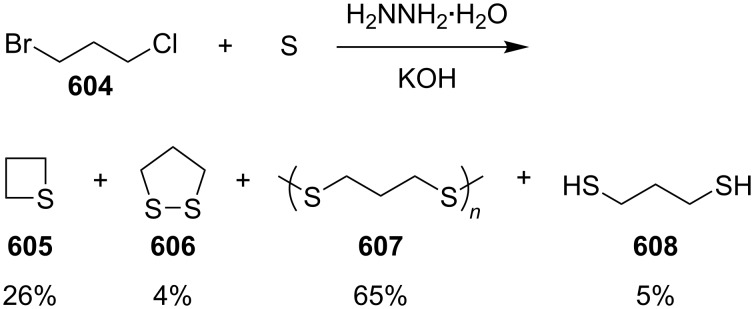
Synthesis of thietane (**605**) from 1-bromo-3-chloropropane and sulfur.

In 2006, a Russian group attempted to prepare thietane (**605**) from 1-bromo-3-chloropropane (**604**) and sulfur in the presence of hydrazine hydrate and KOH. The yield depended on the ratio of KOH:S. When the ratio was 1:2, thietane (**605**) was obtained in 26% yield, however, polymeric –(SCH_2_CH_2_CH_2_S)*_n_*– (**607**) was the major product in 65% yield [180] (Scheme 137).

## Conclusion

Thietanes are one class of important aliphatic four-membered thiaheterocycles. They are not only crucial pharmaceutical cores and structural motifs of some biological compounds, but also useful and versatile synthetic intermediates in organic chemistry. Various synthetic methods of thietanes have been developed to date. They mainly included the inter- and intramolecular nucleophilic thioetherifications and photochemical [2 + 2] cycloadditions of thiocarbonyl compounds with olefins, the ring expansions of aliphatic three-membered heterocycles and ring contractions of aliphatic five- and six-membered thiaheterocycles, the nucleophilic cyclizations, and some miscellaneous methods. Abundant synthetic methods are available for the preparation of different substituted thietanes, respectively. Although various cyclic thioetherification strategies have been applied in the synthesis of biologically important thietanose nucleosides and sulfur analogues of docetaxel and 7-deoxydocetaxel till now, it can be believed that some newly developed synthetic strategies will show wide applications in the preparation of sulfur-containing biologically active compounds and organic materials in the near future.
